# Charge density redistribution with pressure in a zeolite framework

**DOI:** 10.1038/s41598-023-28350-4

**Published:** 2023-01-28

**Authors:** Marcin Stachowicz, Roman Gajda, Agnieszka Huć, Jan Parafiniuk, Anna Makal, Szymon Sutuła, Pierre Fertey, Krzysztof Woźniak

**Affiliations:** 1 Department of Geochemistry, Mineralogy and Petrology, Faculty of Geology, University of Warsaw, Żwirki i Wigury 93, 02-089 Warszawa, Poland; 2 Biological and Chemical Research Centre, Department of Chemistry, University of Warsaw, Żwirki i Wigury 101, 02-093 Warszawa, Poland; 3 Synchrotron SOLEIL, L’Orme des Merisiers, B.P. 48, 91 192 Saint Aubin, Gif-Sur-Yvette Cedex, France

**Keywords:** Solid Earth sciences, Chemistry

## Abstract

As a result of external compression applied to crystals, ions relax, in addition to shortening the bond lengths, by changing their shape and volume. Modern mineralogy is founded on spherical atoms, i.e., the close packing of spheres, ionic or atomic radii, and Pauling and Goldschmidt rules. More advanced, quantum crystallography has led to detailed quantitative studies of electron density in minerals. Here we innovatively apply it to high-pressure studies up to 4.2 GPa of the mineral hsianghualite. With external pressure, electron density redistributes inside ions and among them. For most ions, their volume decreases; however, for silicon volume increases. With growing pressure, we observed the higher contraction of cations in bonding directions, but a slighter expansion towards nonbonding directions. It is possible to trace the spatial redistribution of the electron density in ions even at the level of hundredths parts of an electron per cubic angstrom. This opens a new perspective to experimentally characterise mineral processes in the Earth’s mantle. The use of diamond anvil cells with quantum crystallography offers more than interatomic distances and elastic properties of minerals. Interactions, energetic features, a branch so far reserved only to the first principle DFT calculations at ultra-high-pressures, become available experimentally.

## Introduction

From the very beginning, X-ray diffraction analysis and, in particular, structural mineralogy were based on a simple assumption. The electron density in crystals is represented by spherical ions/atoms which do not exchange electron density. Then, different atomic (ionic) radii could be derived from interatomic distances. This model of electron density is referred to as the Independent Atom Model (IAM)^1^. IAM is the twentieth century application of Kepler’s idea of “spherical hard cannon balls”^2^. It does not allow atoms to exchange electron density nor model its deformation due to chemical bonding and local interactions. However, IAM was, and still is, the most successful model of electron density used in science. Approximately, 99.7% of all 1.5 mln known crystal structures were solved and refined with it. Furthermore, in high-pressure studies of bulk modulus and equation of state, correct information of only unit cell parameters is adequate.

**Figure 1:**
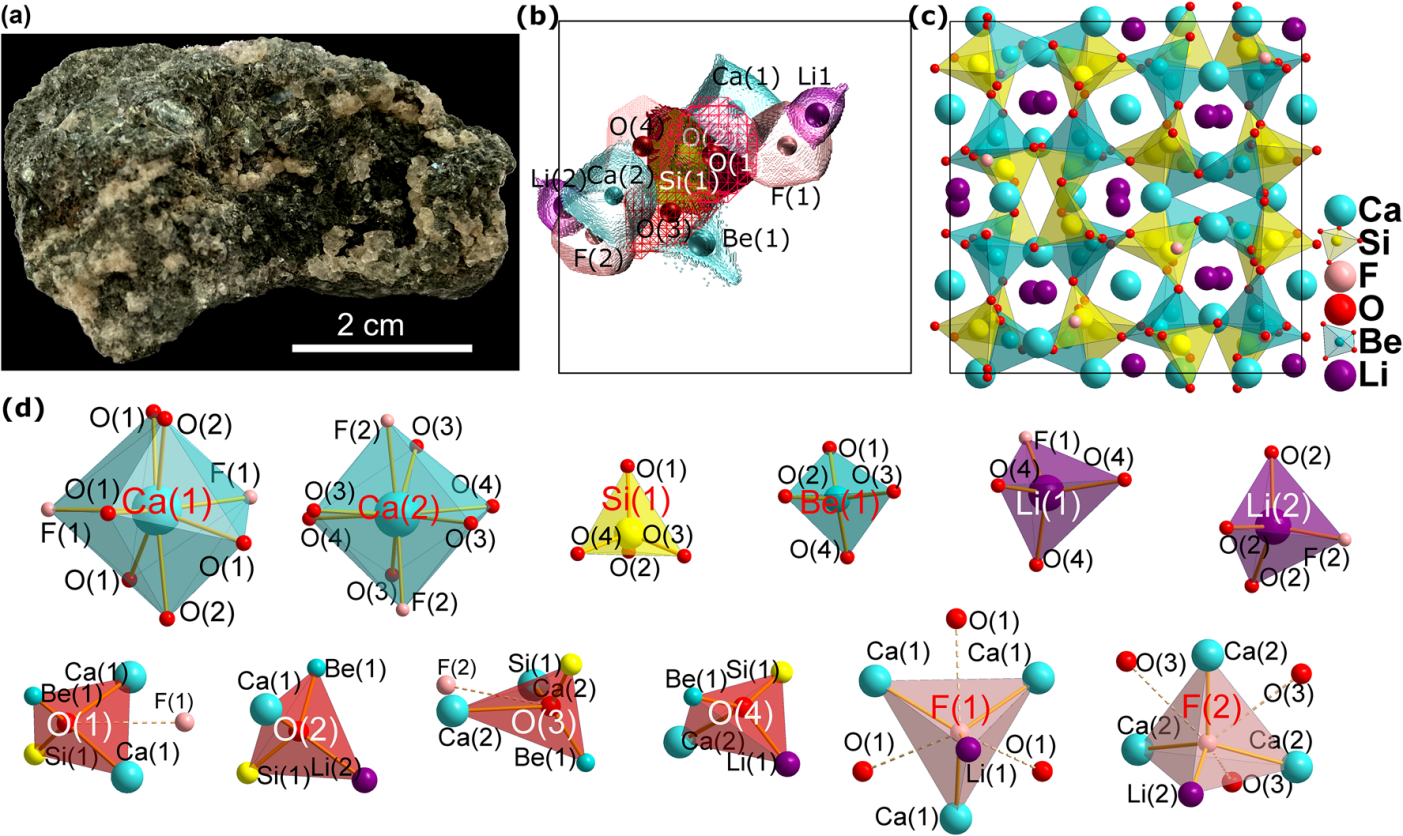
The host rock containing transparent hsianghualite crystals. The mineral forms small, several millimetre in diameter, rounded crystals, sometimes with poorly developed dodecahedral faces or granular and compact aggregates (**a**). The representation of ions (so called ionic basins), bordered by the zero-flux surfaces of electron density (**b**), the polyhedral representation of the crystal structure of **His** (**c**) and fundamental polyhedra present in the **His** structure (**d**).

The common model for interpreting structures is by spherical ions held together by ionic bonds. Similar charges stay apart as far as possible in the mineral’s ground state and are surrounded by ions of opposite charge sign (Pauling’s first rule). The resulting coordination polyhedra define coordination numbers as illustrated for hsianghulaite (**His**) in Fig. 1. A logical consequence is to introduce a size of ion defined by radius and formal charge.

According to Pauling’s second rule^3^, the ionic bond strength is related to an ion’s charge divided by its coordination number. The idea was followed *inter alias* by Brown^4^, whose method is now widely used in the crystallography of minerals. It relays on correlation of bond distance to bond valence parameters retrieved from interpolation from large datasets of crystal structures. The alternative approach presented in our work is universal and does not require any assumptions or interpolated parameters. This is essential for heteroanionic systems as in **His**.

When IAM was introduced, the errors associated with this model were overwhelmed by far larger hardware errors. In the past century and, particularly, in the last few years, there has been significant progress in the design and production of X-ray diffraction devices. Brighter X-ray sources, detectors based on hybrid photon counting technology, and very accurate goniometers are available for small laboratories and large-scale facilities, like synchrotrons or Free Electron Lasers. Nowadays, data are very precise, but still are refined using a more than 100-year-old theoretical formalism, IAM utilising Slater-type 1 s-orbital functions. The directionality of orbitals and chemical bonds, evident from quantum calculations is neglected. IAM refinement of high-quality diffraction data usually leads to the localised residual electron density maxima in the final difference Fourier map. The peaks are observed in the position of lone electron pairs or centred in the middle of the chemical bonds, showing the imperfectness of the model. The close packing of spheres, atomic/ionic radii, Pauling’s rules^3,5^, Goldschmidt’s rules^6^, X-ray/neutron/electron diffraction, and IAM are the cornerstones of modern mineralogy and crystallography. They play a significant role at the level of the geometric structure, as an arrangement of hard spheres. However, they are not useful at the deeper subatomic level of electronic structure of crystals, which we will demonstrate in this work, based on an experimental X-ray diffraction data. We compare our findings with the simultaneous determination of electron density distribution by first principle DFT calculations.

New aspherical approach to the refinement of electron density distribution from X-ray data is a technique within the dynamically developing field of Quantum Crystallography (QC). It opens the opportunity for experimental studies at a level of detail reserved so far to theoretical, first-principle calculations only. We demonstrate how individual ions of zeolite, **His** react to pressure up to 4.2 GPa. We achieve this combining quantum crystallography with high-pressure investigations in diamond anvil cell (DAC).

**Hsianghualite,** Li_2_Ca_3_Be_2_Si_3_O_12_F_2_, (Fig. 1a), belongs to the rarest minerals of the zeolite group^7^. It was described in Chinese by Huang et al.^8^ and more widely known after notes by Fleischer, published in American Mineralogist in 1959^9^ and 1961^10^. The specimen of hsianghualite (Fig. 1a) origins from the Xianghualing Mine (Hsianghualing Mine), Linwu Co., Chenzhou, Hunan, China. The Chinese name of the mine and the mineral means fragrant flower. Until now the mineral was known only from this mine and the nearby Xianghuapu Mine (Maiwan Mine). Hsianghualite occurs in phlogopite veins cutting skarn and thermally metamorphosed limestones in contact with granite intrusions. The mineral forms small, several millimetres in diameter, rounded crystals, sometimes with poorly developed dodecahedral faces or granular and compact aggregates (Fig. 1a). They are colourless, white or pale cream-coloured and reveal a vitreous luster. The crystal structure of hsuanghualite was first determined by Chinese crystallographers in 1973^11^ and later by Rastsvetaeva^12^. The details of the data collection and electron density refinement for **His** can be found in the Supporting Materials (Table S1). **His** was selected for its exceptional crystal quality and very high symmetry, *I*2_1_3. Despite regular system, the asymmetric unit consists of considerably high number of 12 atoms. There are four independent Oxygen atoms, two Ca, F and Li, and one Si and Be. Multiple elements of the same type, allow for comparison of the influence of high-pressure. We believe that these parameters may determine **His** as an ideal candidate for high-pressure investigations at distinct atomic level.

### High-pressure investigations combined with charge density analysis

Except for our feasibility study of electron density in grossular under pressure^13^, there were few attempts to study pure elements^14^ or inorganic materials using the maximum entropy method^15^. There was also a successful study of a molecular organic crystal syn-1,6:8,13-biscarbonyl[14]annulene^16^. Additionally, challenges arising with charge density analysis in crystals at high-pressure have been discussed by Casati and co-workers^17^. None of these reports addressed questions such as: What are the shapes of ions from the electronic point of view?; How much do they depart from the Kepler cannonballs *, i*.e. from the IAM; How much does the electron density change when external stimuli such as pressure or temperature are applied?; Are the ionic radii useful (or even necessary) in their present form?, and—the most important—what fine changes occur in the electron densities of ions under external stimuli. These are topics addressed in this work.

### Importance of high-pressure experimental charge density studies in mineralogy

Applying this formalism gives more accurate and more precise geometrical parameters, compared to IAM refinements, for the studied minerals.

As a consequence, one can accurately describe the interatomic distances, and trace the changes by pressure in the atomic volumes just as in unit cell volumes. These latter variations define the isothermal compressibility, $\kappa_{T}$, or more commonly known as the bulk modulus, $\frac{1}{{\kappa_{T} }}$, of the overall mineral but also of single ions within it. Combining diamond anvil cells with short synchrotron X-ray wavelengths, gives complete, excellent quality diffraction intensities. This is a tool with a potential to retrieve experimental electron densities even at hundreds of GPa pressures, (*i.e.* up to the pressures present in the Earth’s inner core). Such pressures are achieved with DACs of slightly different design and diamond cut. Multiple crystals in optimal mutual orientation in the pressure chamber would be required to achieve 100% completeness in these DACs. We could get insight into the mechanisms of how minerals transform, the nature of phase transitions, the energy of different interatomic interactions, the elastic and other tensor properties of minerals. Tracing the route of phase transformation brings details of plastic deformations at the level of electron density. These results may be adopted to serve in describing large scale geodynamic processes such as, for example, mantle convection or plate tectonics.

## Results and discussion

### Crystal structure

Hsianghualite crystallizes in the regular *I*2_1_3 space group (see Fig. 1). Figure 1b illustrates the ions populating the independent part of the unit cell and their shape defined by so called ionic basins. The boundary is defined by electron density cut-off at 0.001atomic units (1a.u. = e∙bohr^−3^ = 67.49 e∙Å^−3^). Whereas, part (b) and (c) of that figure show the more traditional spherical and polyhedral representations of the contents of the unit cell. The crystal lattice of **His** consists of alternating, ordered, corner sharing SiO_4_ and BeO_4_ tetrahedra (*T*O_4_). The four membered rings of *T*O_4_ are connected into a framework consisting of 6-, 8- and 12-membered rings. Li and F ions are arranged alternately along {111} and fill cavities inside a column of 6-membered *T*O_4_ rings. The LiO_3_F tetrahedra are linked to the framework by a shared O ions. Ca cations fill large cavities along {100}, inside columns, formed by 4-membered *T*O_4_ rings.

The berylo-silicate framework reoriented slightly with pressure, from 1.1 to 4.2 GPa. The O–Si–O and O–Be–O angles within *T*O_4_ tetrahedra remained nearly unchanged with average shift of 0.07° and maximum shift of 0.2° (Table S6). Meanwhile, the angles between corner sharing tetrahedra changed notably. In two groups of Be-O-Si angles, of ca. 121° and 138°, they decreased by 1.2° and by 0.7°, respectively (Table S7).

Details how to obtain an accurate model of electron density (ED) and topological analysis of ED distribution, are described in the Methods and Supporting Materials (see in “Introduction” and “Results and discussion” sections).

### Ionic representation

**Figure 2:**
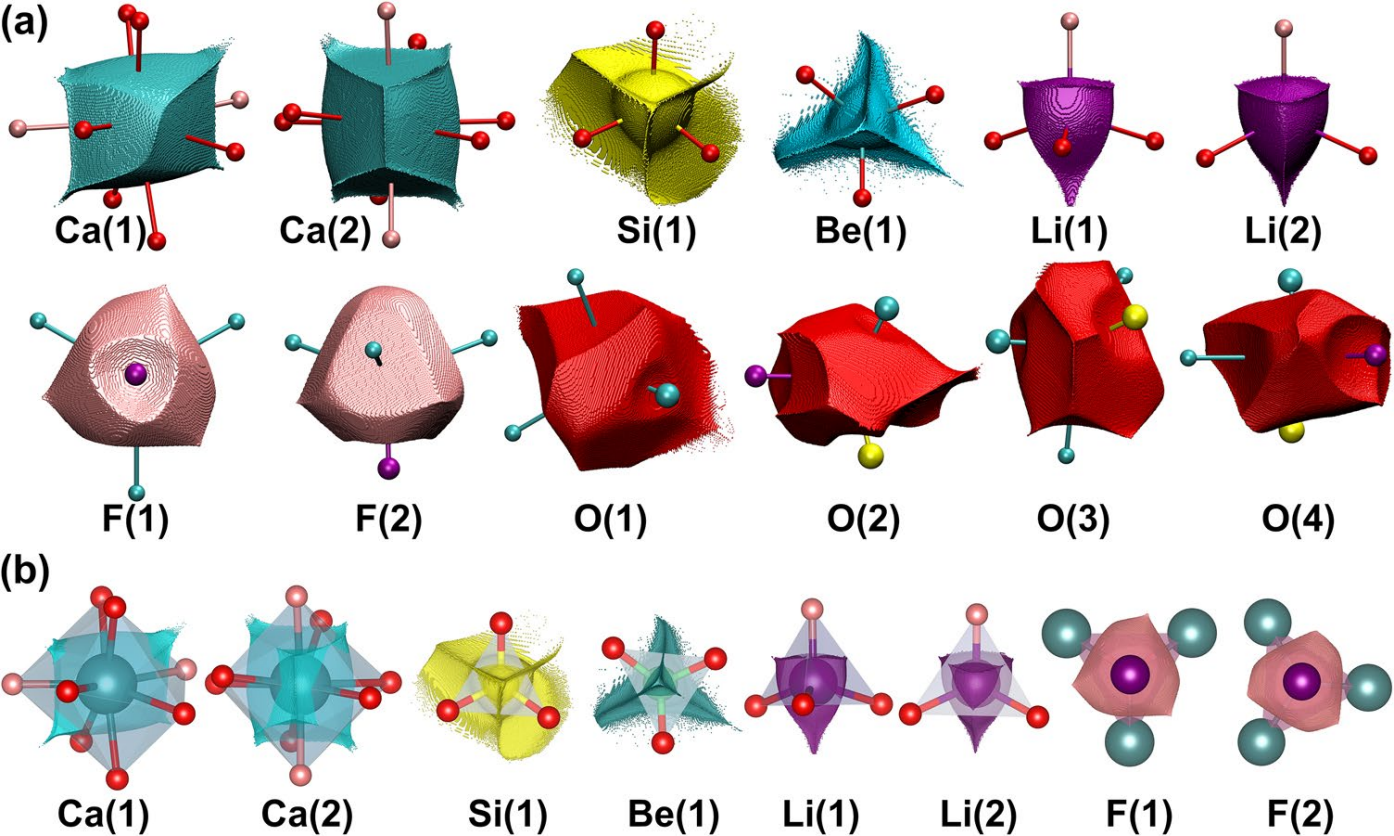
Atomic basins of particular ions present in the **His** structure at 1.9 GPa (**a**) and an overlay of the particular coordination polyhedra and corresponding atomic basins (**b**).

Ions defined as ionic basins play a similar role at the level of electron density as polyhedra at the structural level. These are 3D boundaries of electron density associated with particular ions. For **His**, they are illustrated in Fig. 2a. These shapes reflect the interactions of neighbouring ions, displaying how the valence electron density in the space around them adopts. The coordinating ions influence the shape of central ionic basin (Fig. 2a). 3D video visualisations of all ionic basins are included in the Supporting Materials.

The main difference in polyhedral and ionic basin representations is that the first includes ligands surrounding central ion (Fig. 2b). The bond valence sum^4^ for a given ion corresponds to the integrated charge in the ionic basin. Advantage of the latter is the independence of any empirical constants such as R_*ij*_ or b. Polyhedra are complex figures as they contain some fragments of electron density associated with the central ions and small fragments of electron densities of the corner ions. Ionic basins contain only the electron density associated with central ions. The aspherical shape of an ionic basin is associated with its coordination. The overlap of the polyhedra in **His** and the corresponding ionic basins is illustrated in Fig. 2b.

### Ionic basins under pressure

**Figure 3:**
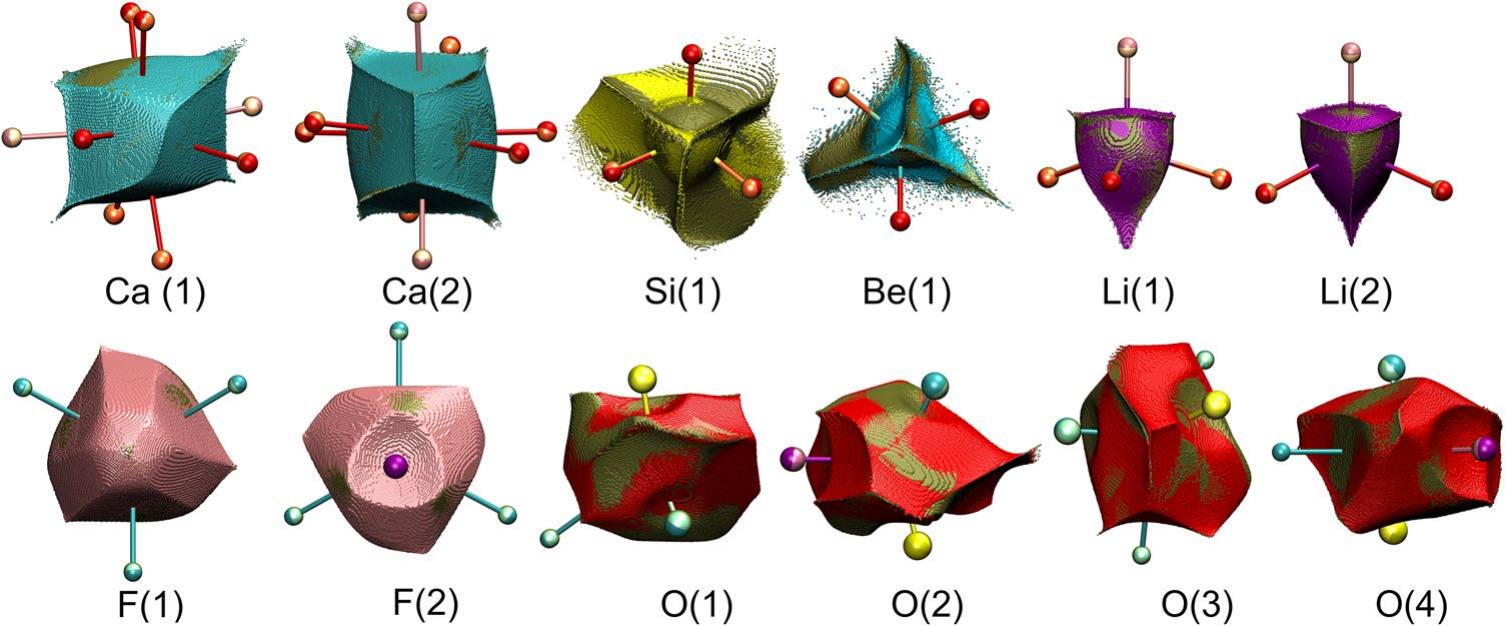
Projections of ionic basins at 4.2GPa (in green) onto ionic basins of the same ions at 1.9 GPa (various colours). Green spots on top indicate the expansion of this fragment of the ion under pressure.

When external stimuli are applied (*e.g.,* pressure or temperature) the interactions among ions in minerals change. Ionic basins also change, slightly yet detectably. One of the ways to demonstrate ionic deformation by pressure (here from 1.9 to 4.2 GPa) is proposed in Fig. 3. Ionic basins of ions at lower pressure are overlaid by their ionic basins under higher pressure coloured in green. Fragments where green colour is on the top indicate expansion due to pressure, i.e. relaxation of electron density. In the remaining places the electron density within an ion is compressed. When pressure is applied, the electron density attempts to compensate this effect by expanding in the anti-gradient directions, i.e. towards nonbonding-edges of ionic basins. A complete set of overlays (1.1 GPa, 1.9 GPa, 4.2 GPa) is given in the Supporting Materials (pdf file) and as 3D rotating views (in power point presentation).

### Changes of electron density inside ionic basins

**Figure 4:**
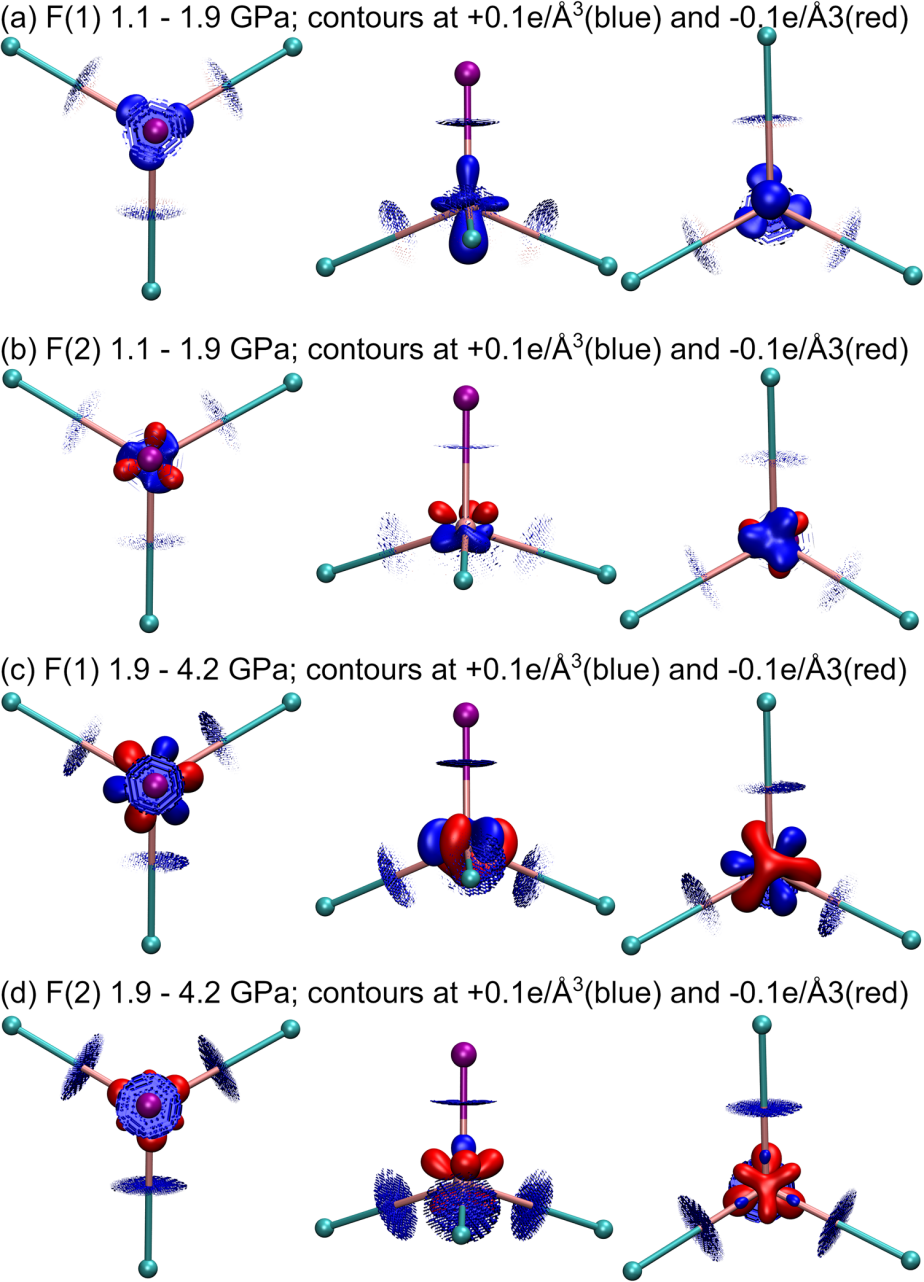
Differences in total electron densities ρ at F anions illustrated at the ± 0.1e/A^3^ isovalues: the total electron density at a higher pressure ρ(P in GPa) is subtracted from the total electron density at lower pressure ρ(1.1)- ρ(1.9) for F(1) (**a**), ρ(1.1)- ρ(1.9) for F(2) (**b**); ρ(1.9GPa)- ρ(4.2GPa) for F(1) (**c**); ρ(1.9GPa)- ρ(4.2GPa) for F(2) (**d**). Isovalues at + 0.1e/Å^3^(blue) and − 0.1e/Å^3^(red). Colours of neighbouring ions: cyan for calcium and violet for lithium.

Another way of tracing changes, with insight closer to nuclei are differential density maps. The electron density values are subtracted at the corresponding points in the space of overlaid ions. The value of electron density at every point, belonging to a given ion at a higher pressure, is subtracted from the corresponding value determined at lower pressure. The grids of electron density were calculated with 0.02 Å intervals. The following abbreviation fashion will be used Δ_1_^F1^_,_ means the difference electron density of 1.1–1.9 GPa for F(1) ion; Δ_2_^F1^ is difference electron density of 1.9–4.2GPa for F(1). Negative values (red) in differential maps indicate places where electron density increases with pressure. Contrary, positive values (blue) indicate an electron density depletion at elevated compression. Defining either contour intervals in 2D maps or isosurface level in 3D view enables quantitative measure of observed changes, *e.g*., for F anions with ± 0.1e/Å^3^ isosurfaces in Fig. 4. This is a direct, quantitative observation how electron density relocates at the 1.9GPa pressure within the F(1) ion.

Tuning the contour steps or isosurface level allows to estimate the size of the electron density changes in e∙Å^−3^. With the isosurface level of 0.1 e⋅Å^3^ two symmetrically non-equivalent F(1) and F(2) ions, despite identical site symmetry show slightly different features (Fig. 4a, d). However, the overall redistribution scheme at the F ionic boundary is similar. The negative values start to appear from: Δ_1_^F1^ =  − 0.15eÅ^−3^, Δ_1_^F2^ =  − 0.11eÅ^−3^, Δ_2_^F1^ =  − 0.05eÅ^−3^, Δ_2_^F2^ =  − 0.04eÅ^−3^. These are located towards nonbonding directions, between Li and Ca that are in fluorides first coordination spheres. The latter set of values shows the level and direction of ionic expansion due to pressure. Contrary, the positive values of differential electron density map near the ionic boundary show the contraction areas of the compressed F ion. These were observed around F-Ca and F-Li bonds, Δ_1_^F1^ = 0.18eÅ^−3^ towards Li and 0.16 eÅ^−3^ towards Ca. Δ_1_^F2^ = 0.17eÅ^−3^, towards Ca and Li. Δ_2_^F1^ = 0.17eÅ^−3^, and 0.16 eÅ^−3^; Δ_2_^F2^ = 0.2eÅ^−3^ and 0.18 eÅ^−3^, towards Li and Ca, respectively.

A complete set of differential maps at ± 0.1 and ± 0.05 eÅ^−3^ is given in the Supporting Materials (pdf file) and as 3D rotating view (in power-point presentation). Around F–Ca bonding directions, further from the nucleus (Fig. 4), the mentioned above positive values (compression) are shifted slightly towards Li, whereas negative values (expansion) are shifted towards empty, nonbonding regions. 

A slight reorientation of berylo-silicate framework with pressure, manifested itself on differential representation of ions. The colours were changing on adjacent faces, red vs blue, showing the direction of atom’s rotation. Notably, Si rotated more than Be. The framework reorientation was demonstrated with a polyhedral representation and with differential density maps of ions in Supplementary power-point presentation.

### Changes of the total ionic charge with pressure

**Figure 5:**
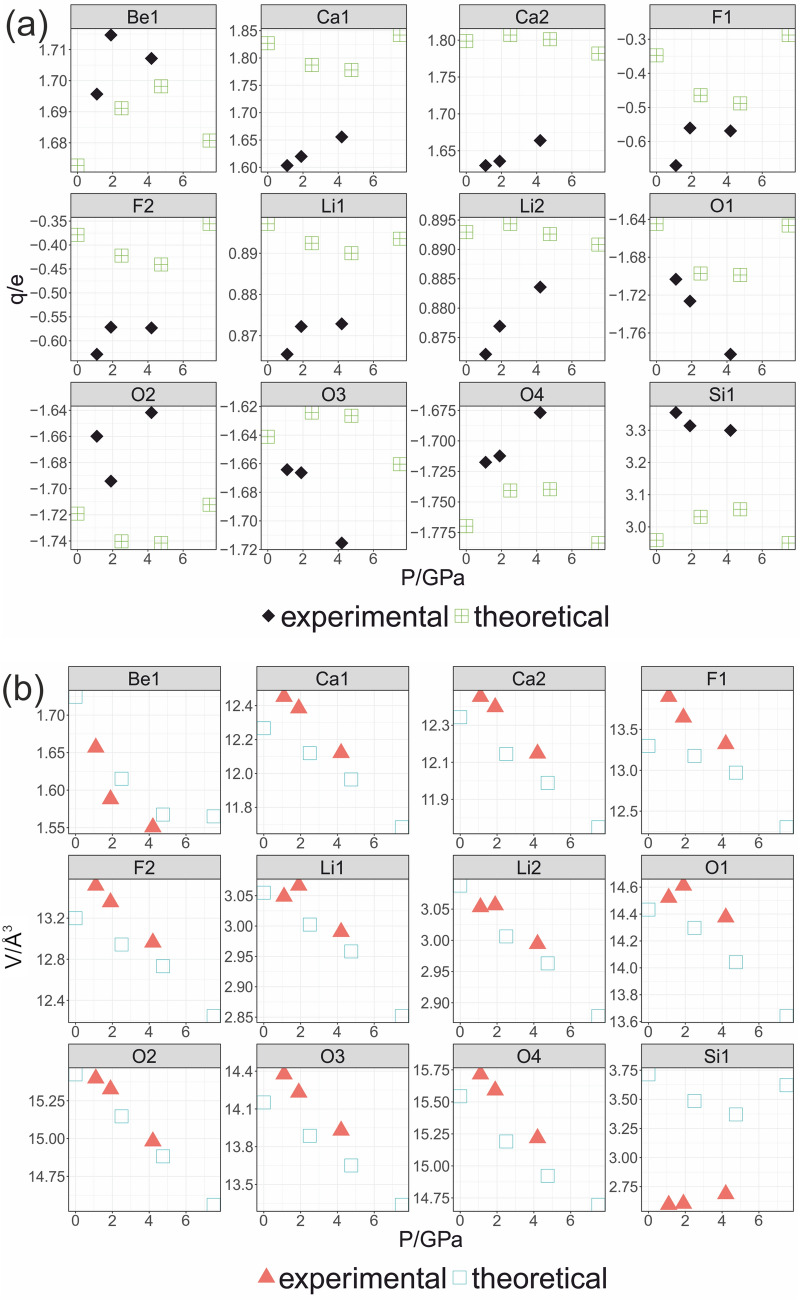
Changes of the ionic charge (**a**) and ionic volume (**b**) for ions in the hsianguhalite crystal structure under pressure.

Integration of the electron density over ionic basins gives the charges incorporated within ions. Electron density redistributes under pressure, changing also the curvature of ionic basins zero flux surfaces. Thus, we observed an interionic charge flow in **His** due to external pressure. Figure 5 presents the charges of ions and their sizes. The results are compared with the first principles calculations.

Neither the experimental nor theoretical total ionic charges take formal values (see Fig. 5a, note individual scales for all ions). The greatest change is observed for F(1) from 1.1 GPa to 1.9 GPa, by increasing + 0.11 e charge. Next, O(1) gains − 0.8e charge. When pressure is increased from 1.1 GPa through 1.9 GPa, to 4.2 GPa, the charge of Ca(1), Ca(2) and O(1) ions change monotonically. For Be(1), Li(1), Li(2) the charges basically do not change, staying in a range of ± 0.02e. For the remaining ions changes are in average ± 0.05e.

The utmost inconsistency with the DFT results is found for Si(1) integrated charges being ca. + 0.3e higher in experiment. The overall trends are mostly nonlinear. Both experimental and theoretical electron densities in ionic basins imply that pressure is a driving force for charge transfer among ions. In theory, with pressures the electronegativity of elements (single atoms) decreases^18^. In a crystal, two competing processes may be considered. On one hand, increasing pressure elevates the electrostatic, ionic interactions. This changes the charge of cations and anions influencing the shape and size of ionic basins. On the other hand, increasing pressure affects the electronegativities of ions, allowing interionic charge transfer, which apparently is nonlinear.

### Changes of ionic volumes under pressure

High-pressure leads to contraction of the unit cell and in most cases decrease in volume of ionic basins (Fig. 5b). An excellent agreement between experiment and theory was found, except for Si(1). In experiment Si expands, increasing its volume with pressure. We defined the degree of compression (*i.e.,* the softness or hardness of ionic basins) as an average of ΔV/ΔP, where ΔV is the change in ionic volume and ΔP, pressure difference. With this definition the least compressible ion in **His** is Si(1), followed by (O1), both revealing expansion with pressure (O(1) from 1.1 to 1.9 GPa, Si(1) in all ranges). This phenomenon is directly related to interionic electron density redistribution, outweighing pressure compression.

More details on ionic volumes, bond critical parameters and ADP values under pressure are described in the Supporting Materials.

## Conclusions

Ionic basin representation allows the capture of detailed changes in interacting ions with increasing pressure. In contrast to polyhedral representation, individual ions are examined with distinguished bonding or nonbonding fragments. Their 3D shapes characterise intermolecular interactions and are very sensitive even to small changes in external stimuli such as pressure. The shape of ions, defined by ionic basins is very anisotropic, non-spherical. Thus, cornerstones of modern crystallography and mineralogy, such as ionic/atomic radii, polyhedra, Pauling’s and Goldschmidt rules seem less practical at the level of quantitative studies of electron density in minerals.

The measured charge of ions in crystals differed from the formal unit values. We observed a redistribution of charge among ions, mostly F and O anions, due to applied external pressure. Redistribution within  a single ion was also possible to quantify. Even though the total volume of a given ion decreased with pressure, there are fragments of this ion that actually swelled up. It was observed mostly in anti-gradient direction of electron density, where no bonds were formed, *i.e.* towards least electron density accumulation. Most of ions showed the largest compression along their bonds. Exceptionally, Si forming bonds with the highest covalent contribution, expanded towards coordinated O ions with external pressure.

Presented approach opens new perspectives for experimental and theoretical studies on subatomic level of electron density. For all the processes taking place in the Earth’s mantle, such as the ability of mantle rocks to flow during decompression and its relevance to plate movements, phase transitions of minerals, formation of new minerals at pressure, and variable temperatures at subatomic levels of quantitative changes in the electron density of ions in minerals.

## Methods

### Combined HP X-ray diffraction data collection

High-pressure X-ray diffraction experiments on single crystals no. 1 and 2 of hsianghualite were carried out at the CRISTAL beam-line of the SOLEIL synchrotron at 293 K temperature. The wavelength was determined to have the value of 0.4166 Å. A specially designed, (wider, 120° opening angle), diamond anvil cell, DiacellOne20DAC from Almax EasyLab, was used with a ruby chip as pressure indicator and 4:1 methanol-ethanol mixture as pressure medium. The sample was placed in a DAC equipped with 0.5 mm culet diamonds and fitted with a steel gasket of initial thickness 0.2 mm and a 0.3 mm gasket hole. The pressure was set to 1.1 GPa (HP1) and to 1.9 GPa (HP2). A detailed description of the design of DiacellOne20DAC, synchrotron wavelength calibration and sample centring procedure is presented in our previous work^13^.

The second piece of single crystal was mounted into a triangle DAC equipped with 0.7 mm culet diamonds with narrower, 70° opening angle and pressure was set to 4.2 GPa (HP4). A tungsten gasket of 0.24 mm initial thickness and 0.3 mm hole diameter was used.

The overall data redundancy to a resolution of 0.5 Å for crystal no 1 at 1.1 and 1.9 GPa was: redundancy 26 with 99.9% completness and 14.2 with 99.8%, respectively. For crystal no. 2 at 4.2 GPa it was 11 with 82.4% completeness.

The images from all X-ray diffraction experiments were processed with the CrysAlis PRO software suite (Rigaku Oxford Diffraction, 2020). Reflection intensities were corrected for Lorentz, polarisation, and absorption effects and converted to structure factors using CrysalisPro software. Data were treated using the dedicated HP routines present in CrysalisPro and described in detail previously^13^.

### Experimental determination and modelling of the ED distribution at 1.1, 1.9 and 4.2 GPa pressure

The basis axes *X*, *Y*, *Z* for all atoms and dependent multipoles were chosen according to the XD2020 documentation, adequately to the occupied crystallographic sites. This choice guaranteed that the refined multipolar parameters maintain the point symmetries of the individual ions and the overall symmetry of the crystal. The non-spherical multipole populations of Ca, Be and Li were not refined due to the spherical character of the valence shell (s orbitals) of the ions. The refinement strategy for the multipole model was as follows: atomic coordinates (*xyz*), anisotropic displacement parameters (*U*_*ij*_) were refined together with the scale factor (*s*) using the high-order X-ray diffraction data of sinθ/λ > 0.7 Å^−1^. The terms *xyz*, *U*_*ij*_ and *s* were fixed after high-order refinement. Then the procedure was performed in a stepwise manner, adding new parameters in the following order: scale factor (*s*) and valence populations (*M*), dipoles (*D*), quadrupoles (*Q*), octupoles (*O*), hexadecapoles (*H*). For F ions, due to symmetry constraints only the following multipoles were allowed for the refinement *M1*, D0, Q0, O0, O3 + , O3 + , O3-, H0, H3 + , H3-. Next, *s* was refined with spherical κ parameters for the Si, F and O ions. In the final stage the *s*, *xyz* and *U*_*ij*_ parameters were refined. Each refinement cycle was considered fully converged at the point at which the maximum shift/standard uncertainty (s.u.) ratio was less than 10^–4^_._

### Charge density analysis

The charge density distribution and related properties were analysed with the aid of Bader’s QTAIMC theory^19^. More information about this theory can be found in the Supporting Materials. Calculations of BCPs, bond paths, the integrated charges and volumes of ionic basins were performed in the XD2020 program package^20^. The models of ions were calculated and visualised at the electron density level of 0.001au. Partitioning of the electron density into ionic basins was done using the TOPXD within XD2020 and BADER programs^21^. Charge density cubes were calculated with 0.02 Å grid size.

### Theoretical calculations

CRYSTAL17^22,23^ software was used for DFT calculations. We applied B3LYP^24–26^ exchange–correlation functional corrected for dispersion by Grimme's D3^27^ correction in conjunction with the pob_TZVP_rev2 basis sets^28^. Geometry optimisation of unit cell and atomic positions at given pressures was performed. Then theoretical dynamic structure factors were calculated with CRYSTAL17^22,23^ on energetically converged structures at pressures ranging from 0 to 7 GPa. Next, structure factors were used in a multipolar refinement of the electron density using the XD2020 program package^20^.

### Additional information

Access codes: The X-ray crystallographic coordinates for structures reported in this study have been deposited at CSD under deposition numbers: 2232592-2232594. These data can be obtained free of charge from CSD via www.ccdc.cam.ac.uk/data_request/cif.

## Supplementary Information

Supplementary Information.

## Data Availability

The datasets generated and/or analysed during the current study are available in the RepOD repository https://doi.org/10.18150/CKBWZY
