# Supplementary material for: Charge density redistribution with pressure in a zeolite framework
Source: Sci Rep. 2023 Jan 28;13:1609. doi: 10.1038/s41598-023-28350-4 (PMC9884199; doi:10.1038/s41598-023-28350-4)
Supplement: Supplementary file 1 — Supplementary Information. [file 41598_2023_28350_MOESM1_ESM.pdf]

Supporting materials

Charge Density Redistribution with Pressure in a Zeolite Framework

Marcin Stachowicz[1]*, Roman Gajda[2], Agnieszka Huć[2] Jan Parafiniuk[1], Anna Makal[2], Szymon Sutuła[2], Pierre Fertey[3], Krzysztof Woźniak[2]*

[1]Department of Geochemistry, Mineralogy and Petrology, Faculty of Geology, University of Warsaw, Żwirki i Wigury 93, Warszawa 02-089, Poland,
[2]Biological and Chemical Research Centre, Department of Chemistry, University of Warsaw, Żwirki i Wigury 101, Warszawa 02-093, Poland,
[3]Synchrotron SOLEIL, L'Orme des Merisiers - Saint Aubin, B.P. 48, Gif-sur-Yvette Cedex 91 192, France

Email: marcin.stachowicz@uw.edu.pl, kwozniak@chem.uw.edu.pl

Contents

1. Experimental quantitative charge density studies – main ideas.
2. Topological analysis of electron density.
Table S1. The crystal information and details of X-ray diffraction data collection and refinement for hsianghualite.
Figure S1. The equivalent isotropic values (*U*eq) of displacement parameters for all atoms plotted vs. *U*eq at 1.1 GPa.

Table S2. Fractional atomic coordinates, equivalent isotropic displacement parameters U_{eq}(Å^2) and Atomic displacement parameters U^{ij}(Å^2).
Table S3. Integrated charges and volumes of Bader ionic basins.
Table S4. List of bond/interatomic distances in hsiaghualite at various pressures.
Table S5. Bond critical point parameters and corresponding electronegativity. rho – electron density in e·Å^{-3} d2rho – laplacian of electron density in e·Å^{-5}. distances, Rij and bonded radii in Å.
Table S6. List of angles O-Si-O angles in SiO4 and O-Be-O angles in BeO4 tetrahedra
Table S7. List of Si-O-Be angles bwtween *T*O4 tetrahedra forming hsianghualite infinite framework.
3. Theoretical calculations
4. Visualisations of differences in the total electron densities of the central ion for two values of pressure applied for single crystal of hsianghualite
5. List of angles in the crystal structure of hsainghualite at 1.1, 1.9 and 4.2 GPa

1. Experimental quantitative charge density studies – main ideas.

It is essential to collect accurate X-ray data to a sufficiently high resolution (high diffraction angle). Aspherical modelling brings up to 32 additional parameters per atom. Independent Atom Model (IAM) refines only 9, *i.e.* positions and atomic displacement parameters (ADPs), related to atomic thermal vibrations. High resolution is necessary to match a sufficient observation/parameter ratio and to properly deconvolute valence electron density from the thermal motions of atoms. High resolution reflections are strongly associated with nuclei positions (i.e. main contribution form the core electron density) and particularly with ADPs. The valence electron density contributes mostly to the low angle diffraction reflections. The data set should be sufficiently complete to avoid systematic effects in the refinement – ideally 100% complete, as for **His** Some very accurate and precise measurements of the intensities of the reflections are needed to minimize systematic effects such as absorption by the sample, extinction and, very importantly here, absorption by the apparatus itself, namely the diamonds and the metal gasket. Furthermore, the contribution of valence electron to the total reflection intensity never exceeds a few percent which reinforces the need for very accurate intensity measurements. Apart from accurate correction of the data, modern area detector technologies offer the possibility of improving the precision of the measurement by repeated collection of the same intensities.

The most common, aspherical quantitative model of experimental charge density, is based on a finite spherical harmonic expansion of the electronic part of the charge distribution about each atomic centre. An atomic expansion is called a pseudoatom. In the formalism of Hansen and Coppens[1,2] the pseudoatom electron density is defined as:

$$\rho(\mathbf{r}) = \rho_c(r) + P_v \kappa^3 \rho_v(\kappa r) + \sum_{l=0}^{l_{max}} \kappa'^3 R_l(\kappa' r) \sum_{m=0}^{l} P_{lm\pm} d_{lm\pm}(\theta,\varphi)$$

where $\rho_c(r)$ and $\rho_v(r)$ are spherical core and valence densities, respectively. The third term contains the sum of the angular functions $d_{lm\pm}(\theta,\phi)$ to take into account the aspherical deformations. The angular functions $d_{lm\pm}(\theta,\phi)$ are real spherical harmonic functions. The coefficients P_v and $P_{lm\pm}$ are populations for the valence and deformation density multipoles, respectively. κ and κ' are scaling parameters introduced to make valence and deformation densities expand or contract. In the Hansen-Coppens formalism, P_v, $P_{lm\pm}$, κ and κ' are refinable parameters together with the atomic coordinates and thermal coefficients. Least-squares refinements are performed against the measured intensities of reflections (*i.e.* F^2(hkl)) obtained by single crystal X-ray diffraction. This requires a data resolution of 0.50 Å and full data completeness. Starting atomic coordinates and anisotropic displacement parameters are taken from the ordinary spherical refinement stage and freely refined. Each atom was assigned a core and spherical-valence scattering factors derived from the Su and Coppens wavefunctions[3]. A single-ζ Slater type radial function multiplied by density-normalized spherical harmonics was used for describing the valence deformation terms. The multipole expansion was truncated at the hexadecapole level for Si, F and O atoms. The radial fit of the valence density was optimized by refinement of the expansion-contraction parameter κ for these atoms. Ca, Li and Be were due to spherical valence s orbitals were refined to Pv, valence population level.

Once an aspherical atomic electron density ρ(r) was defined, then it could be used to obtain aspherical atomic form factors and aspherical structural factors for a given crystal. When hundreds of thousands of reflections (or sometimes even more than one million) were measured and used in the refinement, the previously mentioned parameters such as the populations of electrons, contraction/expansion coefficients could be obtained.

To acquire good charge density data some special requirements have to be fulfilled. These are: very good quality single crystals, high resolution (≥ 0.5Å) data to analyse even subtle changes of the electron density, low temperature measurements (100K or even 10K and lower when possible, although for many mineral crystals this requirement is less important), accurate and precise intensity measurements with errors small enough not to influence the bonding electron density and careful corrections for different technical effects such as absorption, extinction, background and thermal diffuse scattering (TDS), variation of incident beam intensity.

Experimental charge density studies of minerals are still very rare in the crystallographic and mineralogical communities. Up to our knowledge, the following minerals have been studied by applying multipolar refinements: diopside $CaMgSi_2O_6$[4], α-spodumene $LiAlSi_2O_6$[5,6], natrolite $Na_2Al_2Si_3O_{10}\cdot 2H_2O$[7], scolecite $CaAl_2Si_3O_{10}\cdot 3H_2O$[8], phenakite Be_2SiO_4[9,10], coesite SiO_2[11], stishovite SiO_2[12], rutile TiO_2[13,14], azurite $Cu_3(CO_3)_2(OH)_2$[15] and cuprite Cu_2O[16,17], topaz $Al_2[SiO_4]F_2$[18], datolite $Ca[BOH(SiO_4)]$[19], dioptase

$Cu_6[Si_6O_{18}]\cdot 6H_2O$[20], diaspore[21] AlOOH, triphylite[22] $LiFe(PO_4)$, hematite[23] α-Fe_2O_3, nitratine[24] $NaNO_3$, mesolite[25] $Na_2Ca_2[Al_2Si_3O_{10}]3\cdot 8H_2O$, α-Al_2O_3 corundum[26], periclase[27] MgO, magnesiowüstite[27] (Mg,Fe)O, forsterite[28] Mg_2SiO_4, calcium silicide[29] CaSi, fluorite[30] CaF_2, niter[31] KNO^3, nitratine[31] $NaNO_3$ and grossular[32] $Ca_3Al_2(SiO_4)_3$.

Notably, there could also be a few more charge density studies of minerals accomplished in Russia (particularly, in the very active Tsirelson's group in Moscow) which have been published in Russian journals which are not covered by common literature databases. Thus, these papers are difficult to identify and these data may not be on our list.

2. Topological analysis of electron density.

Once the quantitative electron density distribution in a mineral is established, different methods of electron density partitioning can be used to analyze the properties of the studied systems. These include such methods as the stockholder[33] pseudoatom partitioning, or - the most popular - Atoms-In-Molecules theory[34] (AIM) proposed by R. Bader. AIM theory[35,36] offers a self-consistent way of partitioning any molecular or crystalline system into its atomic fragments, deduced from the first principles of Quantum Mechanics and Schwinger's principle of stationary action[37]. In AIM theory, a many electron system is separated into subsystems (ionic basins) by *zero-flux surfaces* (ZFSs). Any point on this surface satisfies the equation $\mathbf{n}\bullet\nabla\rho(\mathbf{r}) = 0$, where $\nabla\rho(\mathbf{r})$ is the gradient vector field of the electron density, **r** is a point on the zero-flux surface that separates two fragments, and **n** is the vector normal to the surface at that point. Further analysis of the gradient vector field of electron density results in localization of the extremes of the electron density by finding *critical points* (CP) at which $\nabla\rho(\mathbf{r}_{CP}) = 0$. Particularly useful are bond critical points – the weakest points in bonds which define their properties. Integrating properties over ionic basins is one of the cornerstones of AIM theory because it yields valuable information such as the integrated charges, the volumes of atoms/ions, their energies, and electronic populations as well as higher multiple moments[35], polarizabilities[38], *etc*.

We have verified these using experimental data[39]. The first four criteria concern bond critical point (BCP) properties: the existence of BCPs, charge density and Laplacian at BCPs and mutual penetration of interacting atoms. Four additional necessary criteria are based on the integrated properties of atoms and concern: loss of charge, destabilisation of atom, decrease of the dipolar depolarization and atomic volumes of the interacting atoms in relation to the non-interacting ones. All the above parameters of electron density are quantitative in nature and are useful descriptors to study electronic effects, intra and intermolecular interactions in minerals.

According to Pauling's second rule[40], the ionic bond strength is related to an ion's charge divided by its coordination number. The idea was followed *inter alios* by Brown[41] with bond valence model, whose method is now widely used in the crystallography of minerals. The corresponding to strength, bond valence (S_{ij}) is quantitatively correlated to the bond distances of coordinated atoms *i* and *j*. $S_{ij} = \exp((R_{ij}-d_{ij})/b)$, where d_{ij} is the distance between atoms, R_{ij} is an average, empirically determined distance, b, usually equal to 0.37 is a constant determined by interpolation from a large dataset of crystal structures. Several sets of bond-valence parameters (R_{ij}, b) are often available for unique ion pairs, and there has been little comparison between different sets of parameters available to determine which is the most suitable for a given ion pair. The alternative approach presented in our work is universal and does not require any assumptions or interpolated parameters. This is essential for heteroanionic systems as in **His**.

The estimation of integration errors. The sum of integrated charges within ionic basins per formula unit $Ca_3Li_2(Be_3Si_3O_{12})F_2$ is 0.21e, 0.18e and 0.16e for 1.1, 1.9 and 4.2 GPa, respectively (for DFT values are within 0.03-0.06e). Ideally values should equal 0. To estimate errors associated with the method we relate above values to 112 valence electrons per formula unit, for which populations were refined. We get errors of 0.19%, 0.16% and 0.14% respectively for all three experiments. The sum of ionic basins volumes differ from unit cell size by 0.07%, 0.04%, and 0.08%, respectively.

Changes of ionic volumes under pressure. High-pressure leads to contraction of the unit cell and in most cases decrease in volume of ionic basins (Fig 5b). An excellent agreement between experiment and theory was found, except for Si(1). In experiment Si shows negative compressibility, increasing its volume with pressure. We defined the degree of compression (*i.e.* the softness or hardness of ionic basins) as an average of $\Delta V/\Delta P$, where ΔV is the change in atomic volume and ΔP, pressure difference. With this definition the hardest atom in **His** is Si(1), followed by (O1), both revealing expansion with pressure (O(1) from 1.1 GPa to 1.9 GPa, Si(1) in all ranges). This phenomenon is directly related to interatomic electron density redistribution. Both ionic basins gained electron density when expanded. The effect outweighed pressure

compression. All the remaining anions are soft, the softest being fluorides with an average -0.19 Å^3/GPa, -0.16 Å^3/GPa for F(1) and F(2), respectively. The smallest change in either direction is shown by Li ions. Be(1), despite a relatively small average compression rate -0.04 Å^3/GPa, decreased the most, from 1.1 GPa to 4.2 GPa by 6.4% overtaking F(1), F(2) with 4.2% and 4.1% compression, respectively. In absolute values anions compress to a greater extent than cations. With the increasing size of an ion it becomes it is more susceptible to compression.

Table S1. The crystal information and details of X-ray diffraction data collection and refinement for hsianghualite.

**Crystal data**	**1.1 GPa**	**1.9 GPa**	**4.2 GPa**
4.2GPaChemical formula	$Ca_3Li_2Be_3(SiO_4)_3F_2$	$Ca_3Li_2Be_3(SiO_4)_3F_2$	$Ca_3Li_2Be_3(SiO_4)_3F_2$
M_r	475.42	475.42	475.42
Crystal system, space group	Cubic, $I2_13$	Cubic, $I2_13$	Cubic, $I2_13$
Temperature (K)	293(1)	293(1)	293(1)
a (Å)	12.82743 (2)	12.80182 (2)	12.71733 (4)
V (Å^3)	2110.66 (1)	2098.04 (1)	2056.78 (2)
Z	8	8	8
Radiation type	synchrotron, λ = 0.41625 Å	synchrotron, λ = 0.41625 Å	synchrotron, λ = 0.41625 Å
μ (mm^{-1})	0.45	0.45	0.45
Crystal size (mm)	0.08 × 0.08 × 0.08	0.08 × 0.08 × 0.08	0.04 × 0.04 × 0.04
**Data collection**			
Diffractometer	Cristal Beamline, Soleil	Cristal Beamline, Soleil	Cristal Beamline, Soleil
Absorption correction	Multi-scan, SCALE3 ABSPACK	Multi-scan, SCALE3 ABSPACK	Multi-scan, SCALE3 ABSPACK
T_{min}, T_{max}	0.739, 1.000	0.853, 1.000	0.874, 1.000
No. of measured, independent and observed [$I > 2\sigma(I)$] reflections	49140, 4915, 4863	54460, 7257, 6926	25118, 2998, 2952
R_{int}	0.056	0.046	0.041
$(\sin \theta/\lambda)_{max}$ (Å^{-1})	1.190	1.387	1.111
**Refinement IAM**			
$R[F^2 > 2\sigma(F^2)]$, $wR(F^2)$, S	0.020, 0.050, 1.11	0.020, 0.048, 1.07	0.017, 0.046, 1.08
No. of reflections	4915	7257	2998
No. of parameters	77	77	77
$\Delta\rho_{max}$, $\Delta\rho_{min}$ (e Å^{-3})	0.47, -0.46	0.47, -0.33	0.43, −0.39
Absolute structure parameter	0.01 (2)	0.042(17)	0.02 (3)
**Refinement AAM**			
$R[F^2 > 2\sigma(F^2)]$, $wR(F^2)$, S	0.014, 0.034, 1.128	0.011, 0.028, 0.97	0.014, 0.034, 1.13
No. of reflections	2965 missing (14,14,-14) (15,15,-14)	2946 missing (14,14,-14), (14,15,-13), (15,15,-14), (16,14,-14), (15,16,-13), (16,15-13)	2393
No. of parameters	294	294	294
$\Delta\rho_{max}$, $\Delta\rho_{min}$ (e Å^{-3}) (denser sampling)	0.389, -0.300	0.34, -0.267	0.351. -0.391

Table S2. Fractional atomic coordinates, equivalent isotropic displacement parameters U_{eq}(Å^2) and Atomic displacement parameters U^{ij}(Å^2).

P/ [GPa]	Atom/ion	*x/a*	*y/b*	*z/c*	U_{eq}	U^{11}	U^{22}	U^{33}	U^{12}	U^{13}	U^{23}
1.1	**Ca(1)**	0.5	0.75	0.586428 (9)	0.007	0.00723 (4)	0.00645 (4)	0.00731 (4)	−0.00173 (3)	0	0
1.9		0.5	0.75	0.586461 (7)	0.007	0.00682 (3)	0.00608 (3)	0.00688 (3)	−0.00160 (2)	0	0
4.2		0.5	0.75	0.586612 (11)	0.006	0.00639 (4)	0.00576 (4)	0.00629 (4)	−0.00140 (4)	0	0
1.1	**Ca(2)**	0.155803 (9)	0.5	0.75	0.007	0.00669 (4)	0.00665 (4)	0.00747 (4)	0	0	0.00145 (3)
1.9		0.155938 (7)	0.5	0.75	0.006	0.00619 (3)	0.00626 (3)	0.00705 (3)	0	0	0.00130 (2)
4.2		0.156166 (11)	0.5	0.75	0.006	0.00585 (4)	0.00587 (4)	0.00659 (4)	0	0	0.00112 (4)
1.1	**Si(1)**	0.344767 (9)	0.579562 (9)	0.623785 (9)	0.004	0.00402 (4)	0.00417 (4)	0.00430 (4)	−0.00006 (3)	−0.00003 (3)	0.00018 (3)
1.9		0.344861 (7)	0.579465 (7)	0.623741 (7)	0.004	0.00380 (3)	0.00392 (3)	0.00414 (3)	−0.00002 (2)	−0.00005 (2)	0.00018 (2)
4.2		0.345090 (11)	0.579232 (11)	0.623655 (11)	0.004	0.00355 (4)	0.00375 (4)	0.00402 (4)	−0.00010 (4)	−0.00003 (4)	0.00018 (4)
1.1	**F(1)**	0.64542 (2)	0.64542 (2)	0.64542 (2)	0.01	0.00993 (7)	0.00993 (7)	0.00993 (7)	−0.00020 (8)	−0.00020 (8)	−0.00020 (8)
1.9		0.645407 (18)	0.645407 (18)	0.645407 (18)	0.009	0.00942 (5)	0.00942 (5)	0.00942 (5)	−0.00017 (6)	−0.00017 (6)	−0.00017 (6)
4.2		0.64548 (3)	0.64548 (3)	0.64548 (3)	0.009	0.00873 (9)	0.00873 (9)	0.00873 (9)	−0.00024 (10)	−0.00024 (10)	−0.00024 (10)
1.1	**F(2)**	0.10462 (2)	0.39538 (2)	0.60462 (2)	0.01	0.00953 (7)	0.00953 (7)	0.00953 (7)	0.00003 (7)	−0.00003 (7)	0.00003 (7)
1.9		0.104720 (18)	0.395280 (18)	0.604720 (18)	0.009	0.00890 (5)	0.00890 (5)	0.00890 (5)	0.00008 (6)	−0.00008 (6)	0.00008 (6)
4.2		0.10477 (3)	0.39523 (3)	0.60477 (3)	0.009	0.00856 (8)	0.00856 (8)	0.00856 (8)	0.00013 (10)	−0.00013 (10)	0.00013 (10)
1.1	**O(1)**	0.43340 (2)	0.61697 (2)	0.70712 (2)	0.006	0.00553 (9)	0.00807 (10)	0.00490 (9)	−0.00070 (8)	−0.00035 (7)	−0.00053 (7)
1.9		0.433823 (18)	0.616813 (19)	0.707007 (17)	0.006	0.00547 (7)	0.00779 (7)	0.00434 (7)	−0.00072 (6)	−0.00047 (5)	−0.00041 (5)
4.2		0.43511 (3)	0.61650 (3)	0.70679 (3)	0.006	0.00490 (11)	0.00749 (12)	0.00438 (10)	−0.00038 (11)	−0.00027 (9)	−0.00053 (10)
1.1	**O(2)**	0.39710 (3)	0.60581 (2)	0.51177 (2)	0.007	0.00848 (10)	0.00733 (10)	0.00475 (9)	0.00022 (7)	0.00067 (7)	0.00141 (7)
1.9		0.39708 (2)	0.605402 (19)	0.511388 (18)	0.007	0.00832 (7)	0.00698 (7)	0.00446 (7)	0.00043 (6)	0.00081 (6)	0.00143 (5)
4.2		0.39738 (3)	0.60435 (3)	0.51055 (3)	0.006	0.00818 (12)	0.00680 (11)	0.00413 (10)	0.00059 (10)	0.00082 (10)	0.00142 (10)
1.1	**O(3)**	0.31693 (2)	0.45685 (2)	0.63375 (2)	0.007	0.00656 (9)	0.00431 (9)	0.00869 (10)	−0.00027 (7)	−0.00102 (8)	0.00041 (7)
1.9		0.316429 (19)	0.456684 (17)	0.634052 (19)	0.006	0.00609 (7)	0.00411 (7)	0.00822 (7)	−0.00018 (5)	−0.00094 (6)	0.00031 (6)
4.2		0.31527 (3)	0.45636 (3)	0.63481 (3)	0.006	0.00552 (11)	0.00384 (11)	0.00800 (12)	−0.00024 (9)	−0.00090 (11)	0.00018 (10)
1.1	**O(4)**	0.23865 (2)	0.64264 (2)	0.64973 (2)	0.007	0.00487 (9)	0.00691 (9)	0.00868 (10)	0.00154 (7)	−0.00074 (8)	−0.00069 (7)
1.9		0.238883 (18)	0.643118 (19)	0.649745 (19)	0.007	0.00442 (7)	0.00686 (7)	0.00825 (7)	0.00147 (5)	−0.00071 (6)	−0.00049 (5)
4.2		0.23946 (3)	0.64427 (3)	0.64981 (3)	0.006	0.00440 (11)	0.00637 (11)	0.00796 (12)	0.00159 (10)	−0.00061 (10)	−0.00044 (10)
1.1	**Be(1)**	0.40586 (4)	0.37021 (4)	0.66999 (4)	0.006	0.00625 (16)	0.00621 (16)	0.00588 (16)	−0.00002 (13)	0.00000 (13)	−0.00035 (14)

1.9		0.40585 (3)	0.37023 (3)	0.67014 (3)	0.006	0.00606 (12)	0.00596 (13)	0.00589 (13)	−0.00016 (10)	0.00021 (10)	−0.00029 (11)
4.2		0.40580 (5)	0.37007 (5)	0.67034 (5)	0.006	0.00619 (19)	0.0063 (2)	0.00556 (19)	−0.00050 (19)	−0.00001 (18)	−0.00022 (19)
1.1		0.72985 (8)	0.72985 (8)	0.72985 (8)	0.011	0.0114 (3)	0.0114 (3)	0.0114 (3)	0.0007 (3)	0.0007 (3)	0.0007 (3)
1.9	**Li(1)**	0.72980 (6)	0.72980 (6)	0.72980 (6)	0.011	0.01061 (19)	0.01061 (19)	0.01061 (19)	0.0010 (2)	0.0010 (2)	0.0010 (2)
4.2		0.72957 (9)	0.72957 (9)	0.72957 (9)	0.01	0.0098 (3)	0.0098 (3)	0.0098 (3)	0.0008 (3)	0.0008 (3)	0.0008 (3)
1.1		0.02206 (8)	0.47794 (8)	0.52206 (8)	0.011	0.0113 (3)	0.0113 (3)	0.0113 (3)	0.0005 (3)	−0.0005 (3)	0.0005 (3)
1.9	**Li(2)**	0.02199 (6)	0.47801 (6)	0.52199 (6)	0.011	0.0108 (2)	0.0108 (2)	0.0108 (2)	0.0005 (2)	−0.0005 (2)	0.0005 (2)
4.2		0.02249 (9)	0.47751 (9)	0.52249 (9)	0.01	0.0099 (3)	0.0099 (3)	0.0099 (3)	−0.0002 (3)	0.0002 (3)	−0.0002 (3)

Table S3. Integrated charges and volumes of Bader ionic basins.

P	atom	q	Vtot_A 3	Lagran.
1.1	**Ca(1)**	1.60	12.45	2.89E-03
1.9		1.62	12.38	2.52E-03
4.2		1.66	12.12	2.23E-03
1.1	**Ca(2)**	1.63	12.45	2.75E-03
1.9		1.64	12.40	2.47E-03
4.2		1.66	12.15	2.03E-03
1.1	**Si(1)**	3.35	2.59	2.13E-03
1.9		3.31	2.60	2.03E-03
4.2		3.30	2.69	2.11E-03
1.1	**F(1)**	-0.67	13.90	1.83E-03
1.9		-0.56	13.65	1.76E-03
4.2		-0.57	13.32	1.68E-03
1.1	**F(2)**	-0.63	13.52	1.91E-03
1.9		-0.57	13.36	1.81E-03
4.2		-0.57	12.96	1.78E-03
1.1		-1.70	14.52	2.52E-03
1.9	**O(1)**	-1.73	14.61	1.95E-03
4.2		-1.78	14.38	1.33E-03
1.1		-1.66	15.40	3.32E-03
1.9	**O(2)**	-1.69	15.33	2.85E-03
4.2		-1.64	14.98	2.93E-03
1.1		-1.66	14.37	2.91E-03
1.9	**O(3)**	-1.67	14.23	2.82E-03
4.2		-1.72	13.93	1.96E-03
1.1		-1.72	15.72	3.26E-03
1.9	**O(4)**	-1.71	15.59	3.06E-03
4.2		-1.68	15.22	2.38E-03
1.1		1.70	1.66	8.56E-04
1.9	**Be(1)**	1.71	1.59	1.57E-03
4.2		1.71	1.55	1.85E-03
1.1		0.87	3.05	5.59E-04
1.9	**Li(1)**	0.87	3.07	5.03E-04
4.2		0.87	2.99	4.19E-04
1.1		0.87	3.05	6.86E-04
1.9	**Li(2)**	0.88	3.06	6.32E-04
4.2		0.88	2.99	5.60E-04

Table S4. List of bond/interatomic distances in hsiaghualite at various pressures.

**Bond**	**1.1 GPa**	**1.9 Gpa**	**$\Delta_{1.1-1.9}$**	**4.2 Gpa**	**$\Delta_{1.1-4.2}$**	**Shrink%(from 1.1 to 4.2Gpa)**
**Ca(1)-F(1)x2**	2.4201(3)	2.4141(3)	0.0114	2.3981(4)	0.0274	1.13%
**Ca(1)-O(1)x4**	2.4595(3)	2.4599(3)	0.0083	2.4329(4)	0.0353	1.43%
**Ca(1)-O(2)x2**	2.4668(4)	2.4699(3)	-0.0017	2.4643(4)	0.0039	0.16%
**Ca(2)-F(2)x2**	2.3898(3)	2.3846(3)	0.0111	2.3696(4)	0.0261	1.09%
**Ca(2)-O(3)x4**	2.6124(3)	2.6011(3)	0.0239	2.5625(4)	0.0625	2.38%
**Ca(2)-O(4)x2**	2.4773(3)	2.4763(3)	0.0044	2.472(4)	0.0087	0.35%
**Si(1)-O(1)**	1.6344(3)	1.6323(3)	0.0078	1.6313(5)	0.0088	0.54%
**Si(1)-O(2)**	1.6219(4)	1.6207(3)	0.0051	1.6172(5)	0.0086	0.53%
**Si(1)-O(3)**	1.621(3)	1.6194(3)	0.0029	1.6148(5)	0.0075	0.46%
**Si(1)-O(4)**	1.6184(3)	1.6246(3)	-0.0014	1.6132(5)	0.01	0.62%
**Be(1)-O(1)**	1.6238(6)	1.6221(6)	0.006	1.616(8)	0.0121	0.74%
**Be(1)-O(2)**	1.6265(6)	1.6242(5)	0.0057	1.6131(8)	0.0168	1.03%
**Be(1)-O(3)**	1.6601(6)	1.6576(5)	0.0036	1.6533(8)	0.0079	0.48%
**Be(1)-O(4)**	1.608(6)	1.6067(5)	0.0062	1.598(8)	0.0149	0.92%
**Li(1)-F(1)**	1.8758(11)	1.8717(10)	0.018	1.8521(13)	0.0376	1.99%
**Li(1)-O(4)x3**	1.9747(11)	1.9642(10)	0.0156	1.9401(13)	0.0397	2.01%
**Li(2)-F(2)**	1.8342(11)	1.8343(10)	0.0118	1.8113(13)	0.0348	1.89%
**Li(2)-O(2)x3**	1.9891(11)	1.9793(10)	0.018	1.9545(13)	0.0428	2.14%
**F(1)…O(1)**	2.8584(4)	2.8455(4)	0.0216	2.8118(6)	0.0553	1.93%
**F(2)…O(3)**	2.8618(4)	2.8498(4)	0.0199	2.8146(6)	0.0551	1.92%

Table S5. Bond critical point parameters and corresponding electronegativity. rho – electron density in e·Å^{-3} d2rho – laplacian of electron density in e·Å^{-5}. distances, Rij and bonded radii in Å.

bond	id	rho	d2rho	Rij	1st atom bonded radius	2nd atom bonded radius	Cation electronegativity (Boyd 1988)
**Ca(1)-F(1)_x2**	exp1.1GPa	0.164	3.575	2.4201	1.2444	1.1757	1.089441387
**Ca(1)-O(1)_x4**	exp1.1GPa	0.19	3.624	2.4595	1.2338	1.2257	1.123112696
**Ca(1)-O(2)_x2**	exp1.1GPa	0.195	3.502	2.4668	1.234	1.2328	1.12964054
**Ca(2)-F(2)_x2**	exp1.1GPa	0.176	3.95	2.3898	1.2289	1.1609	1.108880388
**Ca(2)-O(3)_x4**	exp1.1GPa	0.135	2.314	2.6124	1.3035	1.3089	1.029688519
**Ca(2)-O(4)_x2**	exp1.1GPa	0.183	3.319	2.4773	1.2433	1.234	1.112742534
**Si(1)-O(1)**	exp1.1GPa	1.07	10.103	1.6344	0.6893	0.9451	1.983308545
**Si(1)-O(2)**	exp1.1GPa	0.938	16.963	1.6219	0.6851	0.9368	1.919587272
**Si(1)-O(3)**	exp1.1GPa	0.938	17.737	1.621	0.6815	0.9396	1.917888071
**Si(1)-O(4)**	exp1.1GPa	1.028	14.294	1.6184	0.6847	0.9337	1.964666404
**Be(1)-O(1)**	exp1.1GPa	0.439	14.034	1.6238	0.5639	1.06	1.294468151
**Be(1)-O(2)**	exp1.1GPa	0.442	14.031	1.6265	0.5629	1.0636	1.296114935
**Be(1)-O(3)**	exp1.1GPa	0.407	11.987	1.6601	0.5742	1.0858	1.269569657
**Be(1)-O(4)**	exp1.1GPa	0.432	14.258	1.608	0.5646	1.0434	1.291211652
**Li(1)-F(1)**	exp1.1GPa	0.181	5.69	1.8758	0.7299	1.1459	0.886562319
**Li(1)-O(4)_x3**	exp1.1GPa	0.165	4.557	1.9747	0.751	1.2237	0.86317314
**Li(2)-F(2)**	exp1.1GPa	0.202	6.593	1.8342	0.7153	1.1189	0.911572103
**Li(2)-O(2)**	exp1.1GPa	0.158	4.331	1.9891	0.7567	1.2324	0.853900257
**F(1)…O(1)**	exp1.1GPa	0.073	0.877	2.8584	1.3913	1.467	
**F(2)…O(3)**	exp1.1GPa	0.07	0.896	2.8618	1.3927	1.4691	
**Ca(1)-F(1)_x2**	exp1.9GPa	0.164	3.634	2.4141	1.2427	1.1715	1.089740288
**Ca(1)-O(1)_x4**	exp1.9GPa	0.188	3.597	2.4599	1.2366	1.2233	1.120738066
**Ca(1)-O(2)_x2**	exp1.9GPa	0.19	3.457	2.4699	1.2391	1.2308	1.123131617
**Ca(2)-F(2)_x2**	exp1.9GPa	0.177	3.966	2.3846	1.2274	1.1573	1.110711159
**Ca(2)-O(3)_x4**	exp1.9GPa	0.137	2.407	2.6011	1.2994	1.3016	1.033809532
**Ca(2)-O(4)_x2**	exp1.9GPa	0.175	3.231	2.4763	1.2526	1.2237	1.102565637

**Si(1)-O(1)**	exp1.9GPa	1.083	10.697	1.6323	0.6882	0.9441	1.989197158
**Si(1)-O(2)**	exp1.9GPa	0.967	16.044	1.6207	0.6863	0.9344	1.935150411
**Si(1)-O(3)**	exp1.9GPa	0.978	16.064	1.6194	0.6821	0.9374	1.938689695
**Si(1)-O(4)**	exp1.9GPa	1.085	11.215	1.6246	0.6878	0.9367	1.991680035
**Be(1)-O(1)**	exp1.9GPa	0.437	14.428	1.6221	0.563	1.0591	1.292926036
**Be(1)-O(2)**	exp1.9GPa	0.428	13.694	1.6242	0.5657	1.0585	1.286810556
**Be(1)-O(3)**	exp1.9GPa	0.403	12.201	1.6576	0.5741	1.0835	1.266630646
**Be(1)-O(4)**	exp1.9GPa	0.438	14.644	1.6067	0.5623	1.0444	1.295102828
**Li(1)-F(1)**	exp1.9GPa	0.169	5.457	1.8717	0.7383	1.1334	0.873392404
**Li(1)-O(4)_x3**	exp1.9GPa	0.164	4.578	1.9642	0.7514	1.2128	0.862641342
**Li(2)-F(2)**	exp1.9GPa	0.196	6.451	1.8343	0.7188	1.1155	0.905422318
**Li(2)-O(2)**	exp1.9GPa	0.158	4.437	1.9793	0.7558	1.2235	0.854393677
**F(1)…O(1)**	exp1.9GPa	0.075	0.96	2.8455	1.3745	1.471	
**F(2)…O(3)**	exp1.9GPa	0.071	0.911	2.8498	1.3835	1.4663	
**Ca(1)-F(1)_x2**	exp4.2GPa	0.168	3.883	2.3981	1.2347	1.1634	1.096409527
**Ca(1)-O(1)_x4**	exp4.2GPa	0.192	3.794	2.4329	1.2289	1.2041	1.128006282
**Ca(1)-O(2)_x2**	exp4.2GPa	0.197	3.621	2.4643	1.2281	1.2361	1.131483965
**Ca(2)-F(2)_x2**	exp4.2GPa	0.175	4.084	2.3696	1.227	1.1426	1.109352878
**Ca(2)-O(3)_x4**	exp4.2GPa	0.143	2.678	2.5625	1.2851	1.2774	1.04595456
**Ca(2)-O(4)_x2**	exp4.2GPa	0.185	3.398	2.472	1.2402	1.2318	1.115673115
**Si(1)-O(1)**	exp4.2GPa	1.046	10.718	1.6313	0.693	0.9383	1.974711403
**Si(1)-O(2)**	exp4.2GPa	0.959	17.353	1.6172	0.683	0.9343	1.930139751
**Si(1)-O(3)**	exp4.2GPa	1.051	13.435	1.6148	0.6816	0.9332	1.97473748
**Si(1)-O(4)**	exp4.2GPa	1.126	10.437	1.6132	0.6851	0.9281	2.011347093
**Be(1)-O(1)**	exp4.2GPa	0.43	13.986	1.616	0.5662	1.0499	1.289310901
**Be(1)-O(2)**	exp4.2GPa	0.461	15.065	1.6131	0.5578	1.0553	1.309692076
**Be(1)-O(3)**	exp4.2GPa	0.43	13.101	1.6533	0.5672	1.086	1.28577153
**Be(1)-O(4)**	exp4.2GPa	0.454	15.333	1.598	0.5587	1.0393	1.306606266
**Li(1)-F(1)**	exp4.2GPa	0.177	5.76	1.8521	0.7326	1.1194	0.883978613
**Li(1)-O(4)_x3**	exp4.2GPa	0.174	4.949	1.9401	0.7436	1.1965	0.875771052
**Li(2)-F(2)**	exp4.2GPa	0.197	6.65	1.8113	0.7182	1.0931	0.908316634
**Li(2)-O(2)**	exp4.2GPa	0.175	5.023	1.9545	0.7414	1.2131	0.875628007
**F(1)…O(1)**	exp4.2GPa	0.075	1.04	2.8118	1.3594	1.4524	

**F(2)…O(3)**	exp4.2GPa	0.074	1.057	2.8146	1.3706	1.444	
**Ca(1)-F(1)_x2**	Teor0GPa	0.137	3.569	2.4248	1.2536	1.1712	1.043001524
**Ca(1)-O(1)_x4**	Teor0GPa	0.182	3.966	2.4329	1.2251	1.2078	1.112156818
**Ca(1)-O(2)_x2**	Teor0GPa	0.16	3.329	2.4878	1.2568	1.231	1.077765598
**Ca(2)-F(2)_x2**	Teor0GPa	0.149	3.753	2.398	1.2439	1.1541	1.066103068
**Ca(2)-O(3)_x4**	Teor0GPa	0.122	2.626	2.5854	1.2963	1.2891	1.005150273
**Ca(2)-O(4)_x2**	Teor0GPa	0.154	3.195	2.4903	1.2638	1.2265	1.068755256
**Si(1)-O(1)**	Teor0GPa	0.984	8.984	1.6719	0.7061	0.9657	1.942709223
**Si(1)-O(2)**	Teor0GPa	0.998	8.235	1.6481	0.7043	0.9437	1.953840153
**Si(1)-O(3)**	Teor0GPa	1.044	7.735	1.6468	0.7013	0.9456	1.974609119
**Si(1)-O(4)**	Teor0GPa	1.127	4.769	1.645	0.7051	0.9399	2.015263234
**Be(1)-O(1)**	Teor0GPa	0.449	14.174	1.6255	0.5615	1.064	1.300897902
**Be(1)-O(2)**	Teor0GPa	0.453	14.196	1.6295	0.5604	1.0691	1.303030014
**Be(1)-O(3)**	Teor0GPa	0.429	12.599	1.6663	0.5684	1.0979	1.284037443
**Be(1)-O(4)**	Teor0GPa	0.465	14.915	1.6075	0.5574	1.0501	1.313006351
**Li(1)-F(1)**	Teor0GPa	0.163	5.648	1.8706	0.7365	1.1342	0.865248683
**Li(1)-O(4)_x3**	Teor0GPa	0.163	4.898	1.9502	0.7471	1.2031	0.861511301
**Li(2)-F(2)**	Teor0GPa	0.182	6.197	1.8374	0.7243	1.113	0.889663694
**Li(2)-O(2)**	Teor0GPa	0.156	4.642	1.957	0.7533	1.2038	0.852719966
**F(1)…O(1)**	Teor0GPa	0.05	0.996	2.8403	1.3819	1.4584	
**F(2)…O(3)**	Teor0GPa	0.055	0.998	2.8467	1.3822	1.4646	
**Ca(1)-F(1)_x2**	Teor2.5GPa	0.151	3.82	2.4077	1.2415	1.1663	1.067933102
**Ca(1)-O(1)_x4**	Teor2.5GPa	0.191	4.053	2.4179	1.2203	1.1976	1.126315975
**Ca(1)-O(2)_x2**	Teor2.5GPa	0.173	3.559	2.4678	1.2432	1.2246	1.098251885
**Ca(2)-F(2)_x2**	Teor2.5GPa	0.159	4.043	2.3802	1.2321	1.1481	1.082964833
**Ca(2)-O(3)_x4**	Teor2.5GPa	0.126	2.71	2.5701	1.2911	1.279	1.013785858
**Ca(2)-O(4)_x2**	Teor2.5GPa	0.166	3.475	2.4706	1.2487	1.2219	1.087869807
**Si(1)-O(1)**	Teor2.5GPa	0.991	11.247	1.6475	0.6987	0.9488	1.947596491
**Si(1)-O(2)**	Teor2.5GPa	1	10.976	1.6343	0.6974	0.9369	1.954243613
**Si(1)-O(3)**	Teor2.5GPa	1.02	11.433	1.6321	0.6936	0.9385	1.962450384
**Si(1)-O(4)**	Teor2.5GPa	1.063	10.035	1.6287	0.6957	0.933	1.98471255
**Be(1)-O(1)**	Teor2.5GPa	0.453	14.692	1.6132	0.5599	1.0533	1.304607476
**Be(1)-O(2)**	Teor2.5GPa	0.461	14.936	1.6183	0.5578	1.0605	1.309136117

**Be(1)-O(3)**	Teor2.5GPa	0.426	12.77	1.6537	0.5686	1.0851	1.283112255
**Be(1)-O(4)**	Teor2.5GPa	0.481	16.031	1.5959	0.5528	1.0431	1.323983582
**Li(1)-F(1)**	Teor2.5GPa	0.176	6.088	1.8593	0.7276	1.1317	0.88117113
**Li(1)-O(4)_x3**	Teor2.5GPa	0.171	5.12	1.9383	0.7418	1.1965	0.871766006
**Li(2)-F(2)**	Teor2.5GPa	0.187	6.55	1.8263	0.7207	1.1057	0.895833683
**Li(2)-O(2)**	Teor2.5GPa	0.164	4.924	1.9456	0.7473	1.1983	0.863183949
**F(1)…O(1)**	Teor2.5GPa	0.058	1.051	2.8223	1.3754	1.4469	
**F(2)…O(3)**	Teor2.5GPa	0.056	1.044	2.826	1.3741	1.4519	
**Ca(1)-F(1)_x2**	Teor4.75GPa	0.157	3.978	2.394	1.2348	1.1592	1.07850351
**Ca(1)-O(1)_x4**	Teor4.75GPa	0.198	4.203	2.4037	1.2145	1.1892	1.136831581
**Ca(1)-O(2)_x2**	Teor4.75GPa	0.18	3.697	2.4536	1.237	1.2166	1.109422917
**Ca(2)-F(2)_x2**	Teor4.75GPa	0.165	4.199	2.3666	1.2259	1.1407	1.093249434
**Ca(2)-O(3)_x4**	Teor4.75GPa	0.132	2.823	2.5552	1.2846	1.2706	1.025853456
**Ca(2)-O(4)_x2**	Teor4.75GPa	0.173	3.613	2.4565	1.2418	1.2147	1.099221646
**Si(1)-O(1)**	Teor4.75GPa	0.989	12.665	1.638	0.6952	0.9428	1.946885497
**Si(1)-O(2)**	Teor4.75GPa	0.998	12.444	1.625	0.6939	0.931	1.95356336
**Si(1)-O(3)**	Teor4.75GPa	1.021	12.687	1.6226	0.6908	0.9317	1.96363611
**Si(1)-O(4)**	Teor4.75GPa	1.054	11.655	1.6195	0.6927	0.9268	1.980991763
**Be(1)-O(1)**	Teor4.75GPa	0.461	15.199	1.6041	0.5579	1.0462	1.31069701
**Be(1)-O(2)**	Teor4.75GPa	0.467	15.371	1.6091	0.5563	1.0528	1.313899822
**Be(1)-O(3)**	Teor4.75GPa	0.434	13.266	1.6442	0.5663	1.078	1.289358244
**Be(1)-O(4)**	Teor4.75GPa	0.491	16.609	1.5868	0.5506	1.0362	1.331123312
**Li(1)-F(1)**	Teor4.75GPa	0.182	6.304	1.8487	0.7241	1.1246	0.888721023
**Li(1)-O(4)_x3**	Teor4.75GPa	0.177	5.297	1.9271	0.7382	1.1888	0.879466016
**Li(2)-F(2)**	Teor4.75GPa	0.192	6.757	1.816	0.7177	1.0982	0.902027096
**Li(2)-O(2)**	Teor4.75GPa	0.17	5.113	1.9345	0.7432	1.1913	0.871009027
**F(1)…O(1)**	Teor4.75GPa	0.061	1.1	2.8061	1.3674	1.4387	
**F(2)…O(3)**	Teor4.75GPa	0.058	1.09	2.8099	1.3664	1.4435	
**Ca(1)-F(1)_x2**	Teor7.5GPa	0.154	4.114	2.3763	1.2321	1.1443	1.074792265
**Ca(1)-O(1)_x4**	Teor7.5GPa	0.209	4.608	2.3756	1.2009	1.1747	1.152461405
**Ca(1)-O(2)_x2**	Teor7.5GPa	0.168	3.512	2.4764	1.2482	1.2282	1.090370897
**Ca(2)-F(2)_x2**	Teor7.5GPa	0.17	4.286	2.3503	1.223	1.1273	1.102798377
**Ca(2)-O(3)_x4**	Teor7.5GPa	0.154	3.366	2.4918	1.2551	1.2367	1.066703629

**Ca(2)-O(4)_x2**	Teor7.5GPa	0.166	3.393	2.4745	1.2529	1.2216	1.088366077
**Si(1)-O(1)**	Teor7.5GPa	1.009	10.215	1.6584	0.7009	0.9574	1.955199122
**Si(1)-O(2)**	Teor7.5GPa	1.038	8.354	1.6346	0.7006	0.934	1.974186071
**Si(1)-O(3)**	Teor7.5GPa	1.096	7.61	1.6364	0.6969	0.9395	1.998838514
**Si(1)-O(4)**	Teor7.5GPa	1.181	4.63	1.6306	0.7006	0.93	2.039917284
**Be(1)-O(1)**	Teor7.5GPa	0.476	15.486	1.6048	0.5554	1.0494	1.320374574
**Be(1)-O(2)**	Teor7.5GPa	0.487	15.792	1.6051	0.5527	1.0524	1.327057551
**Be(1)-O(3)**	Teor7.5GPa	0.452	13.62	1.6466	0.563	1.0837	1.301292015
**Be(1)-O(4)**	Teor7.5GPa	0.501	16.651	1.5808	0.5495	1.0313	1.338173755
**Li(1)-F(1)**	Teor7.5GPa	0.186	6.572	1.8233	0.7212	1.1022	0.89497441
**Li(1)-O(4)_x3**	Teor7.5GPa	0.199	6.073	1.8836	0.7238	1.1598	0.906049413
**Li(2)-F(2)**	Teor7.5GPa	0.209	7.198	1.791	0.709	1.0821	0.921587853
**Li(2)-O(2)**	Teor7.5GPa	0.187	5.783	1.8878	0.7311	1.1567	0.893154225
**F(1)…O(1)**	Teor7.5GPa	0.058	1.274	2.7461	1.3358	1.4103	
**F(2)…O(3)**	Teor7.5GPa	0.068	1.27	2.7521	1.3385	1.4136	

Table S6. List of angles O-Si-O angles in SiO4 and O-Be-O angles in BeO4 tetrahedra

**O-Si-O**		**1.1 Gpa**	**1.9 Gpa**	**4.2 Gpa**
O4	O3	106.52(1)	106.48(1)	106.43(2)
O4	O2	115.26(2)	115.19(1)	115.16(2)
O4	O1	107.71(1)	107.67(1)	107.61(2)
O3	O2	111.29(1)	111.35(1)	111.47(2)
O3	O1	112.81(1)	112.84(1)	112.92(2)
O2	O1	103.35(2)	103.41(1)	103.36(2)
**O-Be_O**				
O4	O1	113.37(3)	113.32(3)	113.27(4)
O4	O2	116.41(3)	116.31(3)	116.48(4)
O4	O3	102.46(3)	102.42(3)	102.37(4)
O1	O2	106.76(3)	106.84(3)	106.83(4)
O1	O3	110.72(3)	110.77(3)	110.77(4)
O2	O3	106.89(3)	106.96(3)	106.88(4)

Table S7. List of Si-O-Be angles bwtween *T*O4 tetrahedra forming hsianghualite infinite framework.

**angle**	**1.1 Gpa**	**1.9 Gpa**	**4.2 Gpa**
Si-O(1)-Be	120.92(2)	120.61(2)	119.76(3)
Si-O(2)-Be	139.29(3)	139.19(2)	138.55(3)
Si-O(3)-Be	121.45(2)	121.04(2)	120.21(3)
Si-O(4)-Be	138.51(3)	138.37(2)	137.89(3)

3. Theoretical calculations.

Experimental X-ray measurements under pressure were accompanied by theoretical calculations. We produced lists of theoretical structure factors, which were further used for multipole model refinement against theoretical data. The approach provides benchmark values and cross-check for multipole model refined on pure experimental data collected at the synchrotron. We have used CRYSTAL17[50,51] software which is devoted to periodic systems calculations and allows also for charge density topological analysis. In our calculations we have used B3LYP[52,53] exchange-correlation functional corrected for dispersion by Grimme's D3[54] correction in conjunction with the pob_TZVP_rev2 basis sets.[55] Atomic positions and unit cell parameters were refined under several pressures in range from 0 to 7.5 GPa and theoretical dynamic structure factors were calculated in CRYSTAL17[56]. Then used to refine a multipolar model of the electron density using the XD2016 program package in identical steps as the experimental data.[57]

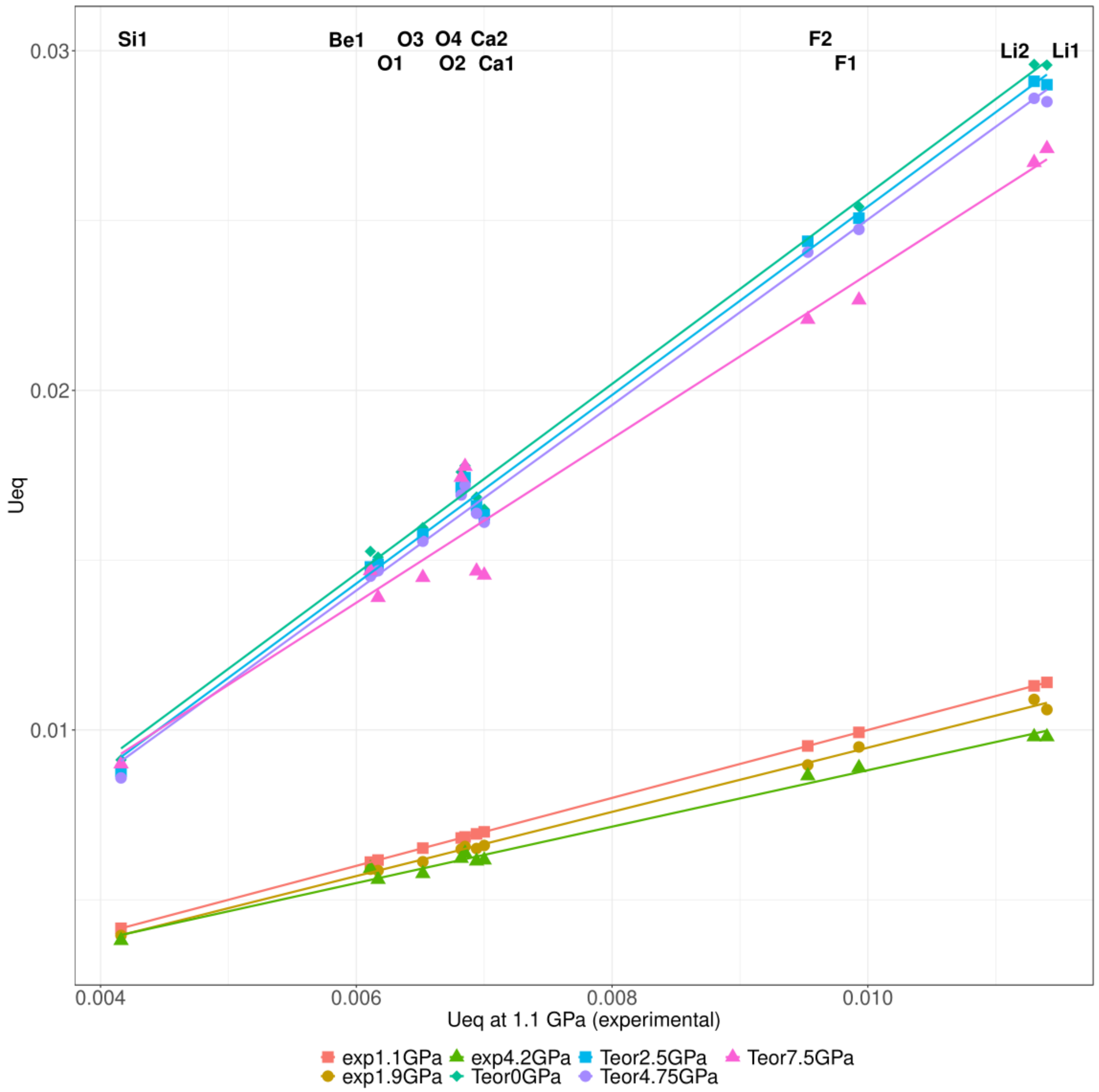

Figure S1. The equivalent isotropic values (*U*eq) of displacement parameters for all atoms plotted vs. *U*eq at 1.1 GPa. The points are presented for atoms in the following order starting from left: Si1, Be1, O1, O3, O4, O2, Ca2, Ca1, F2, F1, Li2, Li1.

Figure S2. Ionic basins overlay and visualisations of differences in the total electron densities of the central ions in hsianghualite

Ca1

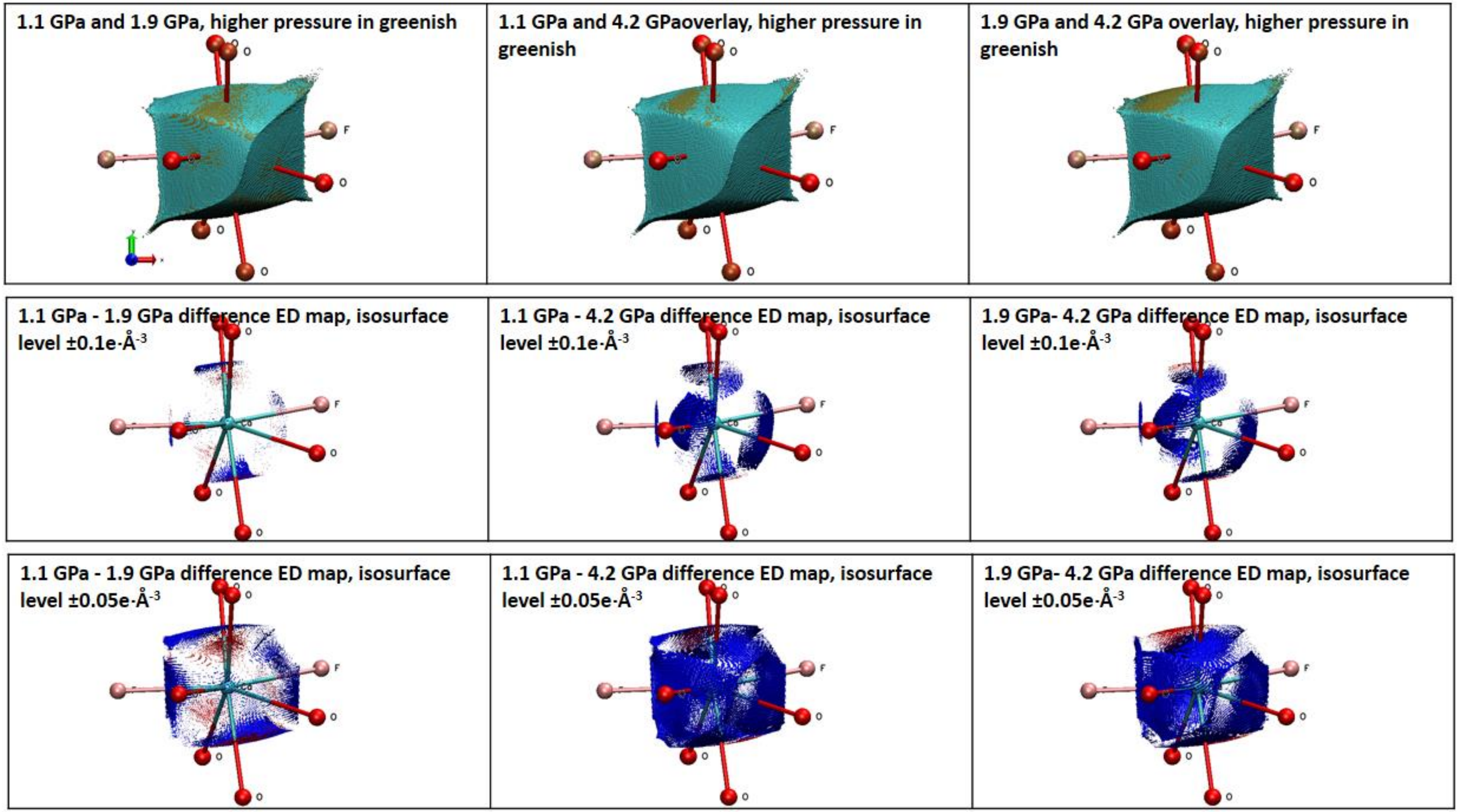

Ca2

1.1 GPa and 1.9 GPa, higher pressure in greenish

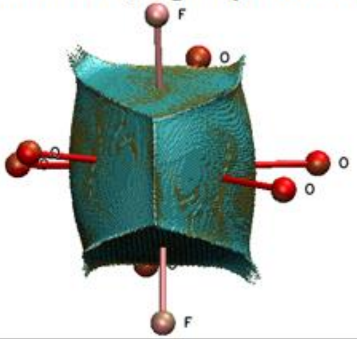

1.1 GPa and 4.2 GPaoverlay, higher pressure in greenish

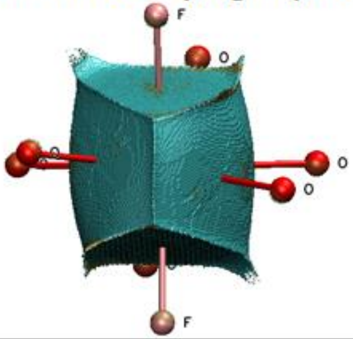

1.9 GPa and 4.2 GPa overlay, higher pressure in greenish

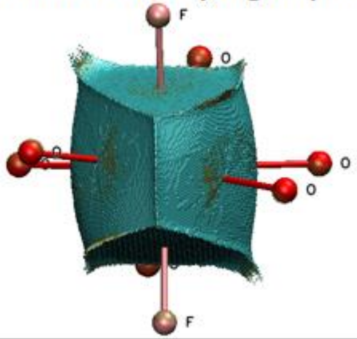

1.1 GPa - 1.9 GPa difference ED map, isosurface level ±0.1e·Å^{-3}

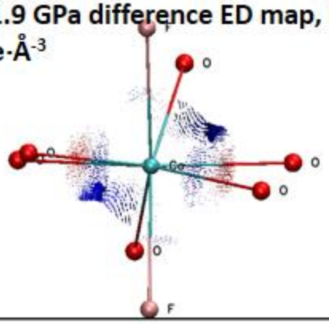

1.1 GPa - 4.2 GPa difference ED map, isosurface level ±0.1e·Å^{-3}

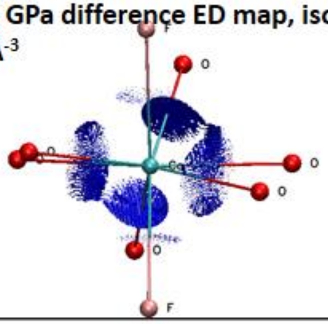

1.9 GPa- 4.2 GPa difference ED map, isosurface level ±0.1e·Å^{-3}

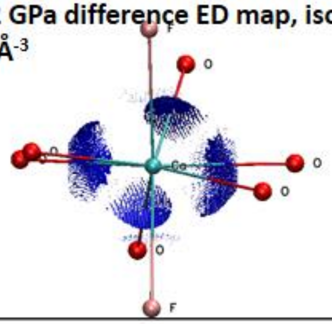

1.1 GPa - 1.9 GPa difference ED map, isosurface level ±0.05e·Å^{-3}

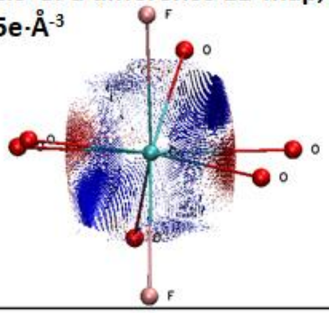

1.1 GPa - 4.2 GPa difference ED map, isosurface level ±0.05e·Å^{-3}

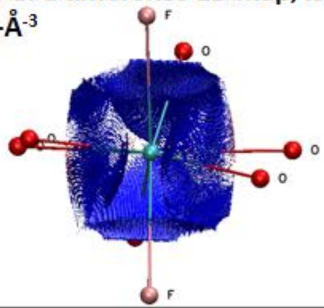

1.9 GPa- 4.2 GPa difference ED map, isosurface level ±0.05e·Å^{-3}

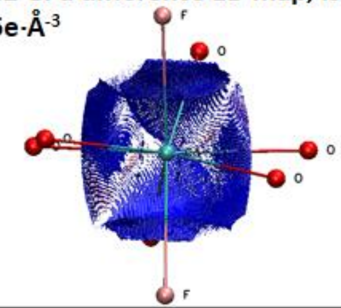

Si1

1.1 GPa and 1.9 GPa, higher pressure in greenish

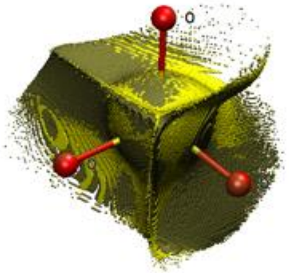

1.1 GPa and 4.2 GPaoverlay, higher pressure in greenish

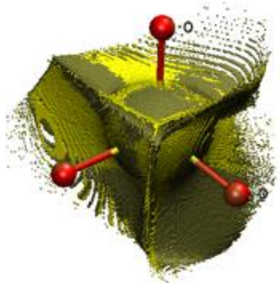

1.9 GPa and 4.2 GPa overlay, higher pressure in greenish

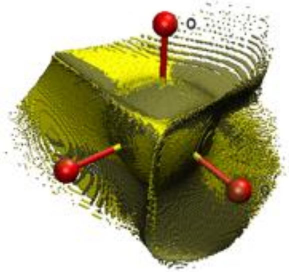

1.1 GPa - 1.9 GPa difference ED map, isosurface level ±0.1e·Å^{-3}

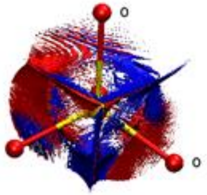

1.1 GPa - 4.2 GPa difference ED map, isosurface level ±0.1e·Å^{-3}

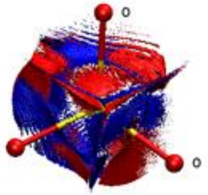

1.9 GPa- 4.2 GPa difference ED map, isosurface level ±0.1e·Å^{-3}

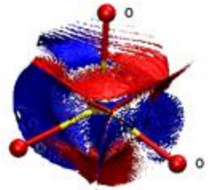

1.1 GPa - 1.9 GPa difference ED map, isosurface level ±0.05e·Å^{-3}

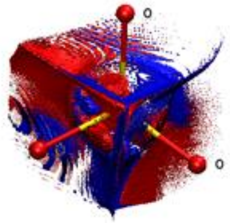

1.1 GPa - 4.2 GPa difference ED map, isosurface level ±0.05e·Å^{-3}

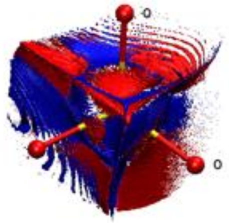

1.9 GPa- 4.2 GPa difference ED map, isosurface level ±0.05e·Å^{-3}

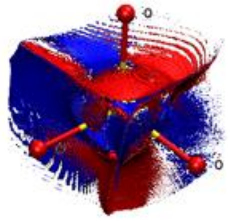

F1

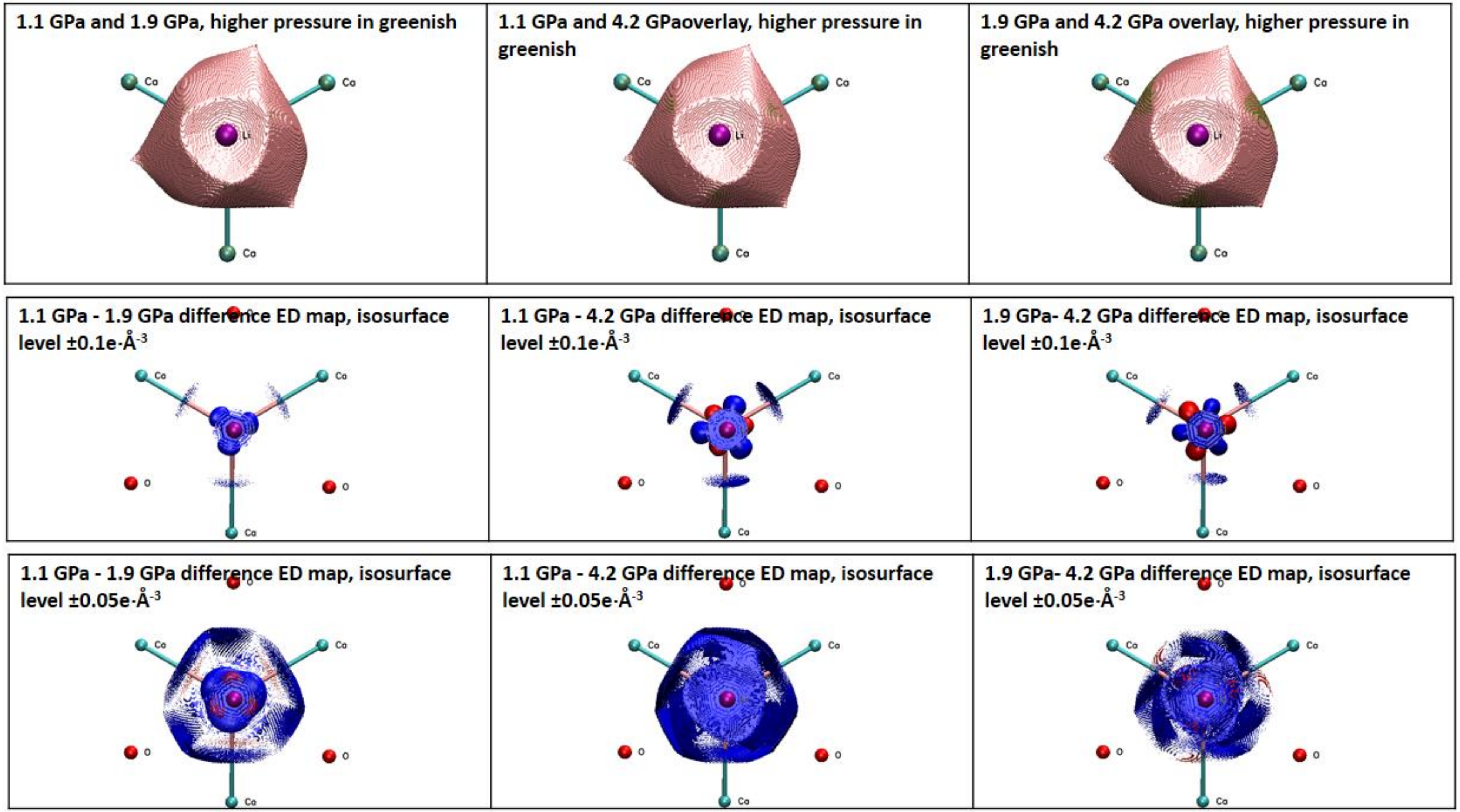
1.1 GPa and 1.9 GPa, higher pressure in greenish
1.1 GPa and 4.2 GPaoverlay, higher pressure in greenish
1.9 GPa and 4.2 GPa overlay, higher pressure in greenish
1.1 GPa - 1.9 GPa difference ED map, isosurface level ±0.1e·Å^{-3}
1.1 GPa - 4.2 GPa difference ED map, isosurface level ±0.1e·Å^{-3}
1.9 GPa- 4.2 GPa difference ED map, isosurface level ±0.1e·Å^{-3}
1.1 GPa - 1.9 GPa difference ED map, isosurface level ±0.05e·Å^{-3}
1.1 GPa - 4.2 GPa difference ED map, isosurface level ±0.05e·Å^{-3}
1.9 GPa- 4.2 GPa difference ED map, isosurface level ±0.05e·Å^{-3}
Ca
Li
O

F2

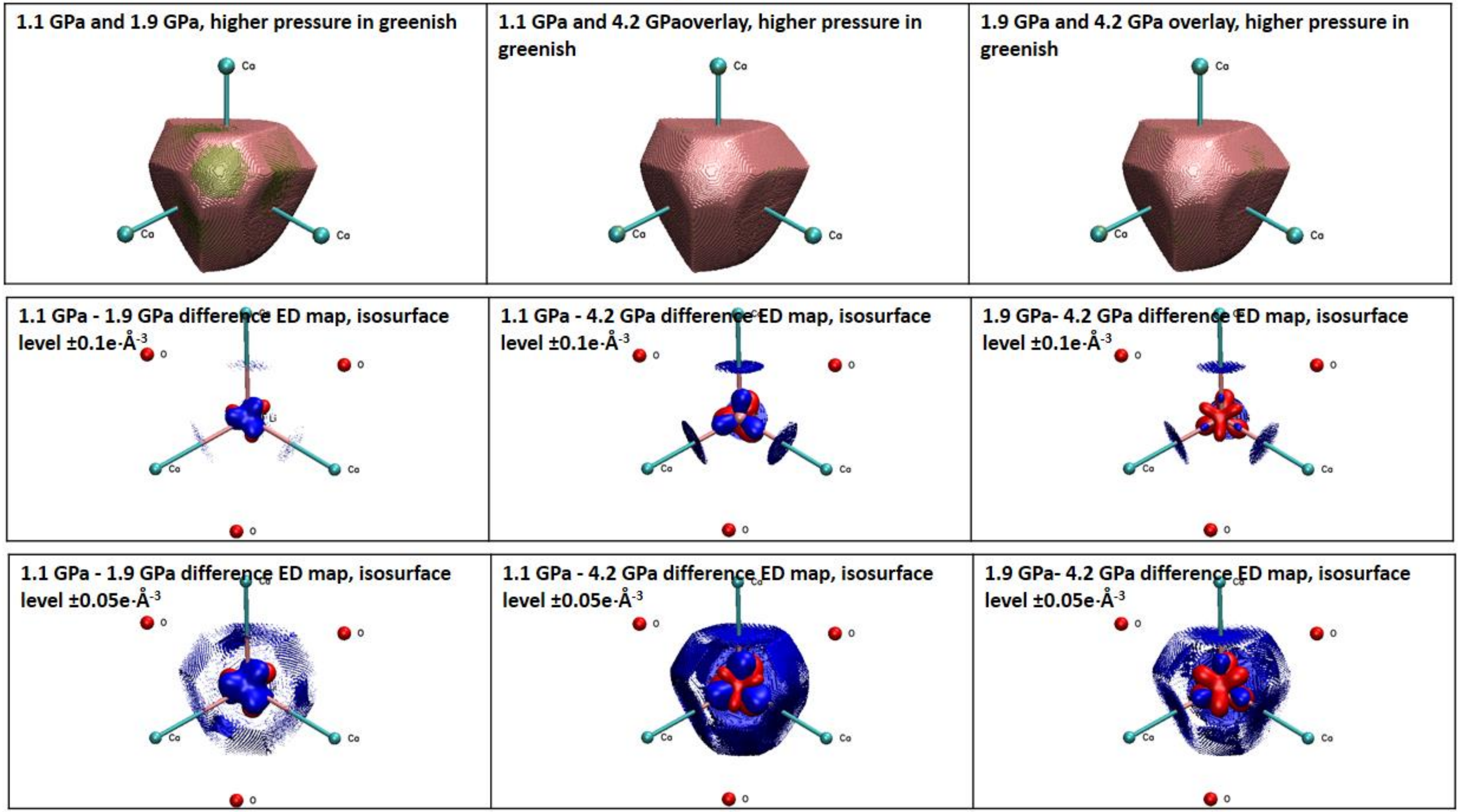
1.1 GPa and 1.9 GPa, higher pressure in greenish
1.1 GPa and 4.2 GPaoverlay, higher pressure in greenish
1.9 GPa and 4.2 GPa overlay, higher pressure in greenish
1.1 GPa - 1.9 GPa difference ED map, isosurface level ±0.1e·Å^{-3}
1.1 GPa - 4.2 GPa difference ED map, isosurface level ±0.1e·Å^{-3}
1.9 GPa- 4.2 GPa difference ED map, isosurface level ±0.1e·Å^{-3}
1.1 GPa - 1.9 GPa difference ED map, isosurface level ±0.05e·Å^{-3}
1.1 GPa - 4.2 GPa difference ED map, isosurface level ±0.05e·Å^{-3}
1.9 GPa- 4.2 GPa difference ED map, isosurface level ±0.05e·Å^{-3}
Ca
O
Li

O1

1.1 GPa and 1.9 GPa, higher pressure in greenish

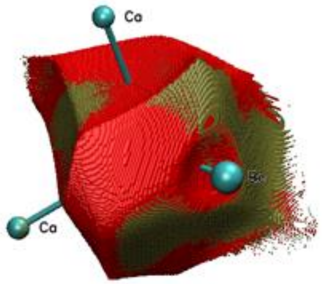

1.1 GPa and 4.2 GPaoverlay, higher pressure in greenish

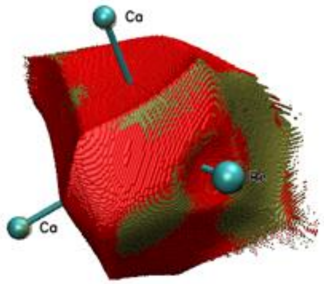

1.9 GPa and 4.2 GPa overlay, higher pressure in greenish

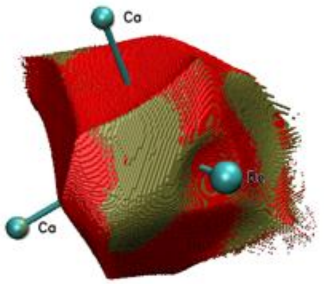

1.1 GPa - 1.9 GPa difference ED map, isosurface level ±0.1e·Å^{-3}

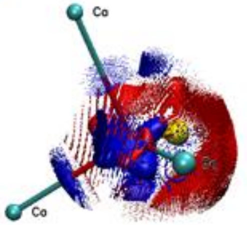

1.1 GPa - 4.2 GPa difference ED map, isosurface level ±0.1e·Å^{-3}

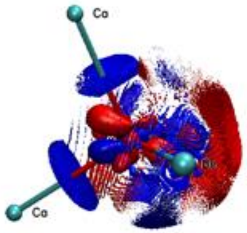

1.9 GPa- 4.2 GPa difference ED map, isosurface level ±0.1e·Å^{-3}

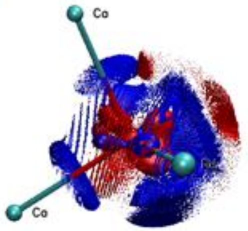

1.1 GPa - 1.9 GPa difference ED map, isosurface level ±0.05e·Å^{-3}

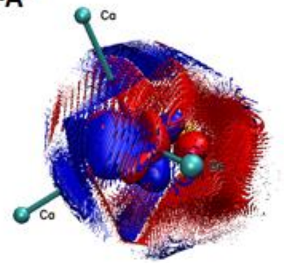

1.1 GPa - 4.2 GPa difference ED map, isosurface level ±0.05e·Å^{-3}

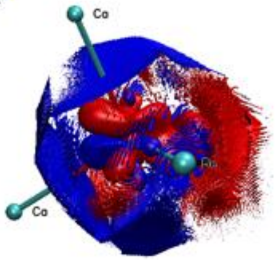

1.9 GPa- 4.2 GPa difference ED map, isosurface level ±0.05e·Å^{-3}

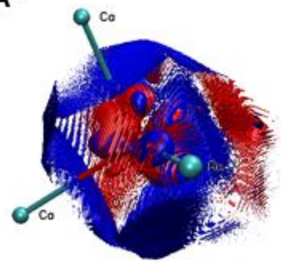

O2

1.1 GPa and 1.9 GPa, higher pressure in greenish

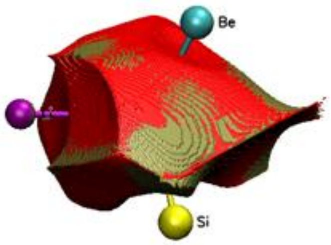

1.1 GPa and 4.2 GPaoverlay, higher pressure in greenish

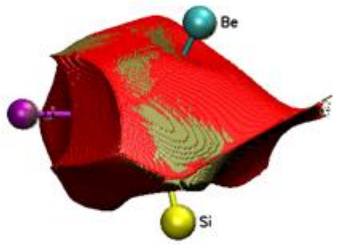

1.9 GPa and 4.2 GPa overlay, higher pressure in greenish

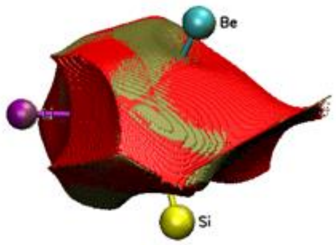

1.1 GPa - 1.9 GPa difference ED map, isosurface level ±0.1e·Å^{-3}

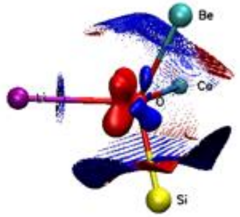

1.1 GPa - 4.2 GPa difference ED map, isosurface level ±0.1e·Å^{-3}

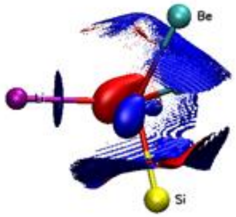

1.9 GPa- 4.2 GPa difference ED map, isosurface level ±0.1e·Å^{-3}

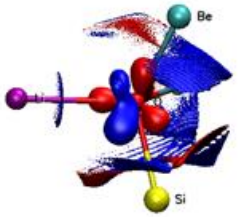

1.1 GPa - 1.9 GPa difference ED map, isosurface level ±0.05e·Å^{-3}

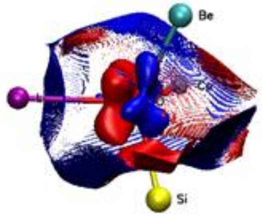

1.1 GPa - 4.2 GPa difference ED map, isosurface level ±0.05e·Å^{-3}

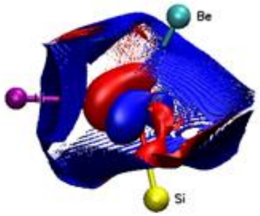

1.9 GPa- 4.2 GPa difference ED map, isosurface level ±0.05e·Å^{-3}

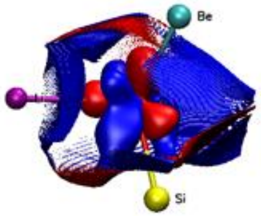

O3

1.1 GPa and 1.9 GPa, higher pressure in greenish 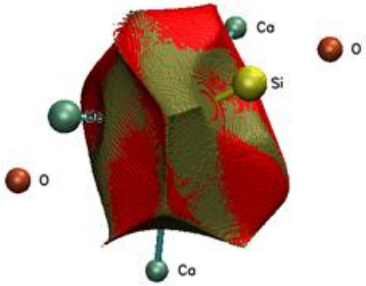	1.1 GPa and 4.2 GPaoverlay, higher pressure in greenish 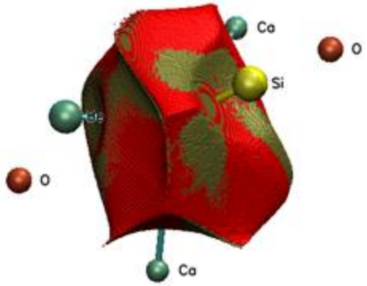	1.9 GPa and 4.2 GPa overlay, higher pressure in greenish 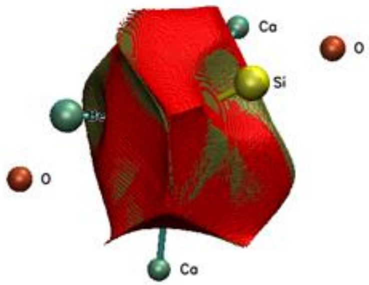
1.1 GPa - 1.9 GPa difference ED map, isosurface level $\pm0.1e\cdot\text{Å}^{-3}$ 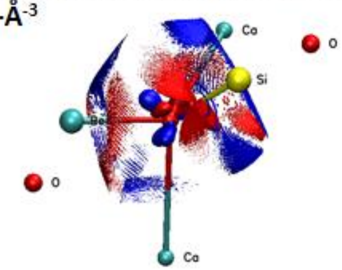	1.1 GPa - 4.2 GPa difference ED map, isosurface level $\pm0.1e\cdot\text{Å}^{-3}$ 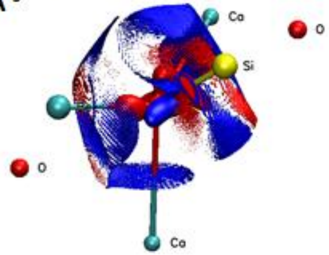	1.9 GPa- 4.2 GPa difference ED map, isosurface level $\pm0.1e\cdot\text{Å}^{-3}$ 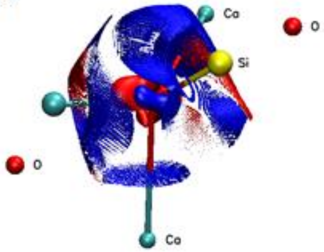
1.1 GPa - 1.9 GPa difference ED map, isosurface level $\pm0.05e\cdot\text{Å}^{-3}$ 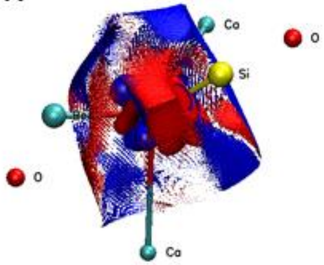	1.1 GPa - 4.2 GPa difference ED map, isosurface level $\pm0.05e\cdot\text{Å}^{-3}$ 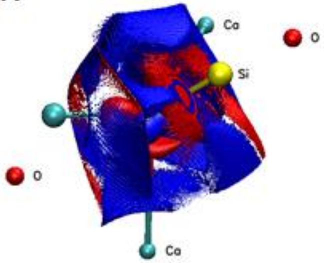	1.9 GPa- 4.2 GPa difference ED map, isosurface level $\pm0.05e\cdot\text{Å}^{-3}$ 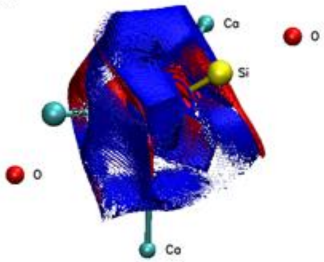

O4

1.1 GPa and 1.9 GPa, higher pressure in greenish

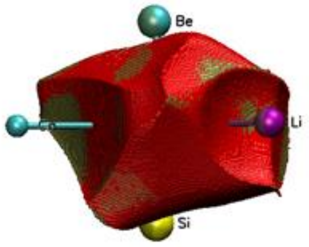

1.1 GPa and 4.2 GPaoverlay, higher pressure in greenish

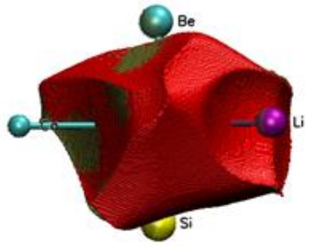

1.9 GPa and 4.2 GPa overlay, higher pressure in greenish

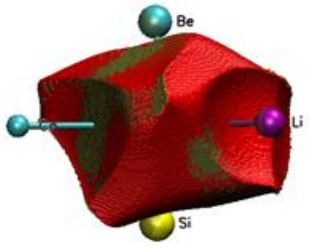

1.1 GPa - 1.9 GPa difference ED map, isosurface level ±0.1e·Å^{-3}

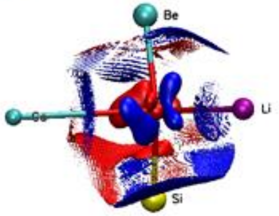

1.1 GPa - 4.2 GPa difference ED map, isosurface level ±0.1e·Å^{-3}

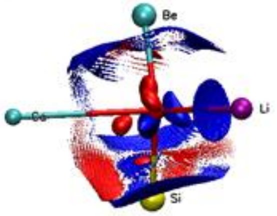

1.9 GPa- 4.2 GPa difference ED map isosurface level ±0.1e·Å^{-3}

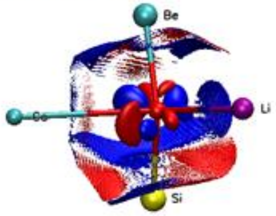

1.1 GPa - 1.9 GPa difference ED map, isosurface level ±0.05e·Å^{-3}

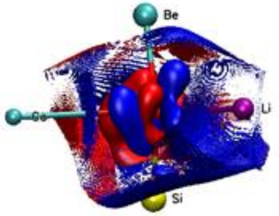

1.1 GPa - 4.2 GPa difference ED map, isosurface level ±0.05e·Å^{-3}

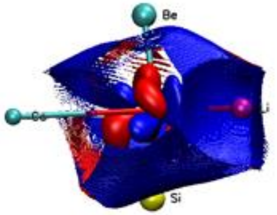

1.9 GPa- 4.2 GPa difference ED map, isosurface level ±0.05e·Å^{-3}

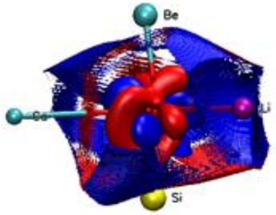

Be1

1.1 GPa and 1.9 GPa, higher pressure in greenish

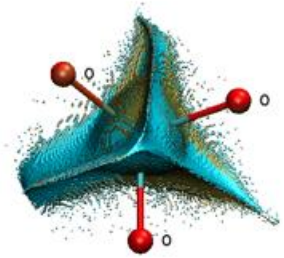

1.1 GPa and 4.2 GPaoverlay, higher pressure in greenish

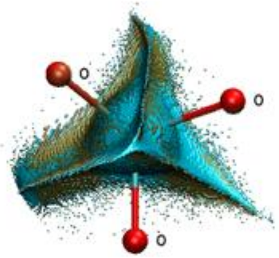

1.9 GPa and 4.2 GPa overlay, higher pressure in greenish

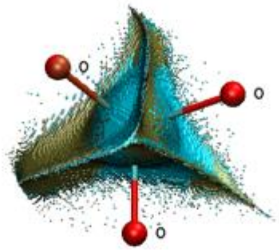

1.1 GPa - 1.9 GPa difference ED map, isosurface level ±0.1e·Å^{-3}

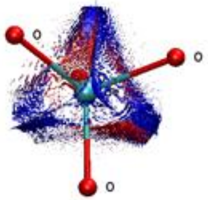

1.1 GPa - 4.2 GPa difference ED map, isosurface level ±0.1e·Å^{-3}

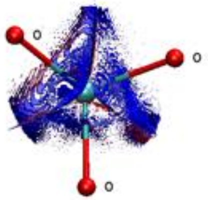

1.9 GPa- 4.2 GPa difference ED map, isosurface level ±0.1e·Å^{-3}

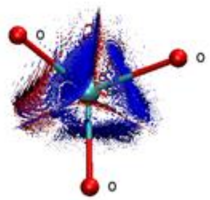

1.1 GPa - 1.9 GPa difference ED map, isosurface level ±0.05e·Å^{-3}

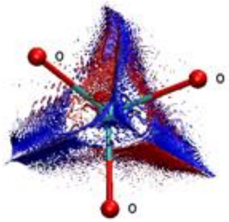

1.1 GPa - 4.2 GPa difference ED map, isosurface level ±0.05e·Å^{-3}

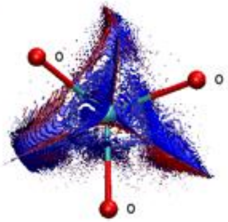

1.9 GPa- 4.2 GPa difference ED map, isosurface level ±0.05e·Å^{-3}

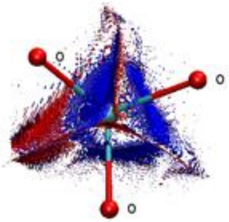

Li1

1.1 GPa and 1.9 GPa, higher pressure in greenish

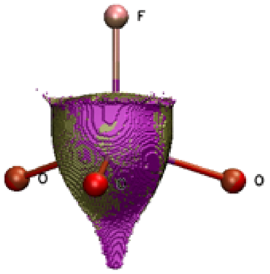

1.1 GPa and 4.2 GPaoverlay, higher pressure in greenish

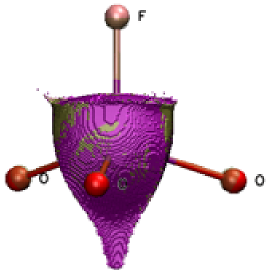

1.9 GPa and 4.2 GPa overlay, higher pressure in greenish

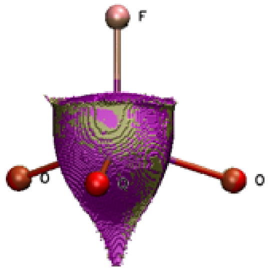

1.1 GPa - 1.9 GPa difference ED map, isosurface level ±0.1e·Å^{-3}

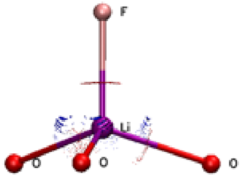

1.1 GPa - 4.2 GPa difference ED map, isosurface level ±0.1e·Å^{-3}

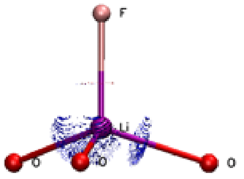

1.9 GPa- 4.2 GPa difference ED map, isosurface level ±0.1e·Å^{-3}

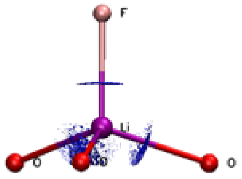

1.1 GPa - 1.9 GPa difference ED map, isosurface level ±0.05e·Å^{-3}

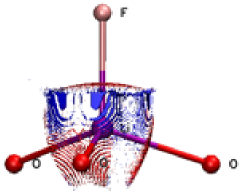

1.1 GPa - 4.2 GPa difference ED map, isosurface level ±0.05e·Å^{-3}

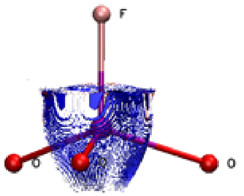

1.9 GPa- 4.2 GPa difference ED map, isosurface level ±0.05e·Å^{-3}

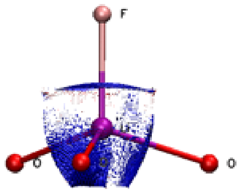

Li2

1.1 GPa and 1.9 GPa, higher pressure in greenish

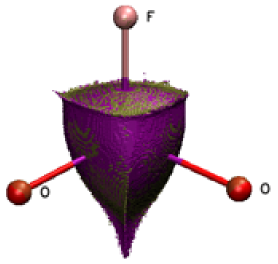

1.1 GPa and 4.2 GPaoverlay, higher pressure in greenish

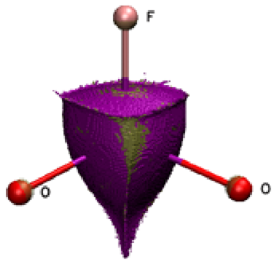

1.9 GPa and 4.2 GPa overlay, higher pressure in greenish

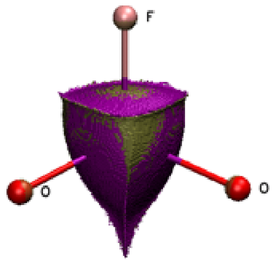

1.1 GPa - 1.9 GPa difference ED map, isosurface level ±0.1e·Å^{-3}

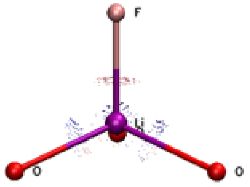

1.1 GPa - 4.2 GPa difference ED map, isosurface level ±0.1e·Å^{-3}

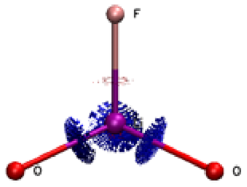

1.9 GPa- 4.2 GPa difference ED map, isosurface level ±0.1e·Å^{-3}

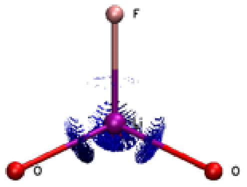

1.1 GPa - 1.9 GPa difference ED map, isosurface level ±0.05e·Å^{-3}

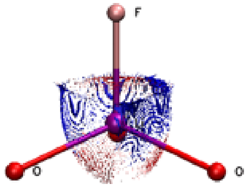

1.1 GPa - 4.2 GPa difference ED map, isosurface level ±0.05e·Å^{-3}

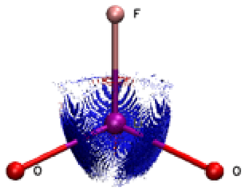

1.9 GPa- 4.2 GPa difference ED map, isosurface level ±0.05e·Å^{-3}

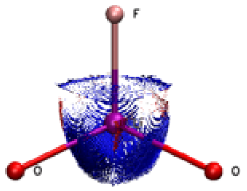

List of all remaining angles

List of angles 1.1 GPa

2.8 Angstrom Coordination Sphere Around Atom I = Ca1 [ARU = 1555.04] 1/2 3/4 0.58643 6.4137 9.6206 7.5224

Nr	d(I,J)	To	Atom J	Symm_Oper. on Atom J	ARU(J)	Type	Phi	Mu	X	Y	Z	XO	YO	ZO
1	2.4191(3)	<<	F1	[	= 02]		-35.72	18.23	0.64542	0.64542	0.64542	8.2791	8.2791	8.2791
2	2.4191(3)	<<	F1_a	[1-x,3/2-y,z	= 2665.02]		144.28	18.23	0.35458	0.85458	0.64542	4.5483	10.9621	8.2791
3	2.4574(3)	<<	O1	[	= 01]		-116.59	39.05	0.43340	0.61696	0.70712	5.5594	7.9140	9.0705
4	2.4574(3)	<<	O1_b	[1-x,3/2-y,z	= 2665.01]		63.41	39.05	0.56660	0.88304	0.70712	7.2680	11.3271	9.0705
5	2.4658(3)	<<	O2	[	= 01]		-125.51	-22.85	0.39710	0.60581	0.51177	5.0938	7.7710	6.5647
6	2.4658(3)	<<	O2_b	[1-x,3/2-y,z	= 2665.01]		54.49	-22.85	0.60290	0.89419	0.51177	7.7337	11.4702	6.5647
7	2.5311(3)	<<	O1_c	[y,z,x	= 9555.01]		-20.13	-50.85	0.61696	0.70712	0.43340	7.9140	9.0705	5.5594
8	2.5311(3)	<<	O1_d	[1-y,3/2-z,x	= 12665.01]		159.87	-50.85	0.38304	0.79288	0.43340	4.9134	10.1706	5.5594

Angles (Degrees) At1...V...At2 with Vertex V = Ca1

F1	, F1_a	143.54(1)	F1	, O1	71.69(1)	F1	, O1_b	85.41(1)	F1	, O2	96.79(1)
F1	, O2_b	97.16(1)	F1	, O1_c	70.43(1)	F1	, O1_d	145.10(1)	F1_a	, O1	85.41(1)
F1_a	, O1_b	71.69(1)	F1_a	, O2	97.16(1)	F1_a	, O2_b	96.79(1)	F1_a	, O1_c	145.10(1)
F1_a	, O1_d	70.43(1)	O1	, O1_b	101.90(1)	O1	, O2	62.46(1)	O1	, O2_b	162.11(1)
O1	, O1_c	122.94(1)	O1	, O1_d	115.69(1)	O1_b	, O2	162.11(1)	O1_b	, O2_b	62.46(1)
O1_b	, O1_c	115.69(1)	O1_b	, O1_d	122.94(1)	O2	, O2_b	134.29(1)	O2	, O1_c	81.55(1)
O2	, O1_d	62.90(1)	O2_b	, O1_c	62.90(1)	O2_b	, O1_d	81.55(1)	O1_c	, O1_d	78.30(1)

==

2.8 Angstrom Coordination Sphere Around Atom I = Ca2 [ARU = 1555.05] 0.15580 1/2 3/4 1.9986 6.4137 9.6206

Nr	d(I,J)	To	Atom J	Symm_Oper. on Atom J	ARU(J)	Type	Phi	Mu	X	Y	Z	XO	YO	ZO
1	2.3895(3)	<<	F2	[	= 03]		-116.07	-51.30	0.10462	0.39538	0.60462	1.3420	5.0717	7.7557
2	2.3895(3)	<<	F2_a	[x,1-y,3/2-z	= 3566.03]		116.07	51.30	0.10462	0.60462	0.89538	1.3420	7.7557	11.4854
3	2.4017(3)	<<	O3_d	[1/2-y,1-z,1/2+x	= 24565.01]		-130.11	20.94	0.04315	0.36626	0.81693	0.5535	4.6982	10.4791
4	2.4017(3)	<<	O3_c	[1/2-y,z,1-x	= 23556.01]		130.11	-20.94	0.04315	0.63374	0.68307	0.5535	8.1293	8.7620
5	2.4762(3)	<<	O4_b	[x,1-y,3/2-z	= 3566.01]		-59.85	31.29	0.23865	0.35736	0.85027	3.0613	4.5840	10.9068
6	2.4762(3)	<<	O4	[	= 01]		59.85	-31.29	0.23865	0.64264	0.64973	3.0613	8.2434	8.3344
7	2.6081(3)	<<	O3	[	= 01]		-14.99	-34.88	0.31693	0.45685	0.63374	4.0654	5.8602	8.1293
8	2.6081(3)	<<	O3_b	[x,1-y,3/2-z	= 3566.01]		14.99	34.88	0.31693	0.54315	0.86626	4.0654	6.9672	11.1119

Angles (Degrees) At1...V...At2 with Vertex V = Ca2

F2	,	F2_a	148.10(1)	F2	,	O3_d	73.29(1)	F2	,	O3_c	87.53(1)	F2	,	O4_b	96.22(1)
F2	,	O4	97.33(1)	F2	,	O3	69.65(1)	F2	,	O3_b	141.55(1)	F2_a	,	O3_d	87.53(1)
F2_a	,	O3_c	73.29(1)	F2_a	,	O4_b	97.33(1)	F2_a	,	O4	96.22(1)	F2_a	,	O3	141.55(1)
F2_a	,	O3_b	69.65(1)	O3_d	,	O3_c	106.02(1)	O3_d	,	O4_b	62.92(1)	O3_d	,	O4	166.34(1)
O3_d	,	O3	121.98(1)	O3_d	,	O3_b	115.09(1)	O3_c	,	O4_b	166.34(1)	O3_c	,	O4	62.92(1)
O3_c	,	O3	115.09(1)	O3_c	,	O3_b	121.98(1)	O4_b	,	O4	129.17(1)	O4_b	,	O3	78.47(1)
O4_b	,	O3_b	61.30(1)	O4	,	O3	61.30(1)	O4	,	O3_b	78.47(1)	O3	,	O3_b	75.17(1)

==

2.8 Angstrom Coordination Sphere Around Atom I = Si1 [ARU = 1555.01] 0.34477 0.57956 0.62379 4.4225 7.4343 8.0016

Nr	d(I,J)	To	Atom J	Symm_Oper. on Atom J	ARU(J)	Type	Phi	Mu	X	Y	Z	XO	YO	ZO

```
------------------------------------------------------------------------------------------------------------------------------------
 1   1.6181(3) --  O4                                     Intra 149.27  11.87   0.23865  0.64264  0.64973    3.0613  8.2434  8.3344
 2   1.6191(3) --  O3                                     Intra-102.78   4.52   0.31693  0.45685  0.63374    4.0654  5.8602  8.1293
 3   1.6213(3) --  O2                                     Intra  26.64 -62.41   0.39710  0.60581  0.51177    5.0938  7.7710  6.5647
 4   1.6326(3) --  O1                                     Intra  22.88  40.90   0.43340  0.61696  0.70712    5.5594  7.9140  9.0705

Tetrahedral Volume  2.177 Ang**3, Quadratic Elongation  1.005, Angle Variance  19.44 Deg**2 [K.Robinson +, Science 1971,172,567-570]

Tau(4)-Descriptor for 4-Coordination (L.Yang, D.R.Powell, R.P.Houser, Dalton Trans. (2007), 955-964)
------------------------------------------------------------------------------------------------------------------------------------
Tau(4) = (360 - (Beta + Alpha)) / 141 = 0.94 :: (Extreme Forms: 0.00 for SQP and 1.00 for TET; 0.85 for TRP)

Angles (Degrees)  At1...V...At2 with Vertex V =  Si1
------------------------------------------------------------------------------------------------------------------------------------
 O4     ,  O3        106.52(1)     O4     ,  O2        115.26(2)     O4     ,  O1        107.71(1)     O3     ,  O2        111.29(1)
 O3     ,  O1        112.81(1)     O2     ,  O1        103.35(2)

====================================================================================================================================
 2.8 Angstrom Coordination Sphere Around Atom I = F1     [ARU = 1555.02]         0.64542  0.64542  0.64542    8.2791  8.2791  8.2791
------------------------------------------------------------------------------------------------------------------------------------
 Nr     d(I,J) To  Atom J  Symm_Oper. on Atom J    ARU(J)  Type     Phi      Mu         X        Y        Z        XO      YO      ZO
------------------------------------------------------------------------------------------------------------------------------------
 1  1.8759(11) <<  Li1    [                    =       07]        45.00  35.26   0.72985  0.72985  0.72985    9.3621  9.3621  9.3621
 2   2.4191(3) <<  Ca1_b  [y,z,x               =  9555.04]       -29.43 -50.45       3/4  0.58643      1/2    9.6206  7.5224  6.4137
 3   2.4191(3) <<  Ca1_a  [z,x,y               =  5555.04]      -112.08  33.68   0.58643      1/2      3/4    7.5224  6.4137  9.6206
 4   2.4191(3) <<  Ca1    [                    =       04]       144.28 -18.23       1/2      3/4  0.58643    6.4137  9.6206  7.5224

Angles (Degrees)  At1...V...At2 with Vertex V =  F1
------------------------------------------------------------------------------------------------------------------------------------
```

```
 Li1    ,  Ca1_b     107.80(3)     Li1    ,  Ca1_a     107.80(3)     Li1    ,  Ca1       107.80(3)     Ca1_b  ,  Ca1_a     111.09(1)
 Ca1_b  ,  Ca1       111.09(1)     Ca1_a  ,  Ca1       111.09(1)

                                                                                                   1.1 Gpa    Page    8
=====================================================================================================================================
 2.8 Angstrom Coordination Sphere Around Atom I = F2     [ARU = 1555.03]         0.10462  0.39538  0.60462    1.3420  5.0717  7.7557
-------------------------------------------------------------------------------------------------------------------------------------
 Nr     d(I,J) To  Atom J  Symm_Oper. on Atom J    ARU(J)  Type     Phi     Mu         X        Y        Z         XO      YO      ZO
-------------------------------------------------------------------------------------------------------------------------------------
 1  1.8343(11) <<  Li2    [                    =       08]       135.00 -35.26   0.02206  0.47794  0.52206    0.2830  6.1307  6.6967
 2   2.3895(3) <<  Ca2_a  [1-z,1/2-x,y         =  6655.05]       -19.40 -34.17       1/4  0.34420      1/2    3.2069  4.4152  6.4137
 3   2.3895(3) <<  Ca2    [                    =       05]        63.93  51.30   0.15580      1/2      3/4    1.9986  6.4137  9.6206
 4   2.3895(3) <<  Ca2_b  [-1/2+y,-1/2+z,1/2+x = 21445.05]      -125.74  15.95         0      1/4  0.65580   -0.0000  3.2069  8.4123

Angles (Degrees)  At1...V...At2 with Vertex V =  F2
-------------------------------------------------------------------------------------------------------------------------------------
 Li2    ,  Ca2_a     106.56(3)     Li2    ,  Ca2       106.56(3)     Li2    ,  Ca2_b     106.56(3)     Ca2_a  ,  Ca2       112.22(1)
 Ca2_a  ,  Ca2_b     112.22(1)     Ca2    ,  Ca2_b     112.22(1)

                                                                                                   1.1 Gpa    Page    9
=====================================================================================================================================
 2.8 Angstrom Coordination Sphere Around Atom I = O1     [ARU = 1555.01]         0.43340  0.61696  0.70712    5.5594  7.9140  9.0705
-------------------------------------------------------------------------------------------------------------------------------------
 Nr     d(I,J) To  Atom J  Symm_Oper. on Atom J    ARU(J)  Type     Phi     Mu         X        Y        Z         XO      YO      ZO
-------------------------------------------------------------------------------------------------------------------------------------
 1   1.6326(3) --  Si1                                     Intra-157.12 -40.90   0.34477  0.57956  0.62379    4.4225  7.4343  8.0016



 2   1.6238(6) <<  Be1_b  [x,1-y,3/2-z         =  3566.06]       155.02  76.11   0.40586  0.62979  0.83001    5.2061  8.0786 10.6469
```

```
  3   2.4574(3) <<  Ca1    [                     =       04]        63.41 -39.05       1/2       3/4  0.58643    6.4137  9.6206  7.5224
  4   2.5311(3) <<  Ca1_c  [z,x,y                =  5555.04]       -37.39  12.55   0.58643       1/2       3/4    7.5224  6.4137  9.6206
  5   2.5527(4) <<  O2                                        Intra-162.92 -79.00   0.39710  0.60581  0.51177    5.0938  7.7710  6.5647
  6   2.6079(4) <<  O2_d   [z,1-x,3/2-y          =  7566.01]       -10.17  66.94   0.51177  0.60290  0.89419    6.5647  7.7337 11.4702
  7   2.6251(4) <<  O4                                        Intra 172.49 -16.29   0.23865  0.64264  0.64973    3.0613  8.2434  8.3344
  8   2.6996(4) <<  O4_e   [1-z,1/2+x,3/2-y      =  8656.01]       124.34  45.55   0.35027  0.73865  0.85736    4.4931  9.4750 10.9977
  9   2.7010(4) <<  O3_a   [x,1-y,3/2-z          =  3566.01]      -147.64  49.09   0.31693  0.54315  0.86626    4.0654  6.9672 11.1119
 10   2.7085(4) <<  O3                                        Intra-126.03 -20.34   0.31693  0.45685  0.63374    4.0654  5.8602  8.1293

Angles (Degrees)  At1...V...At2 with Vertex V =  O1
----------------------------------------------------------------------------------------------------------------------------------
 Si1    ,  Be1_b     120.92(2)     Si1    ,  Ca1         91.93(1)     Si1    ,  Ca1_c     120.54(1)     Si1    ,  O2          38.17(1)
 Si1    ,  O2_d      148.27(2)     Si1    ,  O4          35.96(1)     Si1    ,  O4_e      111.24(1)     Si1    ,  O3_a        90.38(1)
 Si1    ,  O3         33.44(1)     Be1_b  ,  Ca1        128.09(2)     Be1_b  ,  Ca1_c      91.02(2)     Be1_b  ,  O2         156.77(2)
 Be1_b  ,  O2_d       36.64(2)     Be1_b  ,  O4          93.01(2)     Be1_b  ,  O4_e       33.12(2)     Be1_b  ,  O3_a        35.07(2)
 Be1_b  ,  O3        107.11(2)     Ca1    ,  Ca1_c      106.20(1)     Ca1    ,  O2         58.93(1)     Ca1    ,  O2_d       119.58(1)
 Ca1    ,  O4         93.84(1)     Ca1    ,  O4_e       100.69(1)     Ca1    ,  O3_a      155.76(1)     Ca1    ,  O3         119.96(1)
 Ca1_c  ,  O2        108.76(1)     Ca1_c  ,  O2_d        57.32(1)     Ca1_c  ,  O4        150.85(1)     Ca1_c  ,  O4_e       119.60(1)
 Ca1_c  ,  O3_a       93.27(1)     Ca1_c  ,  O3          93.09(1)     O2     ,  O2_d      165.85(2)     O2     ,  O4          63.78(1)
 O2     ,  O4_e      131.39(1)     O2     ,  O3_a       128.42(1)     O2     ,  O3         61.04(1)     O2_d   ,  O4         129.31(1)
 O2_d   ,  O4_e       62.32(1)     O2_d   ,  O3_a        59.57(1)     O2_d   ,  O3        118.68(1)     O4     ,  O4_e        75.63(1)
 O4     ,  O3_a       74.31(1)     O4     ,  O3          58.18(1)     O4_e   ,  O3_a       56.27(1)     O4_e   ,  O3         117.95(1)
 O3_a   ,  O3         72.05(1)
```



```
==================================================================================================================================
 2.8 Angstrom Coordination Sphere Around Atom I = O2      [ARU = 1555.01]           0.39710  0.60581  0.51177    5.0938  7.7710  6.5647
----------------------------------------------------------------------------------------------------------------------------------
 Nr     d(I,J)  To  Atom J  Symm_Oper. on Atom J    ARU(J)  Type      Phi      Mu         X        Y        Z         XO       YO       ZO
```

No	Dist		Atom	Symm. op.			Ang1	Ang2	x	y	z	X	Y	Z
1	1.6213(3)	--	Si1			Intra	-153.36	62.41	0.34477	0.57956	0.62379	4.4225	7.4343	8.0016
2	1.6256(6)	<<	Be1_c	[y,z,x	= 9555.06]		112.73	-56.69	0.37021	0.66999	0.40586	4.7488	8.5942	5.2061
3	1.9885(11)	<<	Li2_a	[1-z,1/2-x,y	= 6655.08]		-57.70	-12.61	0.47794	0.47794	0.47794	6.1307	6.1307	6.1307
4	2.4658(3)	<<	Ca1	[	= 04]		54.49	22.85	1/2	3/4	0.58643	6.4137	9.6206	7.5224
5	2.5527(4)	<<	O1			Intra	17.07	79.00	0.43340	0.61696	0.70712	5.5594	7.9140	9.0705
6	2.6079(4)	<<	O1_d	[1-y,3/2-z,x	= 12665.01]		94.30	-22.67	0.38304	0.79288	0.43340	4.9134	10.1706	5.5594
7	2.6386(4)	<<	O3_b	[y,z,x	= 9555.01]		25.05	-71.30	0.45685	0.63374	0.31693	5.8602	8.1293	4.0654
8	2.6752(4)	<<	O3			Intra	-118.29	35.79	0.31693	0.45685	0.63374	4.0654	5.8602	8.1293
9	2.7361(4)	<<	O4			Intra	166.91	40.30	0.23865	0.64264	0.64973	3.0613	8.2434	8.3344
10	2.7472(4)	<<	O4_e	[1/2-x,y,1-z	= 16556.01]		164.82	-48.95	0.26135	0.64264	0.35027	3.3524	8.2434	4.4931

Angles (Degrees) At1...V...At2 with Vertex V = O2

At1	At2	Angle	At1	At2	Angle	At1	At2	Angle	At1	At2	Angle
Si1 ,	Be1_c	139.29(3)	Si1 ,	Li2_a	103.77(3)	Si1 ,	Ca1	91.90(1)	Si1 ,	O1	38.48(1)
Si1 ,	O1_d	120.27(2)	Si1 ,	O3_b	171.09(2)	Si1 ,	O3	34.33(1)	Si1 ,	O4	32.33(1)
Si1 ,	O4_e	116.20(2)	Be1_c ,	Li2_a	110.25(4)	Be1_c ,	Ca1	93.34(2)	Be1_c ,	O1	146.17(2)
Be1_c ,	O1_d	36.60(2)	Be1_c ,	O3_b	36.99(2)	Be1_c ,	O3	140.26(3)	Be1_c ,	O4	107.19(2)
Be1_c ,	O4_e	31.59(2)	Li2_a ,	Ca1	115.11(3)	Li2_a ,	O1	99.52(3)	Li2_a ,	O1_d	135.31(3)
Li2_a ,	O3_b	75.75(3)	Li2_a ,	O3	74.87(3)	Li2_a ,	O4	132.14(3)	Li2_a ,	O4_e	107.93(3)
Ca1 ,	O1	58.61(1)	Ca1 ,	O1_d	59.77(1)	Ca1 ,	O3_b	96.35(1)	Ca1 ,	O3	120.96(1)
Ca1 ,	O4	90.97(1)	Ca1 ,	O4_e	120.21(1)	O1 ,	O1_d	109.84(1)	O1 ,	O3_b	150.37(2)
O1 ,	O3	62.36(1)	O1 ,	O4	59.40(1)	O1 ,	O4_e	147.80(2)	O1_d ,	O3_b	61.97(1)
O1_d ,	O3	148.88(2)	O1_d ,	O4	92.24(1)	O1_d ,	O4_e	60.48(1)	O3_b ,	O3	139.70(1)
O3_b ,	O4	143.61(2)	O3_b ,	O4_e	56.39(1)	O3 ,	O4	57.28(1)	O3 ,	O4_e	108.68(1)
O4 ,	O4_e	89.27(1)									

==

2.8 Angstrom Coordination Sphere Around Atom I = O3 [ARU = 1555.01] 0.31693 0.45685 0.63374 4.0654 5.8602 8.1293

Nr	d(I,J)	To	Atom J	Symm_Oper. on Atom J		ARU(J)	Type	Phi	Mu	X	Y	Z	XO	YO	ZO
1	1.6191(3)	--	Si1				Intra	77.22	-4.52	0.34477	0.57956	0.62379	4.4225	7.4343	8.0016
2	1.6591(6)	<<	Be1	[	=	06]		-44.25	16.28	0.40586	0.37021	0.66999	5.2061	4.7488	8.5942
3	2.4017(3)	<<	Ca2_c	[1-z,1/2-x,y	=	6655.05]		-120.72	-45.59	1/4	0.34420	1/2	3.2069	4.4152	6.4137
4	2.5465(4)	<<	O4_d	[1-z,1/2-x,y	=	6655.01]		-80.32	2.57	0.35027	0.26135	0.64264	4.4931	3.3524	8.2434
5	2.5942(4)	<<	O4				Intra	112.85	4.53	0.23865	0.64264	0.64973	3.0613	8.2434	8.3344
6	2.6081(3)	<<	Ca2	[	=	05]		165.01	34.88	0.15580	1/2	3/4	1.9986	6.4137	9.6206
7	2.6386(4)	<<	O2_b	[z,x,y	=	5555.01]		-17.05	-7.80	0.51177	0.39710	0.60581	6.5647	5.0938	7.7710
8	2.6752(4)	<<	O2				Intra	61.71	-35.79	0.39710	0.60581	0.51177	5.0938	7.7710	6.5647
9	2.7010(4)	<<	O1_a	[x,1-y,3/2-z	=	3566.01]		-32.36	49.09	0.43340	0.38304	0.79288	5.5594	4.9134	10.1706
10	2.7085(4)	<<	O1				Intra	53.97	20.34	0.43340	0.61696	0.70712	5.5594	7.9140	9.0705

Angles (Degrees) At1...V...At2 with Vertex V = O3

Si1	, Be1	121.45(2)	Si1	, Ca2_c	127.40(1)	Si1	, O4_d	157.50(2)	Si1	, O4	36.73(1)
Si1	, Ca2	90.78(1)	Si1	, O2_b	93.60(1)	Si1	, O2	34.38(1)	Si1	, O1_a	106.17(1)
Si1	, O1	33.76(1)	Be1	, Ca2_c	92.46(2)	Be1	, O4_d	38.03(2)	Be1	, O4	149.24(2)
Be1	, Ca2	121.79(2)	Be1	, O2_b	36.12(2)	Be1	, O2	112.21(2)	Be1	, O1_a	34.22(2)
Be1	, O1	91.79(2)	Ca2_c	, O4_d	59.97(1)	Ca2_c	, O4	118.09(1)	Ca2_c	, Ca2	104.65(1)
Ca2_c	, O2_b	93.83(1)	Ca2_c	, O2	98.59(1)	Ca2_c	, O1_a	121.78(1)	Ca2_c	, O1	154.37(1)
O4_d	, O4	165.05(1)	O4_d	, Ca2	108.45(1)	O4_d	, O2_b	63.96(1)	O4_d	, O2	131.69(1)
O4_d	, O1_a	61.84(1)	O4_d	, O1	129.68(1)	O4	, Ca2	56.85(1)	O4	, O2_b	130.11(1)
O4	, O2	62.54(1)	O4	, O1_a	118.45(1)	O4	, O1	59.30(1)	Ca2	, O2_b	152.86(1)
Ca2	, O2	119.17(1)	Ca2	, O1_a	94.62(1)	Ca2	, O1	94.44(1)	O2_b	, O2	76.35(1)

O2_b	,	O1_a	58.46(1)	O2_b	,	O1	75.23(1)	O2	,	O1_a	118.67(1)
O2	,	O1	56.61(1)	O1_a	,	O1	72.43(1)				

2.8 Angstrom Coordination Sphere Around Atom I = O4 [ARU = 1555.01] 0.23865 0.64264 0.64973 3.0613 8.2434 8.3344

Nr	d(I,J)	To	Atom J	Symm_Oper. on Atom J	ARU(J)	Type	Phi	Mu	X	Y	Z	XO	YO	ZO
1	1.6181(3)	--	Si1			Intra	-30.73	-11.87	0.34477	0.57956	0.62379	4.4225	7.4343	8.0016
2	1.6067(6)	<<	Be1_e	[1/2-y,z,1-x	= 23556.06]		165.90	-26.35	0.12979	0.66999	0.59414	1.6649	8.5942	7.6213
3	1.9735(11)	<<	Li1_a	[1-x,3/2-y,z	= 2665.07]		76.12	31.38	0.27015	0.77015	0.72985	3.4653	9.8790	9.3621
4	2.4762(3)	<<	Ca2	[	= 05]		-120.15	31.29	0.15580	1/2	3/4	1.9986	6.4137	9.6206
5	2.5465(4)	<<	O3_d	[1/2-y,z,1-x	= 23556.01]		-177.39	9.67	0.04315	0.63374	0.68307	0.5535	8.1293	8.7620
6	2.5942(4)	<<	O3			Intra	-67.15	-4.53	0.31693	0.45685	0.63374	4.0654	5.8602	8.1293
7	2.6251(4)	<<	O1			Intra	-7.51	16.29	0.43340	0.61696	0.70712	5.5594	7.9140	9.0705
8	2.6996(4)	<<	O1_c	[-1/2+y,3/2-z,1-x	= 22466.01]		129.01	-23.27	0.11696	0.79288	0.56660	1.5003	10.1706	7.2680
9	2.7361(4)	<<	O2			Intra	-13.09	-40.30	0.39710	0.60581	0.51177	5.0938	7.7710	6.5647
10	2.7472(4)	<<	O2_b	[1/2-x,y,1-z	= 16556.01]		-164.82	-48.95	0.10290	0.60581	0.48823	1.3199	7.7710	6.2627

Angles (Degrees) At1...V...At2 with Vertex V = O4

Si1	,	Be1_e	138.51(3)	Si1	,	Li1_a	110.44(3)	Si1	,	Ca2	95.65(1)	Si1	,	O3_d	147.20(2)
Si1	,	O3	36.75(1)	Si1	,	O1	36.33(1)	Si1	,	O1_c	139.65(2)	Si1	,	O2	32.41(1)
Si1	,	O2_b	106.99(1)	Be1_e	,	Li1_a	103.19(4)	Be1_e	,	Ca2	91.08(2)	Be1_e	,	O3_d	39.51(2)
Be1_e	,	O3	120.13(2)	Be1_e	,	O1	168.22(2)	Be1_e	,	O1_c	33.52(2)	Be1_e	,	O2	113.35(2)
Be1_e	,	O2_b	32.00(2)	Li1_a	,	Ca2	115.45(3)	Li1_a	,	O3_d	98.70(3)	Li1_a	,	O3	136.33(3)

Li1_a	O1	76.30(3)	Li1_a	O1_c	74.48(3)	Li1_a	O2	109.14(3)	Li1_a	O2_b	131.68(3)
Ca2	O3_d	57.11(1)	Ca2	O3	61.86(1)	Ca2	O1	99.79(1)	Ca2	O1_c	118.98(1)
Ca2	O2	121.82(1)	Ca2	O2_b	89.58(1)	O3_d	O3	110.69(1)	O3_d	O1	152.18(1)
O3_d	O1_c	61.90(1)	O3_d	O2	146.35(1)	O3_d	O2_b	59.65(1)	O3	O1	62.52(1)
O3	O1_c	148.04(1)	O3	O2	60.18(1)	O3	O2_b	91.59(1)	O1	O1_c	138.64(1)
O1	O2	56.82(1)	O1	O2_b	142.48(1)	O1_c	O2	107.30(1)	O1_c	O2_b	57.21(1)
O2	O2_b	87.33(1)									

1.1 Gpa Page 13

2.8 Angstrom Coordination Sphere Around Atom I = Be1 [ARU = 1555.06] 0.40586 0.37021 0.66999 5.2061 4.7488 8.5942

Nr	d(I,J)	To	Atom J	Symm_Oper. on Atom J	ARU(J)	Type	Phi	Mu	X	Y	Z	XO	YO	ZO
1	1.6067(6)	<<	O4_c	[1-z,1/2-x,y	= 6655.01]		-117.05	-12.61	0.35027	0.26135	0.64264	4.4931	3.3524	8.2434
2	1.6238(6)	<<	O1_a	[x,1-y,3/2-z	= 3566.01]		24.98	76.11	0.43340	0.38304	0.79288	5.5594	4.9134	10.1706
3	1.6256(6)	<<	O2_b	[z,x,y	= 5555.01]		14.25	-30.43	0.51177	0.39710	0.60581	6.5647	5.0938	7.7710
4	1.6591(6)	<<	O3	[	= 01]		135.75	-16.28	0.31693	0.45685	0.63374	4.0654	5.8602	8.1293

Angles (Degrees) At1...V...At2 with Vertex V = Be1

O4_c	O1_a	113.37(3)	O4_c	O2_b	116.41(3)	O4_c	O3	102.46(3)	O1_a	O2_b	106.76(3)
O1_a	O3	110.72(3)	O2_b	O3	106.89(3)						

1.1 Gpa Page 14

2.8 Angstrom Coordination Sphere Around Atom I = Li1 [ARU = 1555.07] 0.72985 0.72985 0.72985 9.3621 9.3621 9.3621

Nr	d(I,J)	To	Atom J	Symm_Oper. on Atom J	ARU(J)	Type	Phi	Mu	X	Y	Z	XO	YO	ZO

1	1.8759(11)	<<	F1	[	= 02]	-135.00	-35.26	0.64542	0.64542	0.64542	8.2791	8.2791	8.2791
2	1.9735(11)	<<	O4_a	[1-x,3/2-y,z	= 2665.01]	76.12	-31.38	0.76135	0.85736	0.64973	9.7662	10.9977	8.3344
3	1.9735(11)	<<	O4_c	[3/2-y,z,1-x	= 23656.01]	-32.14	11.81	0.85736	0.64973	0.76135	10.9977	8.3344	9.7662
4	1.9735(11)	<<	O4_b	[z,1-x,3/2-y	= 7566.01]	158.54	55.97	0.64973	0.76135	0.85736	8.3344	9.7662	10.9977

Angles (Degrees) At1...V...At2 with Vertex V = Li1

F1	,	O4_a	107.22(5)	F1	,	O4_c	107.22(5)	F1	,	O4_b	107.22(5)
O4_a	,	O4_c	111.63(5)	O4_a	,	O4_b	111.63(5)	O4_c	,	O4_b	111.63(5)

2.8 Angstrom Coordination Sphere Around Atom I = Li2 [ARU = 1555.08] 0.02206 0.47794 0.52206 0.2830 6.1307 6.6967

Nr	d(I,J)	To	Atom J	Symm_Oper. on Atom J	ARU(J) Type	Phi	Mu	X	Y	Z	XO	YO	ZO
1	1.8343(11)	<<	F2	[	= 03]	-45.00	35.26	0.10462	0.39538	0.60462	1.3420	5.0717	7.7557
2	1.9885(11)	<<	O2_b	[1/2-z,x,1-y	= 20556.01]	-112.71	-55.58	-0.01177	0.39710	0.39419	-0.1510	5.0938	5.0564
3	1.9885(11)	<<	O2_c	[1/2-y,z,1-x	= 23556.01]	165.18	31.43	-0.10581	0.51177	0.60290	-1.3573	6.5647	7.7337
4	1.9885(11)	<<	O2_a	[1/2-x,y,1-z	= 16556.01]	57.70	-12.61	0.10290	0.60581	0.48823	1.3199	7.7710	6.2627

Angles (Degrees) At1...V...At2 with Vertex V = Li2

F2	,	O2_b	107.53(5)	F2	,	O2_c	107.53(5)	F2	,	O2_a	107.53(5)
O2_b	,	O2_c	111.34(5)	O2_b	,	O2_a	111.34(5)	O2_c	,	O2_a	111.34(5)

List of angles 1.9 GPa

2.8 Angstrom Coordination Sphere Around Atom I = Ca1 [ARU = 1555.04] 1/2 3/4 0.58646 6.4000 9.6000 7.5068

Nr	d(I,J)	To	Atom J	Symm_Oper. on Atom J		ARU(J)	Type	Phi	Mu	X	Y	Z	XO	YO	ZO
1	2.4136(2)	<<	F1	[	=	02]		-35.73	18.21	0.64540	0.64540	0.64540	8.2612	8.2612	8.2612
2	2.4136(2)	<<	F1_a	[1-x,3/2-y,z	=	2665.02]		144.27	18.21	0.35460	0.85460	0.64540	4.5388	10.9389	8.2612
3	2.4504(3)	<<	O1_b	[1-x,3/2-y,z	=	2665.01]		63.58	39.02	0.56618	0.88319	0.70700	7.2472	11.3049	9.0496
4	2.4504(3)	<<	O1	[	=	01]		-116.42	39.02	0.43382	0.61681	0.70700	5.5529	7.8952	9.0496
5	2.4668(3)	<<	O2_b	[1-x,3/2-y,z	=	2665.01]		54.56	-22.93	0.60293	0.89460	0.51138	7.7175	11.4509	6.5457
6	2.4668(3)	<<	O2	[	=	01]		-125.44	-22.93	0.39707	0.60540	0.51138	5.0825	7.7492	6.5457
7	2.5211(3)	<<	O1_c	[y,z,x	=	9555.01]		-20.21	-50.80	0.61681	0.70700	0.43382	7.8952	9.0496	5.5529
8	2.5211(3)	<<	O1_d	[1-y,3/2-z,x	=	12665.01]		159.79	-50.80	0.38319	0.79300	0.43382	4.9049	10.1505	5.5529

Angles (Degrees) At1...V...At2 with Vertex V = Ca1

F1	, F1_a	143.57(1)	F1	, O1_b	85.56(1)	F1	, O1	71.57(1)	F1	, O2_b	97.25(1)
F1	, O2	96.74(1)	F1	, O1_c	70.36(1)	F1	, O1_d	145.15(1)	F1_a	, O1_b	71.57(1)
F1_a	, O1	85.56(1)	F1_a	, O2_b	96.74(1)	F1_a	, O2	97.25(1)	F1_a	, O1_c	145.15(1)
F1_a	, O1_d	70.36(1)	O1_b	, O1	101.95(1)	O1_b	, O2_b	62.53(1)	O1_b	, O2	162.17(1)
O1_b	, O1_c	115.78(1)	O1_b	, O1_d	122.76(1)	O1	, O2_b	162.17(1)	O1	, O2	62.53(1)
O1	, O1_c	122.76(1)	O1	, O1_d	115.78(1)	O2_b	, O2	134.14(1)	O2_b	, O1_c	62.94(1)
O2_b	, O1_d	81.43(1)	O2	, O1_c	81.43(1)	O2	, O1_d	62.94(1)	O1_c	, O1_d	78.39(1)

2.8 Angstrom Coordination Sphere Around Atom I = Ca2 [ARU = 1555.05] 0.15594 1/2 3/4 1.9960 6.4000 9.6000

Nr	d(I,J)	To	Atom J	Symm_Oper. on Atom J		ARU(J)	Type	Phi	Mu	X	Y	Z	XO	YO	ZO
1	2.3842(2)	<<	F2	[	=	03]		-116.05	-51.25	0.10473	0.39527	0.60473	1.3406	5.0594	7.7406
2	2.3842(2)	<<	F2_a	[x,1-y,3/2-z	=	3566.03]		116.05	51.25	0.10473	0.60473	0.89527	1.3406	7.7406	11.4595
3	2.3970(2)	<<	O3_d	[1/2-y,1-z,1/2+x	=	24565.01]		-130.03	20.77	0.04333	0.36593	0.81642	0.5546	4.6839	10.4502
4	2.3970(2)	<<	O3_c	[1/2-y,z,1-x	=	23556.01]		130.03	-20.77	0.04333	0.63407	0.68358	0.5546	8.1161	8.7499
5	2.4757(3)	<<	O4_b	[x,1-y,3/2-z	=	3566.01]		-59.90	31.22	0.23888	0.35689	0.85025	3.0577	4.5682	10.8833
6	2.4757(3)	<<	O4	[	=	01]		59.90	-31.22	0.23888	0.64311	0.64975	3.0577	8.2318	8.3168
7	2.5941(3)	<<	O3	[	=	01]		-15.11	-34.89	0.31642	0.45667	0.63407	4.0502	5.8454	8.1161
8	2.5941(3)	<<	O3_b	[x,1-y,3/2-z	=	3566.01]		15.11	34.89	0.31642	0.54333	0.86593	4.0502	6.9546	11.0839

Angles (Degrees) At1...V...At2 with Vertex V = Ca2

F2	, F2_a	148.09(1)	F2	, O3_d	73.07(1)	F2	, O3_c	87.74(1)	F2	, O4_b	96.09(1)
F2	, O4	97.45(1)	F2	, O3	69.60(1)	F2	, O3_b	141.63(1)	F2_a	, O3_d	87.74(1)
F2_a	, O3_c	73.07(1)	F2_a	, O4_b	97.45(1)	F2_a	, O4	96.09(1)	F2_a	, O3	141.63(1)
F2_a	, O3_b	69.60(1)	O3_d	, O3_c	106.07(1)	O3_d	, O4_b	62.89(1)	O3_d	, O4	166.27(1)
O3_d	, O3	121.74(1)	O3_d	, O3_b	115.24(1)	O3_c	, O4_b	166.27(1)	O3_c	, O4	62.89(1)
O3_c	, O3	115.24(1)	O3_c	, O3_b	121.74(1)	O4_b	, O4	129.21(1)	O4_b	, O3	78.39(1)
O4_b	, O3_b	61.45(1)	O4	, O3	61.45(1)	O4	, O3_b	78.39(1)	O3	, O3_b	75.28(1)

```
 2.8 Angstrom Coordination Sphere Around Atom I = Si1    [ARU = 1555.01]         0.34486  0.57947  0.62375    4.4142  7.4172  7.9840
------------------------------------------------------------------------------------------------------------------------------------
 Nr     d(I,J) To  Atom J  Symm_Oper. on Atom J    ARU(J)  Type      Phi      Mu          X         Y         Z         XO      YO      ZO
------------------------------------------------------------------------------------------------------------------------------------
 1   1.6169(3) --  O4                                      Intra 149.01  11.88   0.23888  0.64311  0.64975    3.0577  8.2318  8.3168
 2   1.6188(2) --  O3                                      Intra-103.04   4.68   0.31642  0.45667  0.63407    4.0502  5.8454  8.1161
 3   1.6204(3) --  O2                                      Intra  26.41 -62.58   0.39707  0.60540  0.51138    5.0825  7.7492  6.5457
 4   1.6312(3) --  O1                                      Intra  22.77  40.79   0.43382  0.61681  0.70700    5.5529  7.8952  9.0496

Tetrahedral Volume  2.173 Ang**3, Quadratic Elongation  1.005, Angle Variance  19.29 Deg**2 [K.Robinson +, Science 1971,172,567-570]

Tau(4)-Descriptor for 4-Coordination (L.Yang, D.R.Powell, R.P.Houser, Dalton Trans. (2007), 955-964)
------------------------------------------------------------------------------------------------------------------------------------
Tau(4) = (360 - (Beta + Alpha)) / 141 = 0.94 :: (Extreme Forms: 0.00 for SQP and 1.00 for TET; 0.85 for TRP)

Angles (Degrees)  At1...V...At2 with Vertex V =  Si1
------------------------------------------------------------------------------------------------------------------------------------
 O4     ,  O3        106.48(1)     O4     ,  O2        115.19(1)     O4     ,  O1        107.67(1)     O3     ,  O2        111.35(1)
 O3     ,  O1        112.84(1)     O2     ,  O1        103.41(1)
                                                                                                  1.9 GPa    Page    7
====================================================================================================================================
 2.8 Angstrom Coordination Sphere Around Atom I = F1     [ARU = 1555.02]         0.64540  0.64540  0.64540    8.2612  8.2612  8.2612
------------------------------------------------------------------------------------------------------------------------------------
 Nr     d(I,J) To  Atom J  Symm_Oper. on Atom J    ARU(J)  Type      Phi      Mu          X         Y         Z         XO      YO      ZO
------------------------------------------------------------------------------------------------------------------------------------
 1   1.8715(9) <<  Li1    [                    =       07]         45.00  35.26   0.72982  0.72982  0.72982    9.3417  9.3417  9.3417
 2   2.4136(2) <<  Ca1_b  [y,z,x               =  9555.04]        -29.40 -50.45       3/4  0.58646       1/2    9.6000  7.5068  6.4000
 3   2.4136(2) <<  Ca1_a  [z,x,y               =  5555.04]       -112.07  33.69   0.58646       1/2       3/4    7.5068  6.4000  9.6000
 4   2.4136(2) <<  Ca1    [                    =       04]        144.27 -18.21       1/2       3/4  0.58646    6.4000  9.6000  7.5068

Angles (Degrees)  At1...V...At2 with Vertex V =  F1
------------------------------------------------------------------------------------------------------------------------------------
 Li1    ,  Ca1_b     107.78(3)     Li1    ,  Ca1_a     107.78(3)     Li1    ,  Ca1       107.78(3)     Ca1_b  ,  Ca1_a     111.11(1)
 Ca1_b  ,  Ca1       111.11(1)     Ca1_a  ,  Ca1       111.11(1)
                                                                                                  1.9 GPa    Page    8
====================================================================================================================================
 2.8 Angstrom Coordination Sphere Around Atom I = F2     [ARU = 1555.03]         0.10473  0.39527  0.60473    1.3406  5.0594  7.7406
------------------------------------------------------------------------------------------------------------------------------------
 Nr     d(I,J) To  Atom J  Symm_Oper. on Atom J    ARU(J)  Type      Phi      Mu          X         Y         Z         XO      YO      ZO
------------------------------------------------------------------------------------------------------------------------------------
 1   1.8340(9) << <Li2    [                    =       08]        135.00 -35.26   0.02201  0.47799  0.52201    0.2817  6.1183  6.6818
 2   2.3842(2) <<  Ca2    [                    =       05]         63.95  51.25   0.15594       1/2       3/4    1.9960  6.4000  9.6000
 3   2.3842(2) <<  Ca2_b  [-1/2+y,-1/2+z,1/2+x = 21445.05]       -125.79  15.96         0       1/4  0.65594   -0.0000  3.2000  8.3961
 4   2.3842(2) <<  Ca2_a  [1-z,1/2-x,y         =  6655.05]        -19.42 -34.21       1/4  0.34406       1/2    3.2000  4.4040  6.4000

Angles (Degrees)  At1...V...At2 with Vertex V =  F2
------------------------------------------------------------------------------------------------------------------------------------
<Li2    ,  Ca2       106.52(3)    <Li2    ,  Ca2_b     106.52(3)    <Li2    ,  Ca2_a     106.52(3)     Ca2    ,  Ca2_b     112.25(1)
 Ca2    ,  Ca2_a     112.25(1)     Ca2_b  ,  Ca2_a     112.25(1)
                                                                                                  1.9 GPa    Page    9
====================================================================================================================================
```

2.8 Angstrom Coordination Sphere Around Atom I = O1 [ARU = 1555.01] 0.43382 0.61681 0.70700 5.5529 7.8952 9.0496

Nr	d(I,J)	To	Atom J	Symm_Oper. on Atom J	ARU(J)	Type	Phi	Mu	X	Y	Z	XO	YO	ZO
1	1.6312(3)	--	Si1			Intra	-157.23	-40.79	0.34486	0.57947	0.62375	4.4142	7.4172	7.9840
2	1.6216(6)	<<	Be1_b	[x,1-y,3/2-z	= 3566.06]		155.20	75.90	0.40581	0.62975	0.82987	5.1944	8.0608	10.6224
3	2.4504(3)	<<	Ca1	[	= 04]		63.58	-39.02	1/2	3/4	0.58646	6.4000	9.6000	7.5068
4	2.5211(3)	<<	Ca1_c	[z,x,y	= 5555.04]		-37.42	12.61	0.58646	1/2	3/4	7.5068	6.4000	9.6000
5	2.5519(3)	<<	O2			Intra	-162.75	-78.87	0.39707	0.60540	0.51138	5.0825	7.7492	6.5457
6	2.6045(3)	<<	O2_d	[z,1-x,3/2-y	= 7566.01]		-10.15	67.22	0.51138	0.60293	0.89460	6.5457	7.7175	11.4509
7	2.6223(3)	<<	O4			Intra	172.32	-16.23	0.23888	0.64311	0.64975	3.0577	8.2318	8.3168
8	2.6957(3)	<<	O4_e	[1-z,1/2+x,3/2-y	= 8656.01]		124.39	45.38	0.35025	0.73888	0.85689	4.4832	9.4577	10.9682
9	2.6984(3)	<<	O3_a	[x,1-y,3/2-z	= 3566.01]		-147.96	48.93	0.31642	0.54333	0.86593	4.0502	6.9546	11.0839
10	2.7076(3)	<<	O3			Intra	-126.25	-20.17	0.31642	0.45667	0.63407	4.0502	5.8454	8.1161

Angles (Degrees) At1...V...At2 with Vertex V = O1

Si1 , Be1_b	120.61(2)	Si1 , Ca1	91.94(1)	Si1 , Ca1_c	120.66(1)	Si1 , O2	38.15(1)
Si1 , O2_d	148.04(1)	Si1 , O4	35.98(1)	Si1 , O4_e	110.97(1)	Si1 , O3_a	90.09(1)
Si1 , O3	33.44(1)	Be1_b , Ca1	128.03(2)	Be1_b , Ca1_c	91.16(2)	Be1_b , O2	156.46(2)
Be1_b , O2_d	36.58(2)	Be1_b , O4	92.73(2)	Be1_b , O4_e	33.15(2)	Be1_b , O3_a	35.04(2)
Be1_b , O3	106.80(2)	Ca1 , Ca1_c	106.39(1)	Ca1 , O2	59.05(1)	Ca1 , O2_d	119.75(1)
Ca1 , O4	93.65(1)	Ca1 , O4_e	100.49(1)	Ca1 , O3_a	155.47(1)	Ca1 , O3	120.10(1)
Ca1_c , O2	108.85(1)	Ca1_c , O2_d	57.51(1)	Ca1_c , O4	151.00(1)	Ca1_c , O4_e	119.73(1)
Ca1_c , O3_a	93.46(1)	Ca1_c , O3	93.23(1)	O2 , O2_d	166.17(1)	O2 , O4	63.75(1)
O2 , O4_e	131.18(1)	O2 , O3_a	128.11(1)	O2 , O3	61.06(1)	O2_d , O4	128.98(1)
O2_d , O4_e	62.26(1)	O2_d , O3_a	59.56(1)	O2_d , O3	118.54(1)	O4 , O4_e	75.34(1)
O4 , O3_a	74.07(1)	O4 , O3	58.18(1)	O4_e , O3_a	56.25(1)	O4_e , O3	117.64(1)
O3_a , O3	71.76(1)						

2.8 Angstrom Coordination Sphere Around Atom I = O2 [ARU = 1555.01] 0.39707 0.60540 0.51138 5.0825 7.7492 6.5457

Nr	d(I,J)	To	Atom J	Symm_Oper. on Atom J	ARU(J)	Type	Phi	Mu	X	Y	Z	XO	YO	ZO
1	1.6204(3)	--	Si1			Intra	-153.59	62.58	0.34486	0.57947	0.62375	4.4142	7.4172	7.9840
2	1.6218(5)	<<	Be1_c	[y,z,x	= 9555.06]		112.51	-56.43	0.37025	0.67013	0.40581	4.7392	8.5777	5.1944
3	1.9787(9)	<<	<Li2_a	[1-z,1/2-x,y	= 6655.08]		-57.58	-12.47	0.47799	0.47799	0.47799	6.1183	6.1183	6.1183
4	2.4668(3)	<<	Ca1	[	= 04]		54.56	22.93	1/2	3/4	0.58646	6.4000	9.6000	7.5068
5	2.5519(3)	<<	O1			Intra	17.25	78.87	0.43382	0.61681	0.70700	5.5529	7.8952	9.0496
6	2.6045(3)	<<	O1_d	[1-y,3/2-z,x	= 12665.01]		94.23	-22.41	0.38319	0.79300	0.43382	4.9049	10.1505	5.5529
7	2.6352(4)	<<	O3_b	[y,z,x	= 9555.01]		25.69	-71.26	0.45667	0.63407	0.31642	5.8454	8.1161	4.0502
8	2.6751(3)	<<	O3			Intra	-118.47	35.95	0.31642	0.45667	0.63407	4.0502	5.8454	8.1161
9	2.7331(4)	<<	O4			Intra	166.59	40.39	0.23888	0.64311	0.64975	3.0577	8.2318	8.3168
10	2.7413(4)	<<	O4_e	[1/2-x,y,1-z	= 16556.01]		164.50	-48.79	0.26112	0.64311	0.35025	3.3423	8.2318	4.4832

Angles (Degrees) At1...V...At2 with Vertex V = O2

Si1	, Be1_c	139.19(2)	Si1	, <Li2_a	103.82(3)	Si1	, Ca1	91.61(1)	Si1	, O1	38.45(1)
Si1	, O1_d	119.94(1)	Si1	, O3_b	171.31(2)	Si1	, O3	34.30(1)	Si1	, O4	32.37(1)
Si1	, O4_e	116.24(1)	Be1_c	, <Li2_a	110.61(3)	Be1_c	, Ca1	93.12(2)	Be1_c	, O1	145.82(2)
Be1_c	, O1_d	36.58(2)	Be1_c	, O3_b	36.98(2)	Be1_c	, O3	140.45(2)	Be1_c	, O4	107.03(2)
Be1_c	, O4_e	31.66(2)	<Li2_a	, Ca1	115.02(3)	<Li2_a	, O1	99.36(3)	<Li2_a	, O1_d	135.50(3)
<Li2_a	, O3_b	76.04(3)	<Li2_a	, O3	75.06(3)	<Li2_a	, O4	132.33(3)	<Li2_a	, O4_e	108.36(3)
Ca1	, O1	58.42(1)	Ca1	, O1_d	59.55(1)	Ca1	, O3_b	96.31(1)	Ca1	, O3	120.75(1)
Ca1	, O4	90.61(1)	Ca1	, O4_e	120.00(1)	O1	, O1_d	109.50(1)	O1	, O3_b	150.21(1)
O1	, O3	62.34(1)	O1	, O4	59.38(1)	O1	, O4_e	147.69(1)	O1_d	, O3_b	61.99(1)
O1_d	, O3	148.60(1)	O1_d	, O4	91.93(1)	O1_d	, O4_e	60.50(1)	O3_b	, O3	140.07(1)
O3_b	, O4	143.47(1)	O3_b	, O4_e	56.41(1)	O3	, O4	57.27(1)	O3	, O4_e	108.79(1)
O4	, O4_e	89.21(1)									

2.8 Angstrom Coordination Sphere Around Atom I = O3 [ARU = 1555.01] 0.31642 0.45667 0.63407 4.0502 5.8454 8.1161

Nr	d(I,J)	To	Atom J	Symm_Oper. on Atom J	ARU(J)	Type	Phi	Mu	X	Y	Z	XO	YO	ZO
1	1.6188(2)	--	Si1			Intra	76.96	-4.68	0.34486	0.57947	0.62375	4.4142	7.4172	7.9840
2	1.6571(5)	<<	Be1	[	= 06]		-44.03	16.17	0.40581	0.37025	0.67013	5.1944	4.7392	8.5777
3	2.3970(2)	<<	Ca2_c	[1-z,1/2-x,y	= 6655.05]		-120.53	-45.72	1/4	0.34406	1/2	3.2000	4.4040	6.4000
4	2.5429(3)	<<	O4_d	[1-z,1/2-x,y	= 6655.01]		-80.19	2.61	0.35025	0.26112	0.64311	4.4832	3.3423	8.2318
5	2.5924(3)	<<	O4			Intra	112.58	4.44	0.23888	0.64311	0.64975	3.0577	8.2318	8.3168
6	2.5941(3)	<<	Ca2	[	= 05]		164.89	34.89	0.15594	1/2	3/4	1.9960	6.4000	9.6000
7	2.6352(4)	<<	O2_b	[z,x,y	= 5555.01]		-17.00	-8.01	0.51138	0.39707	0.60540	6.5457	5.0825	7.7492
8	2.6751(3)	<<	O2			Intra	61.53	-35.95	0.39707	0.60540	0.51138	5.0825	7.7492	6.5457
9	2.6984(3)	<<	O1_a	[x,1-y,3/2-z	= 3566.01]		-32.04	48.93	0.43382	0.38319	0.79300	5.5529	4.9049	10.1505
10	2.7076(3)	<<	O1			Intra	53.75	20.17	0.43382	0.61681	0.70700	5.5529	7.8952	9.0496

Angles (Degrees) At1...V...At2 with Vertex V = O3

Si1	, Be1	121.04(2)	Si1	, Ca2_c	127.24(1)	Si1	, O4_d	157.10(2)	Si1	, O4	36.73(1)
Si1	, Ca2	90.98(1)	Si1	, O2_b	93.26(1)	Si1	, O2	34.34(1)	Si1	, O1_a	105.95(1)
Si1	, O1	33.73(1)	Be1	, Ca2_c	92.46(2)	Be1	, O4_d	38.06(2)	Be1	, O4	149.02(2)
Be1	, Ca2	122.02(2)	Be1	, O2_b	36.06(2)	Be1	, O2	111.85(2)	Be1	, O1_a	34.19(2)
Be1	, O1	91.50(2)	Ca2_c	, O4_d	60.07(1)	Ca2_c	, O4	118.24(1)	Ca2_c	, Ca2	104.91(1)
Ca2_c	, O2_b	93.56(1)	Ca2_c	, O2	98.31(1)	Ca2_c	, O1_a	121.85(1)	Ca2_c	, O1	154.02(1)
O4_d	, O4	165.43(1)	O4_d	, Ca2	108.62(1)	O4_d	, O2_b	63.90(1)	O4_d	, O2	131.41(1)
O4_d	, O1_a	61.82(1)	O4_d	, O1	129.42(1)	O4	, Ca2	57.03(1)	O4	, O2_b	129.78(1)
O4	, O2	62.49(1)	O4	, O1_a	118.41(1)	O4	, O1	59.26(1)	Ca2	, O2_b	153.06(1)
Ca2	, O2	119.29(1)	Ca2	, O1_a	94.83(1)	Ca2	, O1	94.61(1)	O2_b	, O2	76.04(1)
O2_b	, O1_a	58.45(1)	O2_b	, O1	75.03(1)	O2	, O1_a	118.41(1)	O2	, O1	56.60(1)
O1_a	, O1	72.24(1)									

2.8 Angstrom Coordination Sphere Around Atom I = O4 [ARU = 1555.01] 0.23888 0.64311 0.64975 3.0577 8.2318 8.3168

Nr	d(I,J)	To	Atom J	Symm_Oper. on Atom J	ARU(J)	Type	Phi	Mu	X	Y	Z	XO	YO	ZO
1	1.6169(3)	--	Si1			Intra	-30.99	-11.88	0.34486	0.57947	0.62375	4.4142	7.4172	7.9840

```
  2   1.6052(5) <<  Be1_e  [1/2-y,z,1-x         = 23556.06]       166.09 -26.30   0.12975  0.67013  0.59419    1.6608  8.5777  7.6057
  3   1.9638(9) <<  Li1_a  [1-x,3/2-y,z         =  2665.07]        76.16  31.46   0.27018  0.77018  0.72982    3.4583  9.8583  9.3417
  4   2.4757(3) <<  Ca2    [                    =       05]      -120.10  31.22   0.15594      1/2      3/4    1.9960  6.4000  9.6000
  5   2.5429(3) <<  O3_d   [1/2-y,z,1-x         = 23556.01]      -177.35   9.80   0.04333  0.63407  0.68358    0.5546  8.1161  8.7499
  6   2.5924(3) <<  O3                                    Intra -67.42  -4.44   0.31642  0.45667  0.63407    4.0502  5.8454  8.1161
  7   2.6223(3) <<  O1                                    Intra  -7.68  16.23   0.43382  0.61681  0.70700    5.5529  7.8952  9.0496
  8   2.6957(3) <<  O1_c   [-1/2+y,3/2-z,1-x    = 22466.01]       129.16 -23.38   0.11681  0.79300  0.56618    1.4952 10.1505  7.2472
  9   2.7331(4) <<  O2                                    Intra -13.41 -40.39   0.39707  0.60540  0.51138    5.0825  7.7492  6.5457
 10   2.7413(4) <<  O2_b   [1/2-x,y,1-z         = 16556.01]      -164.50 -48.79   0.10293  0.60540  0.48862    1.3175  7.7492  6.2544

Angles (Degrees)  At1...V...At2 with Vertex V =  O4
-----------------------------------------------------------------------------------------------------------------------------------
 Si1     ,  Be1_e     138.37(2)     Si1     ,  Li1_a     110.71(3)     Si1     ,  Ca2        95.38(1)     Si1     ,  O3_d      146.92(2)
 Si1     ,  O3         36.78(1)     Si1     ,  O1         36.35(1)     Si1     ,  O1_c      139.74(1)     Si1     ,  O2         32.45(1)
 Si1     ,  O2_b      106.80(1)     Be1_e   ,  Li1_a     103.32(3)     Be1_e   ,  Ca2        90.91(2)     Be1_e   ,  O3_d       39.53(2)
 Be1_e   ,  O3        119.82(2)     Be1_e   ,  O1        168.38(2)     Be1_e   ,  O1_c       33.53(2)     Be1_e   ,  O2        113.31(2)
 Be1_e   ,  O2_b       32.03(2)     Li1_a   ,  Ca2       115.46(3)     Li1_a   ,  O3_d       98.61(3)     Li1_a   ,  O3        136.45(3)
 Li1_a   ,  O1         76.49(3)     Li1_a   ,  O1_c       74.68(3)     Li1_a   ,  O2        109.47(3)     Li1_a   ,  O2_b      131.91(3)
 Ca2     ,  O3_d       57.04(1)     Ca2     ,  O3         61.52(1)     Ca2     ,  O1         99.68(1)     Ca2     ,  O1_c      118.93(1)
 Ca2     ,  O2        121.53(1)     Ca2     ,  O2_b       89.28(1)     O3_d    ,  O3        110.38(1)     O3_d    ,  O1        152.03(1)
 O3_d    ,  O1_c       61.93(1)     O3_d    ,  O2        146.26(1)     O3_d    ,  O2_b       59.69(1)     O3      ,  O1         62.56(1)
 O3      ,  O1_c      147.82(1)     O3      ,  O2         60.24(1)     O3      ,  O2_b       91.30(1)     O1      ,  O1_c      138.92(1)
 O1      ,  O2         56.87(1)     O1      ,  O2_b      142.34(1)     O1_c    ,  O2        107.34(1)     O1_c    ,  O2_b       57.24(1)
 O2      ,  O2_b       87.23(1)
```



```
===================================================================================================================================
 2.8 Angstrom Coordination Sphere Around Atom I = Be1    [ARU = 1555.06]         0.40581  0.37025  0.67013    5.1944  4.7392  8.5777
-----------------------------------------------------------------------------------------------------------------------------------
 Nr     d(I,J) To  Atom J  Symm_Oper. on Atom J    ARU(J)  Type      Phi      Mu         X         Y         Z        XO      YO      ZO
-----------------------------------------------------------------------------------------------------------------------------------
  1   1.6052(5) <<  O4_c   [1-z,1/2-x,y         =  6655.01]      -116.98 -12.44   0.35025  0.26112  0.64311    4.4832  3.3423  8.2318
  2   1.6216(6) <<  O1_a   [x,1-y,3/2-z         =  3566.01]        24.80  75.90   0.43382  0.38319  0.79300    5.5529  4.9049 10.1505
  3   1.6218(5) <<  O2_b   [z,x,y               =  5555.01]        14.25 -30.72   0.51138  0.39707  0.60540    6.5457  5.0825  7.7492
  4   1.6571(5) <<  O3     [                    =       01]       135.97 -16.17   0.31642  0.45667  0.63407    4.0502  5.8454  8.1161

Angles (Degrees)  At1...V...At2 with Vertex V =  Be1
-----------------------------------------------------------------------------------------------------------------------------------
 O4_c    ,  O1_a      113.32(3)     O4_c    ,  O2_b      116.31(3)     O4_c    ,  O3        102.42(3)     O1_a    ,  O2_b      106.84(3)
 O1_a    ,  O3        110.77(3)     O2_b    ,  O3        106.96(3)
```



```
===================================================================================================================================
 2.8 Angstrom Coordination Sphere Around Atom I = Li1    [ARU = 1555.07]         0.72982  0.72982  0.72982    9.3417  9.3417  9.3417
-----------------------------------------------------------------------------------------------------------------------------------
 Nr     d(I,J) To  Atom J  Symm_Oper. on Atom J    ARU(J)  Type      Phi      Mu         X         Y         Z        XO      YO      ZO
-----------------------------------------------------------------------------------------------------------------------------------
  1   1.8715(9) <<  F1     [                    =       02]      -135.00 -35.26   0.64540  0.64540  0.64540    8.2612  8.2612  8.2612
  2   1.9638(9) <<  O4_a   [1-x,3/2-y,z         =  2665.01]        76.16 -31.46   0.76112  0.85689  0.64975    9.7424 10.9682  8.3168
  3   1.9638(9) <<  O4_c   [3/2-y,z,1-x         = 23656.01]       -32.22  11.77   0.85689  0.64975  0.76112   10.9682  8.3168  9.7424
  4   1.9638(9) <<  O4_b   [z,1-x,3/2-y         =  7566.01]       158.65  55.92   0.64975  0.76112  0.85689    8.3168  9.7424 10.9682
```

Angles (Degrees) At1...V...At2 with Vertex V = Li1

F1	,	O4_a	107.14(4)	F1	,	O4_c	107.14(4)	F1	,	O4_b	107.14(4)
O4_a	,	O4_c	111.70(4)	O4_a	,	O4_b	111.70(4)	O4_c	,	O4_b	111.70(4)

2.8 Angstrom Coordination Sphere Around Atom I = Li2 [ARU = 1555.08] 0.02201 0.47799 0.52201 0.2817 6.1183 6.6818

Nr	d(I,J)	To	Atom J	Symm_Oper. on Atom J	ARU(J)	Type	Phi	Mu	X	Y	Z	XO	YO	ZO
1	1.8340(9)	<<	F2	[	= 03]		-45.00	35.26	0.10473	0.39527	0.60473	1.3406	5.0594	7.7406
2	1.9787(9)	<<	O2_a	[1/2-x,y,1-z	= 16556.01]		57.58	-12.47	0.10293	0.60540	0.48862	1.3175	7.7492	6.2544
3	1.9787(9)	<<	O2_b	[1/2-z,x,1-y	= 20556.01]		-112.42	-55.51	-0.01138	0.39707	0.39460	-0.1456	5.0825	5.0509
4	1.9787(9)	<<	O2_c	[1/2-y,z,1-x	= 23556.01]		165.32	31.57	-0.10540	0.51138	0.60293	-1.3491	6.5457	7.7175

Angles (Degrees) At1...V...At2 with Vertex V = <Li2

F2	,	O2_a	107.36(4)	F2	,	O2_b	107.36(4)	F2	,	O2_c	107.36(4)
O2_a	,	O2_b	111.50(4)	O2_a	,	O2_c	111.50(4)	O2_b	,	O2_c	111.50(4)

List of angles 4.2 GPa

2.8 Angstrom Coordination Sphere Around Atom I = Ca1 [ARU = 1555.04] 1/2 3/4 0.58662 6.3587 9.5380 7.4602

Nr	d(I,J)	To	Atom J	Symm_Oper. on Atom J	ARU(J)	Type	Phi	Mu	X	Y	Z	XO	YO	ZO
1	2.3979(4)	<<	F1	[	= 02]		-35.70	18.19	0.64547	0.64547	0.64547	8.2087	8.2087	8.2087
2	2.3979(4)	<<	F1_a	[1-x,3/2-y,z	= 2665.02]		144.30	18.19	0.35453	0.85453	0.64547	4.5087	10.8673	8.2087
3	2.4291(4)	<<	O1	[	= 01]		-115.92	38.99	0.43511	0.61648	0.70680	5.5334	7.8400	8.9886
4	2.4291(4)	<<	O1_b	[1-x,3/2-y,z	= 2665.01]		64.08	38.99	0.56489	0.88352	0.70680	7.1839	11.2360	8.9886
5	2.4638(4)	<<	O2	[	= 01]		-125.17	-23.12	0.39738	0.60434	0.51055	5.0536	7.6856	6.4928
6	2.4638(4)	<<	O2_b	[1-x,3/2-y,z	= 2665.01]		54.83	-23.12	0.60262	0.89566	0.51055	7.6637	11.3904	6.4928
7	2.4917(4)	<<	O1_c	[y,z,x	= 9555.01]		-20.35	-50.65	0.61648	0.70680	0.43511	7.8400	8.9886	5.5334
8	2.4917(4)	<<	O1_d	[1-y,3/2-z,x	= 12665.01]		159.65	-50.65	0.38352	0.79320	0.43511	4.8773	10.0874	5.5334

Angles (Degrees) At1...V...At2 with Vertex V = Ca1

F1	F1_a	143.62(1)	F1	O1	71.23(1)	F1	O1_b	85.93(1)	F1	O2	96.57(1)
F1	O2_b	97.51(1)	F1	O1_c	70.15(1)	F1	O1_d	145.31(1)	F1_a	O1	85.93(1)
F1_a	O1_b	71.23(1)	F1_a	O2	97.51(1)	F1_a	O2_b	96.57(1)	F1_a	O1_c	145.31(1)
F1_a	O1_d	70.15(1)	O1	O1_b	102.01(1)	O1	O2	62.71(1)	O1	O2_b	162.28(1)
O1	O1_c	122.30(1)	O1	O1_d	116.02(1)	O1_b	O2	162.28(1)	O1_b	O2_b	62.71(1)
O1_b	O1_c	116.02(1)	O1_b	O1_d	122.30(1)	O2	O2_b	133.76(1)	O2	O1_c	81.11(1)
O2	O1_d	63.08(1)	O2_b	O1_c	63.08(1)	O2_b	O1_d	81.11(1)	O1_c	O1_d	78.70(1)

2.8 Angstrom Coordination Sphere Around Atom I = Ca2 [ARU = 1555.05] 0.15617 1/2 3/4 1.9860 6.3587 9.5380

Nr	d(I,J)	To	Atom J	Symm_Oper. on Atom J	ARU(J)	Type	Phi	Mu	X	Y	Z	XO	YO	ZO
1	2.3693(4)	<<	F2	[	= 03]		-116.12	-51.21	0.10478	0.39522	0.60478	1.3325	5.0261	7.6912
2	2.3693(4)	<<	F2_a	[x,1-y,3/2-z	= 3566.03]		116.12	51.21	0.10478	0.60478	0.89522	1.3325	7.6912	11.3848
3	2.3826(4)	<<	O3_d	[1/2-y,1-z,1/2+x	= 24565.01]		-129.85	20.40	0.04365	0.36518	0.81529	0.5551	4.6441	10.3683
4	2.3826(4)	<<	O3_c	[1/2-y,z,1-x	= 23556.01]		129.85	-20.40	0.04365	0.63482	0.68471	0.5551	8.0732	8.7077
5	2.4720(4)	<<	O4_b	[x,1-y,3/2-z	= 3566.01]		-60.00	31.02	0.23946	0.35574	0.85017	3.0453	4.5241	10.8119
6	2.4720(4)	<<	O4	[	= 01]		60.00	-31.02	0.23946	0.64426	0.64983	3.0453	8.1933	8.2641
7	2.5591(4)	<<	O3	[	= 01]		-15.34	-34.92	0.31529	0.45635	0.63482	4.0096	5.8036	8.0732
8	2.5591(4)	<<	O3_b	[x,1-y,3/2-z	= 3566.01]		15.34	34.92	0.31529	0.54365	0.86518	4.0096	6.9138	11.0028

Angles (Degrees) At1...V...At2 with Vertex V = Ca2

F2	F2_a	147.98(1)	F2	O3_d	72.62(1)	F2	O3_c	88.13(1)	F2	O4_b	95.88(1)
F2	O4	97.70(1)	F2	O3	69.51(1)	F2	O3_b	141.84(1)	F2_a	O3_d	88.13(1)
F2_a	O3_c	72.62(1)	F2_a	O4_b	97.70(1)	F2_a	O4	95.88(1)	F2_a	O3	141.84(1)
F2_a	O3_b	69.51(1)	O3_d	O3_c	106.18(1)	O3_d	O4_b	62.85(1)	O3_d	O4	166.17(1)
O3_d	O3	121.22(1)	O3_d	O3_b	115.57(1)	O3_c	O4_b	166.17(1)	O3_c	O4	62.85(1)
O3_c	O3	115.57(1)	O3_c	O3_b	121.22(1)	O4_b	O4	129.25(1)	O4_b	O3	78.18(1)
O4_b	O3_b	61.78(1)	O4	O3	61.78(1)	O4	O3_b	78.18(1)	O3	O3_b	75.49(1)

2.8 Angstrom Coordination Sphere Around Atom I = Si1 [ARU = 1555.01] 0.34509 0.57924 0.62366 4.3886 7.3664 7.9313

Nr	d(I,J)	To	Atom J	Symm_Oper. on Atom J	ARU(J)	Type	Phi	Mu	X	Y	Z	XO	YO	ZO
1	1.6122(4)	--	O4			Intra	148.38	11.91	0.23946	0.64426	0.64983	3.0453	8.1933	8.2641
2	1.6144(4)	--	O3			Intra	-103.63	5.04	0.31529	0.45635	0.63482	4.0096	5.8036	8.0732
3	1.6166(4)	--	O2			Intra	25.64	-62.85	0.39738	0.60434	0.51055	5.0536	7.6856	6.4928

```
 4   1.6288(4) --  O1                                    Intra  22.48  40.48   0.43511  0.61648  0.70680    5.5334  7.8400  8.9886

Tetrahedral Volume  2.158 Ang**3, Quadratic Elongation  1.005, Angle Variance  19.67 Deg**2 [K.Robinson +, Science 1971,172,567-570]

Tau(4)-Descriptor for 4-Coordination (L.Yang, D.R.Powell, R.P.Houser, Dalton Trans. (2007), 955-964)
-----------------------------------------------------------------------------------------------------------------------------------
Tau(4) = (360 - (Beta + Alpha)) / 141 = 0.94 :: (Extreme Forms: 0.00 for SQP and 1.00 for TET; 0.85 for TRP)

Angles (Degrees)  At1...V...At2 with Vertex V =  Si1
-----------------------------------------------------------------------------------------------------------------------------------
 O4     ,  O3        106.43(2)     O4     ,  O2        115.16(2)     O4     ,  O1        107.61(2)     O3     ,  O2        111.47(2)
 O3     ,  O1        112.92(2)     O2     ,  O1        103.36(2)
```



```
===================================================================================================================================
 2.8 Angstrom Coordination Sphere Around Atom I = F1     [ARU = 1555.02]         0.64547  0.64547  0.64547    8.2087  8.2087  8.2087
-----------------------------------------------------------------------------------------------------------------------------------
 Nr     d(I,J) To  Atom J  Symm_Oper. on Atom J    ARU(J)  Type     Phi     Mu         X         Y         Z        XO      YO      ZO
-----------------------------------------------------------------------------------------------------------------------------------
 1  1.8520(13) <<  Li1    [                    =       07]        45.00  35.26   0.72955  0.72955  0.72955    9.2779  9.2779  9.2779
 2   2.3979(4) <<  Ca1_b  [y,z,x               =  9555.04]       -29.38 -50.49       3/4  0.58662       1/2    9.5380  7.4602  6.3587
 3   2.3979(4) <<  Ca1_a  [z,x,y               =  5555.04]      -112.03  33.67   0.58662       1/2       3/4    7.4602  6.3587  9.5380
 4   2.3979(4) <<  Ca1    [                    =       04]       144.30 -18.19       1/2       3/4  0.58662    6.3587  9.5380  7.4602

Angles (Degrees)  At1...V...At2 with Vertex V =  F1
-----------------------------------------------------------------------------------------------------------------------------------
 Li1    ,  Ca1_b     107.79(4)     Li1    ,  Ca1_a     107.79(4)     Li1    ,  Ca1       107.79(4)     Ca1_b  ,  Ca1_a     111.10(2)
 Ca1_b  ,  Ca1       111.10(2)     Ca1_a  ,  Ca1       111.10(2)
```



```
===================================================================================================================================
```

2.8 Angstrom Coordination Sphere Around Atom I = F2 [ARU = 1555.03] 0.10478 0.39522 0.60478 1.3325 5.0261 7.6912

Nr	d(I,J)	To	Atom J	Symm_Oper. on Atom J	ARU(J)	Type	Phi	Mu	X	Y	Z	XO	YO	ZO
1	1.8113(12)	<<	<Li2	[	= 08]		135.00	-35.26	0.02255	0.47745	0.52255	0.2868	6.0719	6.6454
2	2.3693(4)	<<	Ca2	[	= 05]		63.88	51.21	0.15617	1/2	3/4	1.9860	6.3587	9.5380
3	2.3693(4)	<<	Ca2_a	[1-z,1/2-x,y	= 6655.05]		-19.49	-34.22	1/4	0.34383	1/2	3.1793	4.3726	6.3587
4	2.3693(4)	<<	Ca2_b	[-1/2+y,-1/2+z,1/2+x	= 21445.05]		-125.81	16.01	0	1/4	0.65617	-0.0000	3.1793	8.3447

Angles (Degrees) At1...V...At2 with Vertex V = F2

At1	At2	Angle
<Li2	Ca2	106.53(4)
<Li2	Ca2_a	106.53(4)
<Li2	Ca2_b	106.53(4)
Ca2	Ca2_a	112.24(2)
Ca2	Ca2_b	112.24(2)
Ca2_a	Ca2_b	112.24(2)

2.8 Angstrom Coordination Sphere Around Atom I = O1 [ARU = 1555.01] 0.43511 0.61648 0.70680 5.5334 7.8400 8.9886

Nr	d(I,J)	To	Atom J	Symm_Oper. on Atom J	ARU(J)	Type	Phi	Mu	X	Y	Z	XO	YO	ZO
1	1.6288(4)	--	Si1			Intra	-157.52	-40.48	0.34509	0.57924	0.62366	4.3886	7.3664	7.9313
2	1.6160(7)	<<	Be1_b	[x,1-y,3/2-z	= 3566.06]		155.39	75.29	0.40577	0.62992	0.82970	5.1603	8.0109	10.5516
3	2.4291(4)	<<	Ca1	[	= 04]		64.08	-38.99	1/2	3/4	0.58662	6.3587	9.5380	7.4602
4	2.4917(4)	<<	Ca1_c	[z,x,y	= 5555.04]		-37.55	12.74	0.58662	1/2	3/4	7.4602	6.3587	9.5380
5	2.5462(5)	<<	O2			Intra	-162.16	-78.58	0.39738	0.60434	0.51055	5.0536	7.6856	6.4928
6	2.5923(5)	<<	O2_d	[z,1-x,3/2-y	= 7566.01]		-10.41	67.90	0.51055	0.60262	0.89566	6.4928	7.6637	11.3904
7	2.6155(5)	<<	O4			Intra	171.92	-16.08	0.23946	0.64426	0.64983	3.0453	8.1933	8.2641
8	2.6834(5)	<<	O4_e	[1-z,1/2+x,3/2-y	= 8656.01]		124.63	44.90	0.35017	0.73946	0.85574	4.4532	9.4040	10.8827

```
 9   2.6901(5) <<  O3_a   [x,1-y,3/2-z         =  3566.01]      -148.71  48.48   0.31529  0.54365  0.86518    4.0096  6.9138 11.0028
10   2.7031(5) <<  O3                                    Intra-126.81 -19.79   0.31529  0.45635  0.63482    4.0096  5.8036  8.0732


Angles (Degrees)  At1...V...At2 with Vertex V =  O1
------------------------------------------------------------------------------------------------------------------------------------
 Si1    ,  Be1_b     119.76(3)     Si1    ,  Ca1         91.93(2)     Si1    ,  Ca1_c     120.92(2)     Si1    ,  O2          38.15(1)
 Si1    ,  O2_d      147.33(2)     Si1    ,  O4          35.98(1)     Si1    ,  O4_e      110.17(2)     Si1    ,  O3_a        89.30(2)
 Si1    ,  O3         33.37(1)     Be1_b  ,  Ca1        127.81(3)     Be1_b  ,  Ca1_c      91.61(3)     Be1_b  ,  O2         155.64(3)
 Be1_b  ,  O2_d       36.54(3)     Be1_b  ,  O4          91.95(3)     Be1_b  ,  O4_e       33.14(3)     Be1_b  ,  O3_a        35.06(3)
 Be1_b  ,  O3        106.09(3)     Ca1    ,  Ca1_c     106.96(1)     Ca1    ,  O2         59.31(1)     Ca1    ,  O2_d       120.32(2)
 Ca1    ,  O4         93.12(2)     Ca1    ,  O4_e       99.99(2)     Ca1    ,  O3_a      154.72(2)     Ca1    ,  O3         120.34(2)
 Ca1_c  ,  O2        109.01(2)     Ca1_c  ,  O2_d       57.94(1)     Ca1_c  ,  O4        151.28(2)     Ca1_c  ,  O4_e       120.14(2)
 Ca1_c  ,  O3_a       93.91(1)     Ca1_c  ,  O3          93.59(1)     O2     ,  O2_d      166.85(2)     O2     ,  O4          63.73(1)
 O2     ,  O4_e      130.65(2)     O2     ,  O3_a      127.32(2)     O2     ,  O3         61.06(1)     O2_d   ,  O4         128.16(2)
 O2_d   ,  O4_e       62.27(1)     O2_d   ,  O3_a       59.50(1)     O2_d   ,  O3        118.11(2)     O4     ,  O4_e        74.56(2)
 O4     ,  O3_a       73.44(2)     O4     ,  O3          58.11(1)     O4_e   ,  O3_a       56.23(1)     O4_e   ,  O3         116.82(2)
 O3_a   ,  O3         71.03(1)
```



```
====================================================================================================================================
 2.8 Angstrom Coordination Sphere Around Atom I = O2     [ARU = 1555.01]         0.39738  0.60434  0.51055    5.0536  7.6856  6.4928
------------------------------------------------------------------------------------------------------------------------------------
 Nr     d(I,J) To  Atom J  Symm_Oper. on Atom J    ARU(J)  Type     Phi      Mu         X         Y         Z         XO      YO      ZO
------------------------------------------------------------------------------------------------------------------------------------
 1   1.6166(4) --  Si1                                   Intra-154.36  62.85   0.34509  0.57924  0.62366    4.3886  7.3664  7.9313



 2   1.6124(7) <<  Be1_c  [y,z,x               =  9555.06]       112.48 -55.73   0.37008  0.67030  0.40577    4.7064  8.5244  5.1603
 3  1.9540(12) << <Li2_a  [1-z,1/2-x,y         =  6655.08]       -57.75 -12.44   0.47745  0.47745  0.47745    6.0719  6.0719  6.0719
 4   2.4638(4) <<  Ca1    [                    =       04]        54.83  23.12       1/2       3/4  0.58662    6.3587  9.5380  7.4602
```

```
 5   2.5462(5) <<  O1                                    Intra  17.84  78.58   0.43511  0.61648  0.70680    5.5334  7.8400  8.9886
 6   2.5923(5) <<  O1_d   [1-y,3/2-z,x         = 12665.01]        94.20 -21.72   0.38352  0.79320  0.43511    4.8773 10.0874  5.5334
 7   2.6228(5) <<  O3_b   [y,z,x               =  9555.01]        27.33 -71.22   0.45635  0.63482  0.31529    5.8036  8.0732  4.0096
 8   2.6701(5) <<  O3                                    Intra-119.02  36.29   0.31529  0.45635  0.63482    4.0096  5.8036  8.0732
 9   2.7255(5) <<  O4                                    Intra 165.81  40.53   0.23946  0.64426  0.64983    3.0453  8.1933  8.2641
10   2.7288(5) <<  O4_e   [1/2-x,y,1-z         = 16556.01]       163.74 -48.37   0.26054  0.64426  0.35017    3.3134  8.1933  4.4532

Angles (Degrees)  At1...V...At2 with Vertex V =  O2
-----------------------------------------------------------------------------------------------------------------------------------
 Si1    ,  Be1_c     138.55(3)     Si1    , <Li2_a     104.06(4)     Si1    ,  Ca1         90.97(2)     Si1    ,  O1           38.49(1)
 Si1    ,  O1_d      118.97(2)     Si1    ,  O3_b      171.60(2)     Si1    ,  O3          34.24(1)     Si1    ,  O4           32.37(1)
 Si1    ,  O4_e      116.07(2)     Be1_c  , <Li2_a     111.34(5)     Be1_c  ,  Ca1         92.72(3)     Be1_c  ,  O1          145.00(3)
 Be1_c  ,  O1_d       36.63(3)     Be1_c  ,  O3_b       37.08(3)     Be1_c  ,  O3         140.50(3)     Be1_c  ,  O4          106.35(3)
 Be1_c  ,  O4_e       31.59(3)    <Li2_a  ,  Ca1       115.43(4)    <Li2_a  ,  O1          99.38(4)    <Li2_a  ,  O1_d        136.13(4)
<Li2_a  ,  O3_b       76.65(4)    <Li2_a  ,  O3         75.47(4)    <Li2_a  ,  O4         132.67(4)    <Li2_a  ,  O4_e        108.96(4)
 Ca1    ,  O1         57.98(1)     Ca1    ,  O1_d       58.99(1)     Ca1    ,  O3_b        96.27(2)     Ca1    ,  O3          120.31(2)
 Ca1    ,  O4         89.72(1)     Ca1    ,  O4_e      119.43(2)     O1     ,  O1_d       108.63(2)     O1     ,  O3_b        149.91(2)
 O1     ,  O3         62.37(1)     O1     ,  O4         59.37(1)     O1     ,  O4_e       147.31(2)     O1_d   ,  O3_b         62.10(1)
 O1_d   ,  O3        147.73(2)     O1_d   ,  O4         91.02(2)     O1_d   ,  O4_e        60.50(1)     O3_b   ,  O3          140.93(2)
 O3_b   ,  O4        142.98(2)     O3_b   ,  O4_e       56.44(1)     O3     ,  O4          57.22(1)     O3     ,  O4_e        108.92(2)
 O4     ,  O4_e       88.92(2)
```



```
===================================================================================================================================
 2.8 Angstrom Coordination Sphere Around Atom I = O3     [ARU = 1555.01]         0.31529  0.45635  0.63482    4.0096  5.8036  8.0732
-----------------------------------------------------------------------------------------------------------------------------------
 Nr     d(I,J) To  Atom J  Symm_Oper. on Atom J    ARU(J)  Type      Phi     Mu          X        Y        Z         XO      YO      ZO
-----------------------------------------------------------------------------------------------------------------------------------
 1   1.6144(4) --  Si1                                   Intra  76.37  -5.04   0.34509  0.57924  0.62366    4.3886  7.3664  7.9313
```

Nr	d(I,J)	To	Atom J	Symm_Oper. on Atom J	ARU(J)	Type	Phi	Mu	X	Y	Z	XO	YO	ZO
2	1.6527(7)	<<	Be1	[	= 06]		-43.64	15.84	0.40577	0.37008	0.67030	5.1603	4.7064	8.5244
3	2.3826(4)	<<	Ca2_c	[1-z,1/2-x,y	= 6655.05]		-120.13	-46.02	1/4	0.34383	1/2	3.1793	4.3726	6.3587
4	2.5322(5)	<<	O4_d	[1-z,1/2-x,y	= 6655.01]		-79.90	2.72	0.35017	0.26054	0.64426	4.4532	3.3134	8.1933
5	2.5591(4)	<<	Ca2	[	= 05]		164.66	34.92	0.15617	1/2	3/4	1.9860	6.3587	9.5380
6	2.5840(5)	<<	O4			Intra	111.98	4.24	0.23946	0.64426	0.64983	3.0453	8.1933	8.2641
7	2.6228(5)	<<	O2_b	[z,x,y	= 5555.01]		-16.80	-8.50	0.51055	0.39738	0.60434	6.4928	5.0536	7.6856
8	2.6701(5)	<<	O2			Intra	60.98	-36.29	0.39738	0.60434	0.51055	5.0536	7.6856	6.4928
9	2.6901(5)	<<	O1_a	[x,1-y,3/2-z	= 3566.01]		-31.29	48.48	0.43511	0.38352	0.79320	5.5334	4.8773	10.0874
10	2.7031(5)	<<	O1			Intra	53.19	19.79	0.43511	0.61648	0.70680	5.5334	7.8400	8.9886

Angles (Degrees) At1...V...At2 with Vertex V = O3

Si1	,	Be1	120.21(3)	Si1	,	Ca2_c	126.87(2)	Si1	,	O4_d	156.21(2)	Si1	,	Ca2	91.49(2)
Si1	,	O4	36.76(1)	Si1	,	O2_b	92.38(2)	Si1	,	O2	34.29(1)	Si1	,	O1_a	105.44(2)
Si1	,	O1	33.71(1)	Be1	,	Ca2_c	92.32(3)	Be1	,	O4_d	38.03(3)	Be1	,	Ca2	122.57(3)
Be1	,	O4	148.61(3)	Be1	,	O2_b	36.03(2)	Be1	,	O2	110.93(3)	Be1	,	O1_a	34.17(2)
Be1	,	O1	90.87(3)	Ca2_c	,	O4_d	60.30(1)	Ca2_c	,	Ca2	105.46(1)	Ca2_c	,	O4	118.59(2)
Ca2_c	,	O2_b	92.97(2)	Ca2_c	,	O2	97.68(2)	Ca2_c	,	O1_a	121.97(2)	Ca2_c	,	O1	153.20(2)
O4_d	,	Ca2	108.95(2)	O4_d	,	O4	166.25(2)	O4_d	,	O2_b	63.89(1)	O4_d	,	O2	130.75(2)
O4_d	,	O1_a	61.75(1)	O4_d	,	O1	128.76(2)	Ca2	,	O4	57.45(1)	Ca2	,	O2_b	153.55(2)
Ca2	,	O2	119.67(2)	Ca2	,	O1_a	95.40(1)	Ca2	,	O1	95.08(1)	O4	,	O2_b	128.95(2)
O4	,	O2	62.47(1)	O4	,	O1_a	118.33(2)	O4	,	O1	59.25(1)	O2_b	,	O2	75.16(2)
O2_b	,	O1_a	58.39(1)	O2_b	,	O1	74.44(2)	O2	,	O1_a	117.67(2)	O2	,	O1	56.57(1)
O1_a	,	O1	71.73(1)												

2.8 Angstrom Coordination Sphere Around Atom I = O4 [ARU = 1555.01] 0.23946 0.64426 0.64983 3.0453 8.1933 8.2641

Nr	d(I,J)	To	Atom J	Symm_Oper. on Atom J	ARU(J)	Type	Phi	Mu	X	Y	Z	XO	YO	ZO

1	1.6122(4)	--	Si1			Intra	-31.62	-11.91	0.34509	0.57924	0.62366	4.3886	7.3664	7.9313
2	1.5970(7)	<<	Be1_e	[1/2-y,z,1-x	= 23556.06]		166.63	-26.28	0.12992	0.67030	0.59423	1.6522	8.5244	7.5570
3	1.9387(13)	<<	Li1_a	[1-x,3/2-y,z	= 2665.07]		76.20	31.53	0.27045	0.77045	0.72955	3.4394	9.7981	9.2779
4	2.4720(4)	<<	Ca2	[	= 05]		-120.00	31.02	0.15617	1/2	3/4	1.9860	6.3587	9.5380
5	2.5322(5)	<<	O3_d	[1/2-y,z,1-x	= 23556.01]		-177.24	10.09	0.04365	0.63482	0.68471	0.5551	8.0732	8.7077
6	2.5840(5)	<<	O3			Intra	-68.02	-4.24	0.31529	0.45635	0.63482	4.0096	5.8036	8.0732
7	2.6155(5)	<<	O1			Intra	-8.08	16.08	0.43511	0.61648	0.70680	5.5334	7.8400	8.9886
8	2.6834(5)	<<	O1_c	[-1/2+y,3/2-z,1-x	= 22466.01]		129.55	-23.74	0.11648	0.79320	0.56489	1.4813	10.0874	7.1839
9	2.7255(5)	<<	O2			Intra	-14.19	-40.53	0.39738	0.60434	0.51055	5.0536	7.6856	6.4928
10	2.7288(5)	<<	O2_b	[1/2-x,y,1-z	= 16556.01]		-163.74	-48.37	0.10262	0.60434	0.48945	1.3051	7.6856	6.2245

Angles (Degrees) At1...V...At2 with Vertex V = O4

Si1	Be1_e	137.89(3)	Si1	Li1_a	111.29(4)	Si1	Ca2	94.75(2)	Si1	O3_d	146.23(2)
Si1	O3	36.82(1)	Si1	O1	36.41(1)	Si1	O1_c	139.87(2)	Si1	O2	32.47(1)
Si1	O2_b	106.36(2)	Be1_e	Li1_a	103.72(5)	Be1_e	Ca2	90.47(3)	Be1_e	O3_d	39.61(3)
Be1_e	O3	118.99(3)	Be1_e	O1	168.68(3)	Be1_e	O1_c	33.59(3)	Be1_e	O2	113.18(3)
Be1_e	O2_b	31.93(3)	Li1_a	Ca2	115.59(4)	Li1_a	O3_d	98.49(4)	Li1_a	O3	136.74(4)
Li1_a	O1	76.91(4)	Li1_a	O1_c	75.21(4)	Li1_a	O2	110.14(4)	Li1_a	O2_b	132.42(4)
Ca2	O3_d	56.85(1)	Ca2	O3	60.77(1)	Ca2	O1	99.48(2)	Ca2	O1_c	118.79(2)
Ca2	O2	120.83(2)	Ca2	O2_b	88.50(1)	O3_d	O3	109.64(2)	O3_d	O1	151.72(2)
O3_d	O1_c	62.02(1)	O3_d	O2	146.06(2)	O3_d	O2_b	59.67(1)	O3	O1	62.65(1)
O3	O1_c	147.20(2)	O3	O2	60.31(1)	O3	O2_b	90.62(2)	O1	O1_c	139.58(2)
O1	O2	56.90(1)	O1	O2_b	142.06(2)	O1_c	O2	107.42(2)	O1_c	O2_b	57.23(1)
O2	O2_b	87.11(2)									

2.8 Angstrom Coordination Sphere Around Atom I = Be1 [ARU = 1555.06] 0.40577 0.37008 0.67030 5.1603 4.7064 8.5244

Nr	d(I,J)	To	Atom J	Symm_Oper. on Atom J	ARU(J)	Type	Phi	Mu	X	Y	Z	XO	YO	ZO
1	1.5970(7)	<<	O4_d	[1-z,1/2-x,y	= 6655.01]		-116.91	-11.97	0.35017	0.26054	0.64426	4.4532	3.3134	8.1933
2	1.6124(7)	<<	O2_c	[z,x,y	= 5555.01]		14.60	-31.35	0.51055	0.39738	0.60434	6.4928	5.0536	7.6856
3	1.6160(7)	<<	O1_a	[x,1-y,3/2-z	= 3566.01]		24.61	75.29	0.43511	0.38352	0.79320	5.5334	4.8773	10.0874
4	1.6527(7)	<<	O3	[	= 01]		136.36	-15.84	0.31529	0.45635	0.63482	4.0096	5.8036	8.0732
5	2.7888(14)	.<	Li1_b	[1-x,-1/2+y,3/2-z	= 4646.07]		-133.92	27.17	0.27045	0.22955	0.77045	3.4394	2.9193	9.7981

Angles (Degrees) At1...V...At2 with Vertex V = Be1

O4_d ,	O2_c	116.48(4)	O4_d ,	O1_a	113.27(4)	O4_d ,	O3	102.37(4)	O4_d ,	Li1_b	42.48(3)
O2_c ,	O1_a	106.83(4)	O2_c ,	O3	106.88(4)	O2_c ,	Li1_b	152.32(5)	O1_a ,	O3	110.77(4)
O1_a ,	Li1_b	76.62(4)	O3 ,	Li1_b	96.92(4)						

2.8 Angstrom Coordination Sphere Around Atom I = Li1 [ARU = 1555.07] 0.72955 0.72955 0.72955 9.2779 9.2779 9.2779

Nr	d(I,J)	To	Atom J	Symm_Oper. on Atom J	ARU(J)	Type	Phi	Mu	X	Y	Z	XO	YO	ZO
1	1.8520(13)	<<	F1	[	= 02]		-135.00	-35.26	0.64547	0.64547	0.64547	8.2087	8.2087	8.2087
2	1.9387(13)	<<	O4_a	[1-x,3/2-y,z	= 2665.01]		76.20	-31.53	0.76054	0.85574	0.64983	9.6720	10.8827	8.2641
3	1.9387(13)	<<	O4_c	[z,1-x,3/2-y	= 7566.01]		158.76	55.87	0.64983	0.76054	0.85574	8.2641	9.6720	10.8827
4	1.9387(13)	<<	O4_f	[3/2-y,z,1-x	= 23656.01]		-32.28	11.73	0.85574	0.64983	0.76054	10.8827	8.2641	9.6720
5	2.7888(14)	.<	Be1_b	[1-x,1/2+y,3/2-z	= 4656.06]		133.92	27.17	0.59423	0.87008	0.82970	7.5570	11.0651	10.5516
6	2.7888(14)	.<	Be1_d	[3/2-z,1-x,1/2+y	= 18665.06]		-53.49	39.85	0.82970	0.59423	0.87008	10.5516	7.5570	11.0651
7	2.7888(14)	.<	Be1_e	[1/2+y,3/2-z,1-x	= 22566.06]		35.48	-38.10	0.87008	0.82970	0.59423	11.0651	10.5516	7.5570

Angles (Degrees) At1...V...At2 with Vertex V = Li1

At1	At2	Angle	At1	At2	Angle	At1	At2	Angle	At1	At2	Angle
F1	O4_a	107.06(6)	F1	O4_c	107.06(6)	F1	O4_f	107.06(6)	F1	Be1_b	106.10(6)
F1	Be1_d	106.10(6)	F1	Be1_e	106.10(6)	O4_a	O4_c	111.77(6)	O4_a	O4_f	111.77(6)
O4_a	Be1_b	80.43(5)	O4_a	Be1_d	138.86(6)	O4_a	Be1_e	33.80(3)	O4_c	O4_f	111.77(6)
O4_c	Be1_b	33.80(3)	O4_c	Be1_d	80.43(5)	O4_c	Be1_e	138.86(6)	O4_f	Be1_b	138.86(6)
O4_f	Be1_d	33.80(3)	O4_f	Be1_e	80.43(5)	Be1_b	Be1_d	112.62(5)	Be1_b	Be1_e	112.62(5)
Be1_d	Be1_e	112.62(5)									

2.8 Angstrom Coordination Sphere Around Atom I = Li2   [ARU = 1555.08]   0.02255 0.47745 0.52255   0.2868 6.0719 6.6454

Nr	d(I,J)	To	Atom J	Symm_Oper. on Atom J	ARU(J)	Type	Phi	Mu	X	Y	Z	XO	YO	ZO
1	1.8113(12)	<<	F2	[	= 03]		-45.00	35.26	0.10478	0.39522	0.60478	1.3325	5.0261	7.6912
2	1.9540(12)	<<	O2_b	[1/2-z,x,1-y	= 20556.01]		-112.46	-55.67	-0.01055	0.39738	0.39566	-0.1342	5.0536	5.0317
3	1.9540(12)	<<	O2_a	[1/2-x,y,1-z	= 16556.01]		57.75	-12.44	0.10262	0.60434	0.48945	1.3051	7.6856	6.2245
4	1.9540(12)	<<	O2_c	[1/2-y,z,1-x	= 23556.01]		165.38	31.41	-0.10434	0.51055	0.60262	-1.3269	6.4928	7.6637

Angles (Degrees) At1...V...At2 with Vertex V = <Li2

At1	At2	Angle	At1	At2	Angle	At1	At2	Angle	At1	At2	Angle
F2	O2_b	107.48(6)	F2	O2_a	107.48(6)	F2	O2_c	107.48(6)	O2_b	O2_a	111.39(6)
O2_b	O2_c	111.39(6)	O2_a	O2_c	111.39(6)						

References

1. Hansen, N. K. & Coppens, P. Testing aspherical atom refinements on small-molecule data sets. *Acta Crystallographica Section A: Crystal Physics, Diffraction, Theoretical and General Crystallography* **34**, 909–921 (1978).
2. Koritsanszky, T. S. & Coppens, P. Chemical applications of X-ray charge-density analysis. *Chemical reviews* **101**, 1583–1628 (2001).
3. Su, Z. & Coppens, P. Nonlinear Least-Squares Fitting of Numerical Relativistic Atomic Wave Functions by a Linear Combination of Slater-Type Functions for Atoms with *Z* = 1–36. *Acta Crystallographica Section A Foundations of Crystallography* **54**, 646–652 (1998).
4. Bianchi, R., Forni, A. & Oberti, R. Multipole-refined charge density study of diopside at ambient conditions. *Phys Chem Minerals* **32**, 638–645 (2005).
5. Kuntzinger, S. & Ghermani, N. E. Electron density distribution and Madelung potential in α-spodumene, LiAl(SiO3)2, from two-wavelength high-resolution X-ray diffraction data. *Acta Crystallographica Section B Structural Science* **55**, 273–284 (1999).
6. Kuntzinger, S., Dahaoui, S., Ghermani, N. E., Lecomte, C. & Howard, J. A. K. The use of CCD area detectors in charge-density research. Application to a mineral compound: the α-spodumene $LiAl(SiO_3)_2$. *Acta Crystallographica Section B Structural Science* **55**, 867–881 (1999).
7. Ghermani, N. E., Lecomte, C. & Dusausoy, Y. Electrostatic properties in zeolite-type materials from high-resolution x-ray diffraction: The case of natrolite. *Phys. Rev. B* **53**, 5231–5239 (1996).
8. Kuntzinger, S., Ghermani, N. E., Dusausoy, Y. & Lecomte, C. Distribution and Topology of the Electron Density in an Aluminosilicate Compound from High-Resolution X-ray Diffraction Data: the Case of Scolecite. *Acta Crystallographica Section B Structural Science* **54**, 819–833 (1998).
9. Downs, J. W. & Gibbs, G. V. An exploratory examination of the electron density and electrostatic potential of phenakite. *American Mineralogist* **72**, 769–777 (1987).
10. Tsirelson, V. G., Evdokimova, O. A., Belokoneva, E. L. & Urusov, V. S. Electron Density distribution and bonding in silicates. *Physics and Chemistry of Minerals* **17**, 275–292 (1990).
11. Gibbs, G. V. *et al.* An Exploration of Theoretical and Experimental Electron Density Distributions and SiO Bonded Interactions for the Silica Polymorph Coesite. *J. Phys. Chem. B* **107**, 12996–13006 (2003).
12. Kirfel, A., Krane, H. G., Blaha, P., Schwarz, K. & Lippmann, T. Electron-density distribution in stishovite, SiO2: a new high-energy synchrotron-radiation study. *Acta Crystallogr., A, Found. Crystallogr.* **57**, 663–677 (2001).
13. Jiang, B., Zuo, J. M., Jiang, N., O'Keeffe, M. & Spence, J. C. H. Charge density and chemical bonding in rutile, TiO_2. *Acta Crystallographica Section A Foundations of Crystallography* **59**, 341–350 (2003).
14. Restori, R., Schwarzenbach, D. & Schneider, J. R. Charge density in rutile, TiO_2. *Acta Crystallographica Section B Structural Science* **43**, 251–257 (1987).
15. Belokoneva, E. L., Gubina, Y. K. & Forsyth, J. B. The charge density distribution and antiferromagnetic properties of azurite Cu3[CO3]2(OH)2. *Phys Chem Min* **28**, 498–507 (2001).
16. Restori, R. & Schwarzenbach, D. Charge density in cuprite, Cu_2O. *Acta Crystallographica Section B Structural Science* **42**, 201–208 (1986).
17. Zuo, J. M., Kim, M., O'Keeffe, M. & Spence, J. C. H. Direct observation of d-orbital holes and Cu–Cu bonding in Cu2O. *Nature* **401**, 49–52 (1999).
18. Ivanov, Yu. V., Belokoneva, E. L., Protas, J., Hansen, N. K. & Tsirelson, V. G. Multipole Analysis of the Electron Density in Topaz Using X-ray Diffraction Data. *Acta Crystallographica Section B Structural Science* **54**, 774–781 (1998).
19. Ivanov, Yu. V. & Belokoneva, E. L. Multipole refinement and electron density analysis in natural borosilicate datolite using X-ray diffraction data. *Acta Crystallographica Section B* **63**, 49–55 (2007).

20. Belokoneva, E. L., Gubina, Yu. K., Forsyth, J. B. & Brown, P. J. The charge-density distribution, its multipole refinement and the antiferromagnetic structure of dioptase, Cu6[Si6O18]·6H2O. *Physics and Chemistry of Minerals* **29**, 430–438 (2002).
21. Hill, R. J. Crystal structure refinement and electron density distribution in diaspore. *Physics and Chemistry of Minerals* **5**, 179–200 (1979).
22. Streltsov, V. A., Belokoneva, E. L., Tsirelson, V. G. & Hansen, N. K. Multipole analysis of the electron density in triphylite, LiFePO4, using X-ray diffraction data. *Acta Crystallographica Section B: Structural Science* **49**, 147–153 (1993).
23. Maslen, E. N., Streltsov, V. A., Streltsova, N. R. & Ishizawa, N. Synchrotron X-ray study of the electron density in α-Fe2O3. *Acta Crystallographica Section B: Structural Science* **50**, 435–441 (1994).
24. Gonschorek, W., Schmahl, W. W., Weitzel, H., Miehe, G. & Fuess, H. Anharmonic motion and multipolar expansion of the electron density in $NaNO_3$. *Zeitschrift für Kristallographie-Crystalline Materials* **210**, 843–849 (1995).
25. Kirfel, A. & Gibbs, G. V. Electron density distributions and bonded interactions for the fibrous zeolites natrolite, mesolite and scolecite and related materials. *Physics and Chemistry of Minerals* **27**, 270–284 (2000).
26. Pillet, S. *et al.* Recovering experimental and theoretical electron densities in corundum using the multipolar model: IUCr multipole refinement project. *Acta Crystallographica Section A: Foundations of Crystallography* **57**, 290–303 (2001).
27. Merli, M., Pavese, A. & Ranzini, M. Study of the electron density in MgO,$(Mg_{0.963}Fe_{0.037})O$ and Cu_2O by the maximum entropy method and multipole refinements: comparison between methods. *Physics and chemistry of minerals* **29**, 455–464 (2002).
28. Kirfel, A. *et al.* Electron density distribution and bond critical point properties for forsterite, Mg_2SiO_4, determined with synchrotron single crystal X-ray diffraction data. *Physics and chemistry of minerals* **32**, 301–313 (2005).
29. Kurylyshyn, I. M. *et al.* Probing the Zintl–Klemm concept: a combined experimental and theoretical charge density study of the Zintl phase CaSi. *Angewandte Chemie International Edition* **53**, 3029–3032 (2014).
30. Stachowicz, M., Malinska, M., Parafiniuk, J. & Woźniak, K. Experimental observation of charge-shift bond in fluorite CaF_2. *Acta Crystallographica Section B Structural Science, Crystal Engineering and Materials* **73**, 643–653 (2017).
31. Nelyubina, Y. V., Korlyukov, A. A., Lyssenko, K. A. & Fedyanin, I. V. Transferable Aspherical Atom Modeling of Electron Density in Highly Symmetric Crystals: A Case Study of Alkali-Metal Nitrates. *Inorganic Chemistry* **56**, 4688–4696 (2017).
32. Gajda, R. *et al.* Experimental charge density of grossular under pressure – a feasibility study. *IUCrJ* **7**, (2020).
33. Hirshfeld, F. L. Bonded-atom fragments for describing molecular charge densities. *Theoret. Chim. Acta* **44**, 129–138 (1977).
34. Bader, R. F. W. *Atoms in Molecules: A Quantum Theory*. (Clarendon Press, 1994).
35. Popelier, P. L. A. Integration of atoms in molecules: a critical examination. *Molecular Physics* **87**, 1169–1187 (1996).
36. Gatti, C. Chemical bonding in crystals: new directions. *Zeitschrift für Kristallographie - Crystalline Materials* **220**, 399–457 (2005).
37. Coppens, P. *et al.* Net atomic charges and molecular dipole moments from spherical-atom X-ray refinements, and the relation between atomic charge and shape. *Acta Crystallographica Section A: Crystal Physics, Diffraction, Theoretical and General Crystallography* **35**, 63–72 (1979).
38. Ángyán, J. G., Jansen, G., Loss, M., Hättig, C. & Heß, B. A. Distributed polarizabilities using the topological theory of atoms in molecules. *Chemical Physics Letters* **219**, 267–273 (1994).

39. Dominiak, P. M. *et al.* Continua of interactions between pairs of atoms in molecular crystals. *Chemistry–A European Journal* **12**, 1941–1949 (2006).
40. Pauling, L. The principles determining the structure of complex ionic crystals. *Journal of the american chemical society* **51**, 1010–1026 (1929).
41. Brown, I. D. *The Chemical Bond in Inorganic Chemistry: The Bond Valence Model*. (International Union of Crystallography Monographs on Crystallography 12, 2001).
